# Structure–fluorescence activation relationships of a large Stokes shift fluorogenic RNA aptamer

**DOI:** 10.1093/nar/gkz1084

**Published:** 2019-11-19

**Authors:** Christian Steinmetzger, Irene Bessi, Ann-Kathrin Lenz, Claudia Höbartner

**Affiliations:** Institute of Organic Chemistry, Julius-Maximilians-University Würzburg, Am Hubland, 97074 Würzburg, Germany

## Abstract

The Chili RNA aptamer is a 52 nt long fluorogen-activating RNA aptamer (FLAP) that confers fluorescence to structurally diverse derivatives of fluorescent protein chromophores. A key feature of Chili is the formation of highly stable complexes with different ligands, which exhibit bright, highly Stokes-shifted fluorescence emission. In this work, we have analyzed the interactions between the Chili RNA and a family of conditionally fluorescent ligands using a variety of spectroscopic, calorimetric and biochemical techniques to reveal key structure–fluorescence activation relationships (SFARs). The ligands under investigation form two categories with emission maxima of ∼540 or ∼590 nm, respectively, and bind with affinities in the nanomolar to low-micromolar range. Isothermal titration calorimetry was used to elucidate the enthalpic and entropic contributions to binding affinity for a cationic ligand that is unique to the Chili aptamer. In addition to fluorescence activation, ligand binding was also observed by NMR spectroscopy, revealing characteristic signals for the formation of a G-quadruplex only upon ligand binding. These data shed light on the molecular features required and responsible for the large Stokes shift and the strong fluorescence enhancement of red and green emitting RNA–chromophore complexes.

## INTRODUCTION

In the wake of the seminal report of the Spinach RNA aptamer (1), fluorogen-activating aptamers (FLAPs) have found their footing as biochemical tools for studying RNA in vitro and in cells. As RNA labeling tools, FLAPs supplement the multitude of available fluorescent proteins in live-cell imaging applications (2–9). While fluorescent proteins exist as monolithic units of a fluorophore covalently embedded in the protein, fluorogenic aptamers offer the opportunity for dual engineering of chromophore and RNA due to the non-covalent nature of the interaction between the nucleic acid and its ligand. The different classes of known FLAP chromophores include derivatives of 4-hydroxybenzylidene imidazolon (HBI) (1,10,11), triphenylmethane dyes (12), cyanine dyes (9,13–15) and rhodamines (16,17) including silicon rhodamine (SiR) (18). Despite the large variety of reported FLAPs, only few have been thoroughly characterized with respect to structure–fluorescence activation relationships and ligand diversity (3,17,19).

The structural features responsible for fluorescence activation by several HBI-binding RNA aptamers are well documented on the basis of X-ray co-crystal structures with their respective ligands (3,20–22). Spinach and its variants (Spinach2 (23), Baby Spinach (24), iSpinach (25), Broccoli (26)) that activate the fluorescence of 3,5-difluoro-4-hydroxybenzylidene imidazolone (DFHBI), as well as Corn, a dimeric RNA aptamer for the yellow-emitting dye 3,5-difluoro-4-hydroxybenzylidene imidazolone oxime (DFHO) (11), adopt quadruplex folds composed of two G-quartets and one or more mixed-sequence quartets. Spinach contains a U–A–U base triple on top of a G-quartet with the ligand intercalated in between the two layers (24,27,28). Such a base triple is absent in Corn, which forms a homodimer that encloses the ligand at the interface of the two individual quadruplexes (29). In both aptamers a confined environment is created that decreases the nonradiative deactivation rate of bound photoexcited HBI and results in a significant fluorescence turn-on.

Despite a recently reported exception (30), G-quadruplexes are suggested to be a privileged tertiary structure motif for fluorogen-activating aptamers (20–22) and were also found in the family of Mango RNAs, which activate the Thiazole orange conjugate TO1-biotin (13–15). In Mango aptamers, three tiers of G-quartets are arranged in an unusual mixed parallel–antiparallel topology and undergo van der Waals interactions with the entire fluorophore-linker-biotin unit (31,32). Even though the nature of the binding site and the mode of interaction are unlike those found in Spinach–DFHBI and Corn–DFHO, conceptionally the same effect of decreased nonradiative fluorophore deactivation is achieved.

Apart from the core structure of the binding site and the chromophore, ancillary nucleobases as well as ligand side chains can also play important roles in determining the fluorescence properties of the RNA–ligand complexes. For example, quantum yield and binding affinity of Spinach2 were tuned by the introduction of trifluoromethyl groups to the parent ligand 3,5-difluoro-4-hydroxybenzylidene imidazolone (DFHBI) to generate DFHBI-1T (10). In DFHO-binding variants of Broccoli, mutation of an adenosine in close proximity to the ligand was shown to shift the emission maximum over a range of 20 nm. This effect was proposed to be caused by specific hydrogen bonding interactions between the oxime moiety of DFHO and the adjacent nucleobase. DFHBI, lacking the oxime substituent, was unaffected by any mutation at that position (33).

Recently, the 52-nt RNA Chili has been reported to exhibit Stokes shifts on the order of 130 nm when inducing fluorescence in HBI-derived dyes. Chili is an engineered version of the 13–2 RNA aptamer, which was in vitro-selected to bind 4-hydroxy-3,5-dimethoxybenzylidene imidazolone (DMHBI, **1**) (1). Compared to 13–2, Chili shows brighter fluorescence and tighter binding with low-nanomolar affinities, as well as greatly reduced Mg^2+^ requirements for fluorescence enhancement of DMHBI and extensively modified HBI ligands (34). Here, we present a comprehensive survey of the performance of the Chili aptamer with various ligand modifications and RNA point mutations. The ligand structure was altered by introduction of additional H-bonding sites, expansion of the conjugated π electron system and addition of a cationic side chain. NMR and variable temperature optical spectroscopy revealed the strictly ligand-dependent folding of a G-quadruplex domain in the Chili RNA. Several green and red emitting RNA–chromophore complexes were discovered that exhibit bright fluorescence enhancement and high binding affinity and revealed novel structure-fluorescence activation relationships.

## MATERIALS AND METHODS

### RNA synthesis and folding

The Chili RNA aptamer and its derivatives were prepared by in vitro transcription with T7 RNA polymerase using synthetic DNA templates (see Supplementary Table S4 for sequence information). RNAs were purified by denaturing polyacrylamide gel electrophoresis, extraction and precipitation. The purity of the transcripts was checked by anion exchange HPLC and concentrations were determined by measuring UV absorbance at 260 nm. For RNA folding, the aqueous RNA solution was heated to 95°C for 3 min in a buffer containing KCl and HEPES and then kept at 20°C for 20 min before MgCl_2_ was added. The final composition of the binding buffer was 125 mm KCl, 40 or 80 mm HEPES pH 7.5 and 5 mm MgCl_2_.

### Fluorescence screening

The RNA was annealed as described above to make a 1 μm solution in binding buffer. In parallel, a 1 μm dye solution in binding buffer was prepared. 7.5 μl of each solution were mixed in a 3 × 3 mm cuvette and incubated at 20°C for 3 min prior to measurement. Measurements were repeated after 24 h to check for time-dependent changes. Excitation and emission spectra were collected at the optimum wavelengths for each RNA–dye combination using otherwise identical instrument settings. Background spectra for blank subtraction were obtained from samples containing no RNA. The resulting emission spectra were integrated over the whole peak width and the resulting intensities were normalized to the Chili complex of DMHBI.

### Fluorescence titration

A dilution series (15 steps) was prepared from a 2× solution of the Chili aptamer and the RNA was annealed as described above. Titration samples in binding buffer were obtained by adding a 4× solution of the dye and MgCl_2_ and were incubated at 4°C for 16 h prior to measurement. Emission spectra were collected at the optimum wavelength for each RNA-dye combination. Background spectra for blank subtraction were obtained from pure binding buffer. The resulting emission spectra were integrated over the whole peak width and then fitted to a single site binding model.

### Isothermal titration calorimetry

A 150 μm stock solution of RNA was dialyzed against ultra pure H_2_O according to standard protocols. This solution was diluted and annealed as described above to obtain a 300 μl sample containing 10 or 15 μm RNA in binding buffer, which was loaded into the measurement cell of the calorimeter. In parallel, a 100 or 150 μm dye sample in binding buffer was prepared in an identical manner and loaded into the syringe of the calorimeter. The dye was titrated into the RNA at 25°C using the default protocol for 13 or 17 injections. Background heat measurements for baseline correction were obtained by titrating the dye into an identically prepared buffer containing no RNA. The data points were fitted to a single site binding model as implemented in the MicroCal PEAQ-ITC analysis software.

### Binding kinetics

The RNA was annealed as described above to make a 26 nM solution in 1.05× binding buffer. In parallel, solutions of the dye (16, 21, 32, 42 and 53 μm) in water were prepared. Under constant stirring, 20 μl of the respective dye solution were rapidly injected into 400 μl of the RNA solution at 25°C. Fluorescence time courses were monitored for 10–30 min at the optimum wavelengths for each RNA–dye combination. The data points were fitted to a biexponential association model.

### Thermal melting analysis

The RNA was heated to 95°C for 3 min in a buffer containing KCl and HEPES and then kept at 20°C for 20 min before adding DMHBI^+^ to make a solution containing 2 μm RNA, 2 μm ligand 125 mm KCl and 40 mm HEPES pH 7.5. The absorbance of the solution at 260 and 295 nm was measured in a 1 cm cuvette, collecting four temperature ramps between 10 and 95°C with a heating rate of 0.5°C/min. Afterwards, the fluorescence intensity (Ex/Em: 413/542 nm) of the sample was measured in a 10 × 2 mm cuvette using identical temperature ramps. A sample containing no ligand served as a control.

Full details of the synthesis and characterization of HBI derivatives, NMR measurements and computational methods are given in the Supporting Information.

## RESULTS AND DISCUSSION

### Synthesis of fluorogenic HBI derivatives

**Scheme 1.**
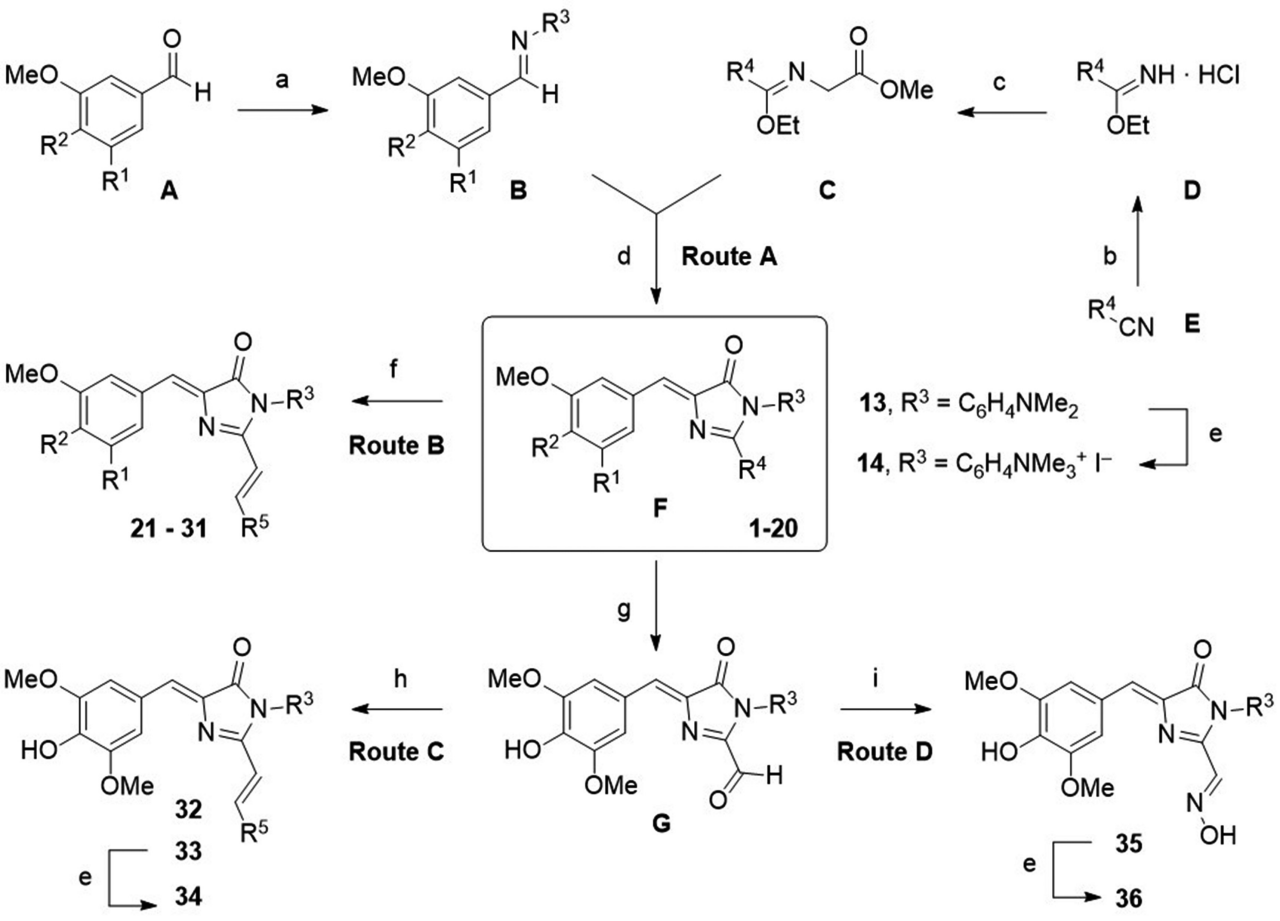
Synthesis of functionalized HBI scaffolds (Route A) and their π-extended derivatives (Route B–D). Reagents and conditions: (**a**) R^3^NH_2_, MgSO_4_, CH_2_Cl_2_, r.t., 24 h; or R^3^NH_2_, toluene, reflux, 16 h. (**b**) AcCl, EtOH, 0°C, 7 h. (**c**) Glycine methyl ester hydrochloride, NEt_3_, CH_2_Cl_2_, r.t., 3 h. (**d**) EtOH, r.t., 16 h; or toluene, 120°C, 16 h. (**e**) MeI, DMF, r.t., 24 h. (**f**) R^5^CHO, cat. Sc(OTf)_3_, dioxane, 110°C, 24 h. (**g**) SeO_2_, dioxane, reflux, 2 h. (**h**) R^5^CH_2_PPh_3_Br, *n*BuLi, 0°C, 30 min; r.t., 16 h. (**i**) NH_2_OH·HCl, K_2_CO_3_, MeOH, r.t., 24 h.

The 36 derivatives of DMHBI investigated in this study were synthesized by an imine-imidate [3+2] cycloaddition strategy (34,35). Briefly, an aryl aldehyde (A) was first reacted with an alkyl or aryl amine to form the respective imine (B). The cycloaddition partner, an *N*-alkyl imidate (C), was generated from methyl glycinate and an ethyl imidate (D). Ethyl imidates were prepared from their parent nitriles (E) by reaction with ethanolic HCl in case they were not commercially available. Cycloaddition between the imines (B) and *N*-alkyl imidates (C) afforded the series of HBI chromophores of the general structure F (boxed in Scheme 1). A first series is represented by compounds **1**–**20** containing alkyl residues at C2 of the imidazolone ring (R^4^ = methyl or phenylethyl, Route A) and variable residues R^3^. In some cases with bulky R^3^ groups, the cycloaddition required extended reaction times and resulted in the competing formation of a side product **16** in which the imidate-N-methylglycinyl substituent instead of the imine-N substituent was incorporated into the HBI scaffold. The chromophores were isolated in pure form by chromatography and obtained as colored solids in medium to good yields. Selected examples with R^4^ = CH_3_, were oxidized with SeO_2_ to access the formylated intermediates G. In a second series, the π electron system was expanded to obtain chromophores **21**–**36** via one of three methods: 1) Sc(OTf)_3_-catalzyed aldol condensation of **1** or **9** with (hetero)aromatic aldehydes (Route B), 2) Wittig reaction of formylated HBI derivatives G with phosphorous ylides (Route C), or 3) condensation of the formylated HBI derivative obtained from **13** with hydroxylamine (Route D). The cationic HBI derivatives **14**, **34** and **36** were prepared by quaternization of the dimethylamino group of **13**, **33** and **35** with MeI as the final step of the reaction sequence of routes A, C and D.

For all DMHBI derivatives carrying an arylvinyl substituent at imidazolone-C2, ^1^H NMR showed that the vinyl group was exclusively in the *E* configuration with ^3^*J*_HH_ coupling constants of 15–16 Hz. Additionally, ^1^H-^1^H NOESY spectra show through-space correlations between the proximal vinyl proton and protons of the R^3^ residue, suggesting a predominant s-cis configuration of the exocyclic bond at C2 (see e.g. Supplementary Figure S1 for compound (**32**).

### Fluorescence screening

**Figure 1.**
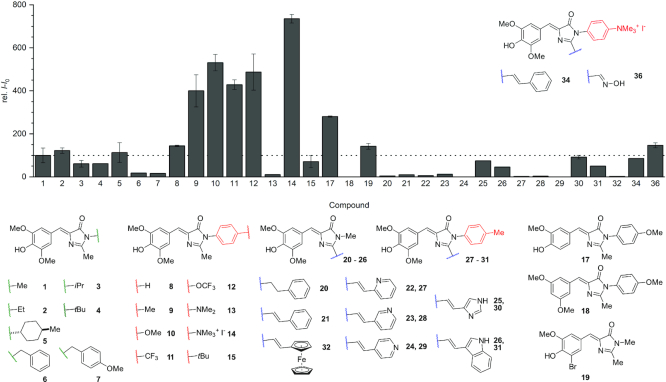
Fluorescence screening results for various HBI derivatives with the Chili aptamer (0.5 μm RNA, 0.5 μm dye, 125 mm KCl, 5 mm MgCl_2_, 80 mm HEPES pH 7.5). The blank-corrected intensity is given relative to the Chili-DMHBI **1** complex. Spectral data and intensity values are listed in Table 1 and spectra are shown in Supplementary Figures S2 and S3.

A simple screening protocol was used to examine the fluorescence turn-on of all the newly synthesized HBI derivatives by the Chili RNA aptamer. Briefly, ligands and pre-folded RNA were combined at a concentration of 0.5 μm each in a binding buffer containing 125 mm KCl and 5 mm MgCl_2_ at pH 7.5 and incubated at 25°C for 3 min; fluorescence emission spectra were collected by exciting the sample at the wavelength that elicited the maximum fluorescence response for the respective Chili RNA complex (Supplementary Figures S2 and S3). The fluorescence enhancement upon RNA binding for each chromophore is depicted in Figure 1, relative to the fluorescence of the Chili–DMHBI **1** complex, which was set to 100. The excitation and emission maxima as well as relative fluorescence values are summarized in Table 1. Several dyes showed strongly enhanced fluorescence, while others were only weakly activated upon binding to Chili. A low emission intensity may be caused by one or more of the following reasons: low affinity (under the experimental conditions, a *K*_D_ of 0.5 μmol l^−1^ or higher would result in less than 40% complex formation), slow uptake kinetics, slow RNA folding (restructuring of the binding site) or low quantum yield of the formed complex.

**Table 1.** Substitution patterns and spectral data for HBI derivatives **1**–**36**. Fluorescence intensity is given relative to the Chili-DMHBI 1 complex

Nr.	Name	R^1^	R^2^	R^3^	R^4^	R^5^	route	*λ*_Ex_/*λ*_Em_nm	Rel. Fl.Int.^[^^a^^]^
**1**	DMHBI	OMe	OH	Me	Me	-	A	400/537	100
**2**	DMHBI-Et	OMe	OH	Et	Me	-	A	400/537	122
**3**	DMHBI-*i*Pr	OMe	OH	*i*Pr	Me	-	A	400/537	61
**4**	DMHBI-*t*Bu	OMe	OH	*t*Bu	Me	-	A	400/534	61
**5**	DMHBI-MeCy	OMe	OH	4MeCy	Me	-	A	400/537	113
**6**	DMHBI-Bn	OMe	OH	PhCH_2_	Me	-	A	400/537	17.9
**7**	DMHBI-PMBn	OMe	OH	4MeOPhCH_2_	Me	-	A	400/535	16.3
**8**	DMHBPI	OMe	OH	Ph	Me	-	A	410/539	144
**9**	DMHBTI^[*]^	OMe	OH	4MePh	Me	-	A	410/539	400
**10**	DMHBAI^[#]^	OMe	OH	4MeOPh	Me	-	A	410/538	531
**11**	DMHBTI^F^	OMe	OH	4CF_3_Ph	Me	-	A	413/540	429
**12**	DMHBAI^F^	OMe	OH	4CF_3_OPh	Me	-	A	413/540	487
**13**	DMHBI-DMA	OMe	OH	4Me_2_NPh	Me	-	A	413/540	10.2
**14**	DMHBI^+^	OMe	OH	4Me_3_N^+^Ph	Me	-	A	413/542	735
**15**	DMHBI^C^	OMe	OH	4*t*BuPh	Me	-	A	410/539	71.0
**16**	DMHBI-spdt	OMe	OH	MeO(CO)CH_2_	Me	-	A	n.m.	n.m.
**17**	MHBAI	H	OH	4MeOPh	Me	-	A	397/513	280
**18**	DMBAI	OMe	H	4MeOPh	Me	-	A	n.d.	n.d.
**19**	BMHBI	Br	OH	Me	Me	-	^[b]^	386/520	142
**20**	DMHBI-PhEt	OMe	OH	Me	Ph(CH_2_)_2_	-	A	400/539	4.3
**21**	DMHBI-Styr	OMe	OH	Me	−	Ph	B	462/601	9.9
**22**	DMHBI-2Py	OMe	OH	Me	−	2Py	B^[c]^	467/616	5.9
**23**	DMHBI-3Py	OMe	OH	Me	−	3Py	B	465/611	12.3
**24**	DMHBI-4Py	OMe	OH	Me	−	4Py	B	n.d.	n.d.
**25**	DMHBI-Imi	OMe	OH	Me	−	4Imi	B	463/545,594	74.3
**26**	DMHBI-Ind	OMe	OH	Me	−	3Ind	B	469/539	45.6
**27**	DMHBTI-2Py	OMe	OH	4MePh	−	2Py	B	464/618	2.3
**28**	DMHBTI-3Py	OMe	OH	4MePh	−	3Py	B	467/613	3.2
**29**	DMHBTI-4Py	OMe	OH	4MePh	−	4Py	B	n.d.	n.d.
**30**	DMHBTI-Imi	OMe	OH	4MePh	−	4Imi	B	416,461/542,592^[d]^	92.0
**31**	DMHBTI-Ind	OMe	OH	4MePh	−	3Ind	B	478/539	50.1
**32**	DMHBI-Fc^[†]^	OMe	OH	Me	−	Fc	C	460/573	2.3
**33**	DMHBI-Styr-DMA	OMe	OH	4Me_2_NPh	−	Ph	C	n.m.	n.m.
**34**	DMHBI-Styr^+^	OMe	OH	4Me_3_N^+^Ph	−	Ph	C	465/603	85.2
**35**	DMHBO-DMA	OMe	OH	4Me_2_NPh	−	−	D	n.m.	n.m.
**36**	DMHBO^+^	OMe	OH	4Me_3_N^+^Ph	−	−	D	456/592	147

^a^Measurement conditions: 0.5 μm RNA, 0.5 μm dye, 125 mm KCl, 5 mm MgCl_2_, 80 mm HEPES pH 7.5, *λ*_Ex_ was set to the value indicated in the table. Samples without added RNA were used for the blank correction.

^b^Erlenmeyer azlactone synthesis.

^c^ZnCl_2_ was used as the catalyst instead of Sc(OTf)_3_ in the aldol condensation.

^d^DMHBTI-Imi shows a dual emission profile (Supplementary Figure S3j). n.d. no fluorescence detected. n.m. not measured. [*] T = *p*-tolyl. [#] A = *p*-anisyl. [†] Fc = ferrocenyl.

A first set of chromophores was used to examine the relationship between fluorescence activation and steric demand of the ligand side chain, irrespective of additional electrostatic and hydrogen bonding interactions. Therefore, the R^3^ substituent at the imidazolone-N3 of DMHBI (**1**) was varied from methyl to ethyl (**2**), isopropyl (**3**) and *tert*-butyl (**4**), respectively. A 40% loss of fluorescence intensity was observed when a linear substituent (compounds **1**, **2**) was replaced by a branched *N*-alkyl substituent (compounds **3**, **4**). Interestingly, compound **5** with the sterically even more demanding *trans*-4-methylcyclohexyl substituent showed comparable fluorescence intensity to complexes with **1** and **2**, suggesting that steric bulk is only a secondary factor. In contrast, the benzyl and *p*-methoxybenzyl substituents in compounds **6** and **7** resulted in complexes with 5-fold lower fluorescence intensity compared to the parent Chili–DMHBI complex. These effects are likely to be caused by an increased number of rotational degrees of freedom for larger substituents, which may increase the rates of internal conversion, thus resulting in lower fluorescence quantum yields (36).

**Figure 2.**
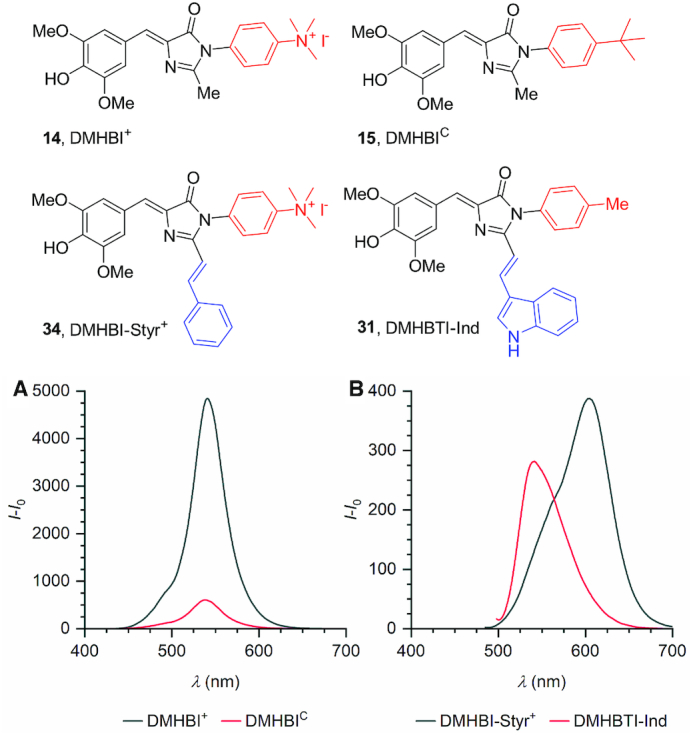
(**A**) The emission spectrum of Chili–DMHBI^+^ compared to Chili–DMHBI^C^. (**B**) Emission spectra of Chili–DMHBTI-Ind and Chili–DMHBI-Styr^+^. (0.5 μm RNA, 0.5 μm dye, 125 mm KCl, 5 mm MgCl_2_, 80 mm HEPES pH 7.5). Samples without added RNA were used for the blank correction.

A more direct interplay between the RNA and a bound ligand could arise from enhanced π–π stacking interactions. We therefore attached a phenyl group directly at the imidazolone-N3 without any alkyl linker resulting in compound **8**, and modulated the electron density of the phenyl group by means of the *para* substituent, resulting in a series of dyes **9–15**. Besides its strongly electron-withdrawing properties, the trifluoromethyl group was chosen because it is known to enhance the lipophilic character (37), which could have an impact on RNA binding of compounds **11** and **12**, in comparison to their non-fluorinated analogs **9** and **10**. Geometry optimization (DFT: B3LYP-D3/def2-TZVP) shows that these derivatives possess largely similar three-dimensional structures, i.e. the HBI core is planar and the N3-substituent is twisted out of plane by 50–59° (Supplementary Figure S4). One key difference is found in the calculated dipole moment of these chromophores, which is substantially larger for the heteroatom-containing derivatives **10–14** compared to **9** and **15**. Most significant is the large value of 16.7 D for DMHBI^+^ (**14**) due to its positively charged sidechain (Supplementary Table S1). In the fluorescence activation assay several interesting results were observed for this family of chromophores. First, we note that compound **9** (DMHBTI) in complex with Chili gave a 20-fold higher fluorescence intensity than its constitutional isomer **6** (DMHBI-Bn). Thus, the direct attachment of an aryl group to N3 proved beneficial in contrast to the alkylation with a benzyl group. While the Chili complex of DMHBPI (**8**) with R^3^ = phenyl showed similar fluorescence to Chili–DMHBI, a 4- to 5-fold enhanced fluorescence intensity was observed with ligands **9–12** that contained electron-donating methyl and methoxy groups, or electron-withdrawing CF_3_ and OCF_3_ substituents, respectively, at the para-position of R^3^. In stark contrast, a tenfold decreased fluorescence intensity compared to DMHBI was observed when this substituent was changed to a dimethyl-amino group in compound **13**. This quenching effect was expected, considering that the dimethylaniline (DMA) moiety is a known donor for intramolecular photoinduced electron transfer (PET) which leads to non-radiative decay of the excited state (38). *N*-methylation of **13** disrupts the PET and the resulting cationic ligand DMHBI^+^ (**14**) fluoresces more than seven times brighter than DMHBI when bound to the Chili aptamer. In other words, methylation of the DMA group enhances the fluorescence of the RNA-bound chromophore 73-fold. This strong fluorescence enhancement of **14** is a result of two factors: a higher affinity of Chili for DMHBI^+^ (leading to a more complete complex formation under the measurement conditions) and a higher fluorescence quantum yield of the resulting complex (34). To help understand how these two factors depend on the positive charge in the sidechain of **14**, a neutral isomorphic analog of DMHBI^+^ was synthesized: DMHBI^C^ (**15**) contained a *tert*-butyl group instead of the trimethylammonium substituent. The geometry-optimized structures of **14** and **15** are superimposable in the gas phase (Supplementary Figure S5). Surprisingly, however, the simple N-to-C substitution resulted in a tenfold loss of fluorescence enhancement under our screening conditions (Figure 2A). Although the Chili–DMHBI^C^ complex retained 70% of the intensity of the parent DMHBI, the distinct benefits of **14** compared to **15** warranted a more detailed analysis of the binding affinity and the influence of electrostatic effects (see below).

Next, we analyzed the spectral tuning of the N3-modified DMHBI chromophores and their complexes with Chili. Introduction of R^3^ = aryl groups led to a negligible shift of the free ligands’ **8–15** excitation and emission maxima (3 nm or less) compared to the alkyl derivatives **1–7** (Supplementary Table S2). However, when bound to the RNA, the difference in excitation maxima between the two groups of alkyl and aryl substituents consistently was in the range of 10–13 nm, whereas it remained unchanged for the emission maxima (Table 1). It follows that the aryl groups undergo a direct interaction with a specific part of the binding site irrespective of their electron density, which destabilizes the electronic ground state of the neutral ligand but not the excited state of its anion. A somewhat related effect was observed for the Spinach aptamer complex with DFHBI-1T, an imidazolone-N3-modified derivative of the parent ligand DFHBI (10). While the excitation maximum of the unbound ligand was only altered by 3 nm, its Spinach complex exhibited a more pronounced redshift of 35 nm. The emission maxima were shifted by 6 and 5 nm, respectively. Incidentally, the N3 substituent of DFHBI(-1T) is in close proximity to a specific nucleotide that was recently shown to govern the spectral tuning of Spinach-related aptamers (33). However, a significant difference between Spinach and Chili FLAPs remains the pronounced preference for Chili to bind exclusively the neutral phenol form of the chromophores (and thereby shifts the phenol – phenolate equilibria even at neutral pH), while Spinach can only bind and activate the anionic phenolate of DFHBI and its analogs.

Red-emitting HBI chromophores (i.e. *λ*_Em_ > 570 nm) differ from their green-emitting counterparts by an expanded π electron system at position C2 of the imidazolone ring. In nature, this is implemented in different classes of red fluorescent proteins through a small palette of functional groups such as acylimine (DsRed), 2-hydroxy-dihydrooxazolyl (mOrange) or vinylimidazolyl (Kaede) moieties, which are formed from specific tripeptides in the protein sequence during maturation (39). Their exact spectral characteristics are determined by a delicate interplay between the HBI chromophore and its surrounding protein environment (40–42). In designing new fluorogenic HBI ligands that interact with biomacromolecules, more tractable and chemically stable entities such as oximes (11,34) and simple benzenoid aromatics (43,44) have been favored so far.

DMHBI-Imi (**25**) and DMHBO^+^ (**36**) bind to Chili with good to excellent affinities, forming yellow to red-fluorescent complexes with emission maxima above 590 nm and quantum yields of 8% and 10%, respectively (34). In order to elucidate key structural features responsible for fluorophore binding and activation of C2-extended chromophores, we investigated several related derivatives. First, two model compounds **20** and **21** were synthesized in which the imidazolone-C2 methyl substituent of DMHBI was replaced by a saturated ethylphenyl (**20**) or an unsaturated vinylphenyl group (**21**), respectively. Compound **20** exhibited nearly the same excitation and emission maxima as DMHBI when bound to Chili, albeit with a strongly reduced fluorescence intensity (Supplementary Figure S2). In contrast, both the excitation and emission maxima of the Chili-bound derivative **21** were redshifted by 62 nm, at about twice the fluorescence intensity of the complex with **20**. Exchanging the imidazolone-N3 methyl substituent of **21** for the cationic trimethylammoniumphenyl sidechain in compound **34** led to a complete recovery of the fluorescence intensity with respect to DMHBI. This beneficial effect of a large dipole moment is consistent with the seven- to tenfold enhancement observed for the same positively charged side chain in DMHBI^+^ and DMHBO^+^. Therefore, DMHBI-Styr^+^ (**34**) represents a promising new ligand for Chili with an emission maximum beyond 600 nm (Figure 2B).

Next, additional hydrogen bond acceptor sites were introduced via R^5^, by replacing the vinylphenyl group in **21** with three different vinylpyridyl side chains. Compounds **22–24** were very weakly autofluorescent in aqueous buffer at pH 7.5, and binding to Chili resulted only in a minor increase in fluorescence of DMHBI-2Py (**22**) and DMHBI-3Py (**23**), reaching similar intensities as for compound **21** with emission maxima of 615 and 610 nm, respectively. However, no fluorescence enhancement was observed for DMHBI-4Py (**24**). DMHBI-4Py has recently been synthesized using a method similar to ours and was screened as a potential ligand for the fluorogenic protein tag FAST, but also in this case no increase in fluorescence intensity was detected (45).

In DMHBI-Imi **25**, which resembles the chromophore of the red fluorescent protein Kaede, both a hydrogen bond donor and acceptor site are present. When bound to Chili, it shows a remarkable, bimodal fluorescence spectrum upon excitation at 463 nm. Two components with emission maxima of 542 nm (38% integrated intensity) and 592 nm (62% integrated intensity) were extracted by fitting the experimental spectrum with a Gaussian function (Supplementary Figure S3o). Since the higher energy component closely resembles the emission profile of Chili–DMHBI, it can be attributed to the excited state of a fluorophore subpopulation that has undergone proton transfer from the imidazole moiety to a nearby acceptor and thereby lost its extended π conjugation. To further corroborate this hypothesis, we also synthesized DMHBI-Ind (**26**) containing a 3-indolyl substituent in place of the imidazolyl group. Indole itself is a known photoacid (46) and should therefore enable efficient NH proton transfer in Chili–DMHBI-Ind. Excitation of the complex at 469 nm resulted in an emission band with a prominent maximum at 537 nm and a broad shoulder on the red edge, which is consistent with the suggested mechanism (Supplementary Figure S3). Similar results were observed for a second series of chromophores, in which R^3^ was a 4-methylphenyl (i.e. *p*-tolyl) group to obtain the respective DMHBTI derivatives **27–31** (Figure 2B, Supplementary Figure S3). DMHBTI-Imi (**30**) was obtained as a 10:1 mixture of *E* and *Z* isomers at the newly formed double bond. Similar to DMHBI-Imi (**25**), its Chili complex shows two prominent emission bands at 542 and 592 nm. However, their relative intensity was dependent on the excitation wavelength (Supplementary Figure S3j), which is likely attributed the presence of electronically distinct geometric isomers. Finally, a ferrocenyl-expanded DMHBI analog **32** was screened. The redox-active organometallic side-chain could provide interesting properties for electrochemical sensor applications (47), but because only a very low level of fluorescence activation upon addition of Chili RNA was observed, this ligand was not investigated in further detail.

### Recognition of HBI by the Chili binding site

Molecular recognition of ligands in the RNA binding site is governed by specific supramolecular interactions. For the Spinach aptamer and its cognate DFHBI ligand, these interactions have been revealed by X-ray crystallography, and π stacking as well as direct and H_2_O-bridged hydrogen bonding have been identified as important interactions between DFHBI and the RNA aptamer. The ligand is situated between the top face of a G-quartet and an U–A–U base triple, where it acts as a hydrogen bond acceptor to a nearby adenine (via imidazolone-N1) and two guanines (via benzylidene-O4 and imidazolone-O4) (24). A similarly dense framework of interactions has been found in the crystal structure of Corn–DFHO, where the oxime moiety of the ligand additionally acts as an ambidentate hydrogen bond donor and acceptor at the same time (29).

**Figure 3.**
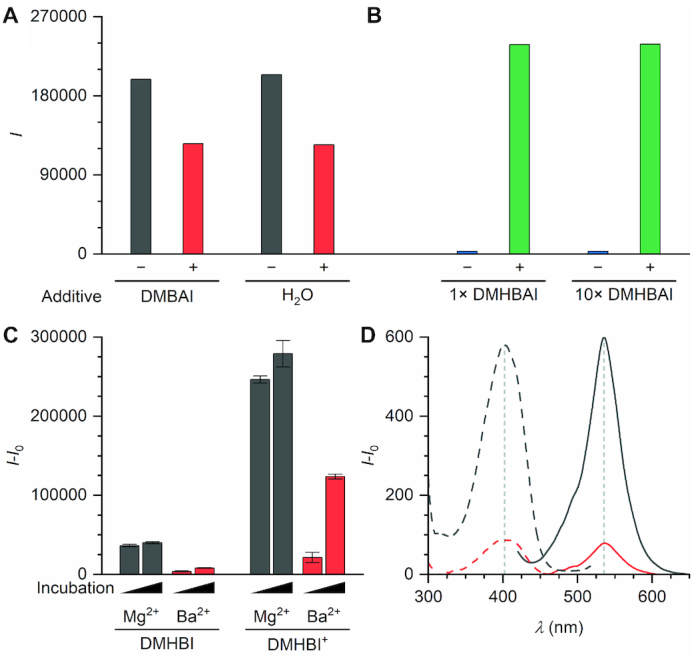
(**A**) Integrated fluorescence emission intensities for Chili–DMHBAI (0.5 μm RNA, 0.5 μm dye **10**, 125 mm KCl, 5 mm MgCl_2_, 40 mm HEPES pH 7.5) before and after adding a tenfold excess of DMBAI (**18**) or an equivalent volume of water. (**B**) Integrated fluorescence emission intensities for Chili–DMBAI (0.5 μm RNA, 5 μm dye, 125 mm KCl, 5 mm MgCl_2_, 40 mm HEPES pH 7.5) before and after adding 0.5 or 5 μm DMHBAI. (**C**) Integrated fluorescence emission intensities for Chili–DMHBI and Chili–DMHBI^+^ (0.5 μm RNA, 0.5 μm dye, 125 mm KCl, 5 mm MgCl_2_ or BaCl_2_, 80 mm HEPES pH 7.5) after 3 min and 24 h incubation time, respectively. (**D**) Fluorescence excitation (dashed) and emission (solid) spectra of Chili–DMHBI after 3 min incubation in the presence of MgCl_2_ (black) or BaCl_2_ (red).

In the absence of a crystal structure of the Chili RNA, the detailed mechanism of ligand recognition is unknown, but biochemical experiments can reveal essential functional groups for analogous supramolecular interactions. We showed previously that DFHBI is not strongly activated by the Chili aptamer (34), indicating a different ligand binding mode in Chili compared to Spinach. The characteristically large pseudo-Stokes shifts observed for Chili complexes show that ligand binding and fluorescence activation involves a proton transfer cycle (Scheme S1). Considering the high acidity of DFHBI compared to derivatives of DMHBI, the required protonation step to generate the binding-competent phenol form is likely impeded in this case. Additionally, because of significant differences in size, electronegativity, and H-bonding potential, the fluorine substituents may be mismatched to the environment that has been selected to accomodate methoxy substituents. To address the possibilities of specific recognition of the phenolic OH and the neighboring OMe groups, two analogs of DMHBAI (**10**) were examined, in which either one of the methoxy groups (MHBAI, **17**) or the phenolic hydroxy group (DMBAI, **18**) were absent. The fluorescence emission of Chili–MHBAI (**17**) was half as intense as that of Chili–DMHBAI (**10**), indicating that both OMe groups contribute to the optimal occupancy of the binding site. Interestingly, one OMe group of the original DMHBI ligand could be replaced by bromine, resulting in the ligand BMHBI (**19**), which gave rise to a chromophore–RNA complex of comparable intensity. In stark contrast, however, no fluorescence emission was detected when DMBAI (**18**) was combined with Chili under standard screening conditions. This result highlights the importance of the phenolic OH group. A competition experiment between DMBAI (**18**) and DMHBAI (**10**) was then performed to distinguish between non-binding and non-activation. Briefly, one equivalent of the preformed Chili–DMHBAI complex was treated with either buffer or a tenfold excess of DMBAI in buffer. Since the observed decrease in fluorescence emission was identical in both cases, DMBAI could not have displaced DMHBAI from the binding site (Figure 3A). In the reverse direction, one or ten equivalents of DMHBAI were added to a solution of Chili containing ten equivalents of DMBAI. The identical increase in fluorescence intensity shows that DMBAI could not have occupied the binding site of Chili (Figure 3B). These results indicate that the binding site of Chili contains a hydrogen bond acceptor that not only facilitates proton abstraction from the photoexcited ligand but that is also crucial for ligand binding in the ground state. Secondary interactions with the methoxy groups (or isosteric replacements) enhance the fluorescence by constraining the bound ligand into a specific conformation.

We also tested whether ligand recognition was influenced by the presence or absence of specific divalent metal ions. Chili requires up to 5 mm Mg^2+^ to effectively turn on the fluorescence of DMHBI, while strongly enhanced DMHBI^+^ fluorescence was observed regardless of the Mg^2+^ concentration (34). When MgCl_2_ in the binding buffer was replaced by BaCl_2_, the fluorescence intensity for either ligand dropped by a factor of approx. 10 (Figure 3C). It recovered over the course of 24 h and remained stable at a moderate level for several days. In contrast, in the presence of Mg^2+^, high fluorescence intensity was observed immediately, which increased by <10% upon prolonged incubation (Figure 3C). Unlike Spinach–DFHBI, whose emission maximum is strongly shifted depending on the nature of the divalent metal ion (24), neither of the two Chili complexes displayed any form of spectral tuning upon cation replacement (Figure 3D). This suggests that Chili complexes lack specific cation–ligand interactions; instead, only the global stability of the active RNA conformation is influenced due to differences in the coordination environment of the respective metal ion.

### Binding affinity

While association with the RNA aptamer is a requirement for fluorescence turn-on of the ligand, affinity reflects interactions in the electronic ground state of the complex, whereas fluorogenic enhancement is an excited state property. Both aspects are therefore subject to different, not necessarily correlated, mechanisms. To examine the effects of ligand substituents on binding affinities, the dissociation constants of Chili complexes with HBI derivatives **9–12** (selected based on the strong fluorescence turn-on during the screening) were measured using fluorescence titration experiments, in which the ligand concentration was held constant and the RNA concentration was increased up to 40 μM. Both DMHBTI (**9**) and DMHBAI (**10**) are strong binders with a *K*_D_ of 151 ± 21 and 65 ± 7 nm, respectively. In contrast, saturation binding was not achieved for DMHBTI^F^ (**11**) and DMHBAI^F^ (**12**); their dissociation constants are estimated to be greater than 1 μm. DMHBI^C^ (15), the neutral analog of DMHBI^+^, was also found to be a weak binder with an estimated *K*_D_ of >0.7 μm (Supplementary Figure S6).

**Figure 4.**
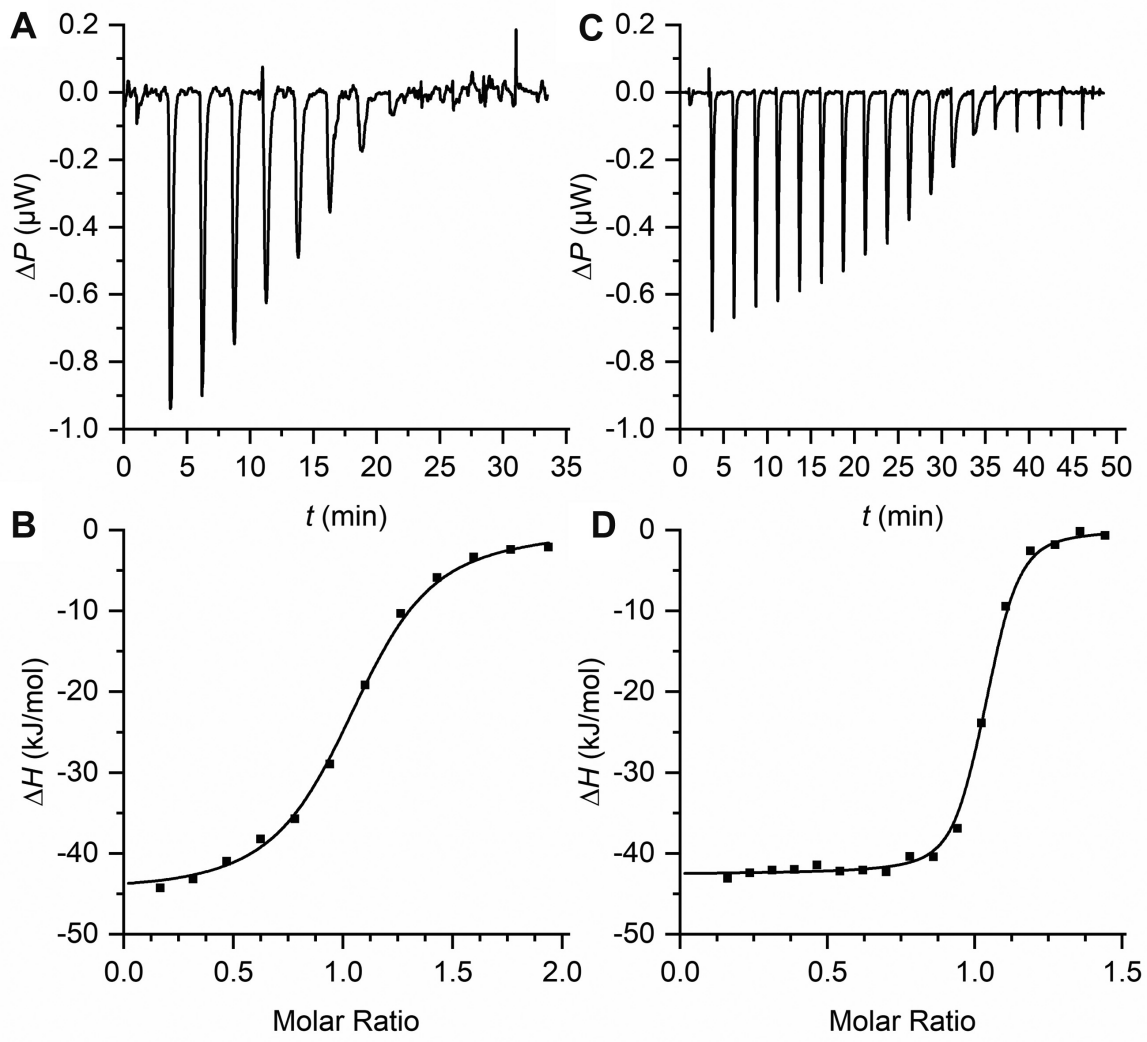
ITC data for the formation of Chili–DMHBI (**A**, **B**) and Chili–DMHBI^+^ (**C**, **D**) at 25°C. Background-corrected differential power and offset-corrected integrated heat are shown for a single representative run of three. The data was fitted with a one-site-binding model.

In addition, the binding affinities of DMHBI (**1**) and DMHBI^+^ (**14**) were assessed by isothermal titration calorimetry (ITC) to determine the respective enthalpic and entropic contributions of the binding process (Figure 4, Table 2). For both, the calorimetric dissociation constants (DMHBI: 371 ± 55 nm; DMHBI^+^: 24.1 ± 5.6 nm) are in good agreement with those obtained by fluorescence titration (34). While no substantial difference in binding enthalpy was observed (−44 and −42 kJ mol^−1^, respectively), the binding entropy is unfavorable for DMHBI (−276 J mol^−1^ K^−1^), but slightly favorable for DMHBI^+^ (78 J mol^−1^ K^−1^) which results in an overall more negative Δ*G* at 25°C for the latter.

**Table 2.** Thermodynamic data for the formation of Chili complexes from ITC experiments

Ligand	Δ*H*, kJ mol^−1^	Δ*S*, J mol^−1^ K^−1^	Δ*G*^a^, kJ mol^−1^	*K*_D_, nmol L^−1^	*K*_D_ ^b^, nmol L^−1^
DMHBI	−44	−276	−37	371	570
DMHBI^+^	−42	+78	−44	24.1	63

^a^Δ*G* = ΔH− *T* Δ*S* at 298 K.

^b^Determined by fluorescence titration (34).

In general, the entropy change of binding encompasses a number of different factors such as the reduction of the number of unbound molecules in solution, loss of conformational freedom in the complex, and the release of water molecules due to a loss of solvent-accessible surface area (48). A positive Δ*S* is characteristic for such a desolvation effect and reflects the increased hydration of the cationic side chain in DMHBI^+^ compared to DMHBI. This further suggests that the lower affinity of HBI derivatives **11**, **12** and **15** is the result of an entropic effect caused by the hydrophobic nature of their aromatic side chains. However, once a complex with the Chili aptamer is formed, a higher degree of fluorescence turn-on compared to N3-alkylated derivatives is achieved.

### Binding kinetics

**Figure 5.**
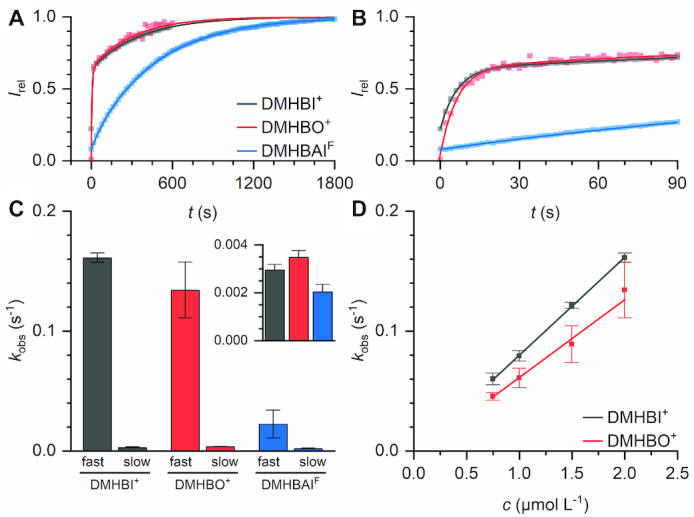
(**A**) Fluorescence activation kinetics of Chili with DMHBI^+^, DMHBO^+^ and DMHBAI^F^ under pseudo-first order conditions (0.025 μm RNA, 2 μm dye, 125 mm KCl, 5 mm MgCl_2_, 40 mm HEPES pH 7.5). Data points were collected in 2 s intervals, every 10th point is plotted. Solid lines represent biexponential fits. (**B**) First 90 s of the data in panel a without decimation. (**C**) *k*_obs_ values for the biexponential fits in panel A). The inset shows only the slow *k*_obs_ values. (**D**) Plot of *k*_obs_ against the dye concentration (0.025 μm RNA, dye as indicated, 125 mm KCl, 5 mmM MgCl_2_, 40 mm HEPES pH 7.5). Solid lines represent linear fits to obtain *k*_on_.

In contrast to other fluorogenic RNA systems like Spinach–DFHBI, Chili was shown to exhibit biexponential kinetics when activating the fluorescence of DMHBO^+^ **36**, which was attributed to a combination of ligand uptake and refolding of the Chili aptamer (34,49,50). We therefore examined the activation kinetics of two additional ligands with different affinities, DMHBI^+^ (**14**) and DMHBAI^F^ (**12**) to test whether this behavior is a general property of the Chili RNA. Under pseudo-first order conditions, the fluorescence signal was found to rise biexponentially for both dyes (Figure 5A, B, Supplementary Figure S7). However, while the apparent rate constants (*k*_obs_) for DMHBI^+^ and DMHBO^+^ were not significantly different, DMHBAI^F^ was activated at a more than sixfold slower rate (Figure 5C).

Bimolecular association rates (*k*_on_) were obtained from determining *k*_obs_ over a range of different ligand concentrations; the corresponding dissociation rates (*k*_off_) were then estimated from the relation *k*_off_ = *k*_on_ · *K*_D_ (Table 3). These results show that DMHBI^+^ and DMHBO^+^ bind to Chili with similar rates on the order of 10^4^ l mol^−1^ s^−1^ (Figure 5D), which is typical for the complex formation between aptamers or riboswitches and their cognate ligands (51). The lower dissociation constant of Chili–DMHBO^+^ therefore reflects a slower unbinding of this fluorophore compared to DMHBI^+^, which may be explained among other reasons by the larger number of molecular interactions due to the extended oxime functional group.

**Table 3.** Rate constants for the association and dissociation of Chili complexes from kinetic experiments according to Figure 5D

Ligand	*K*_D_ ^a^, nmolL^−1^	*k*_on_, L mol^−1^ s^−1^	*k*_off_ ^b^, s^−1^
DMHBI^+^	63	8.2 × 10^4^	5.1 × 10^−3^
DMHBO^+^	12	6.5 × 10^4^	7.8 × 10^−4^

^a^Dissociation constants from (34).

^b^ *k*_off_ = *k*_on_ · *K*_D_.

### UV–Vis and fluorescence melting analysis

**Figure 6.**
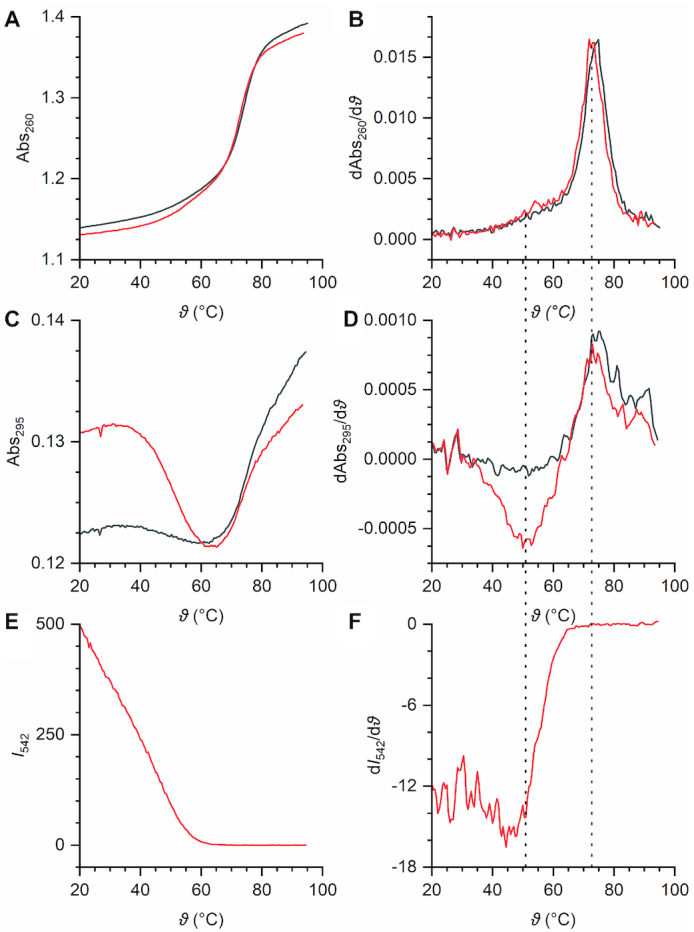
Thermal melting profiles of Chili (black) and Chili–DMHBI^+^ (red) (2 μm RNA, 2 μm dye, 125 mm KCl, 40 mm HEPES pH 7.5, temperature ramp 0.5°C/min). (**A**) Absorbance and (**B**) first derivative of the absorbance at 260 nm. (**C**) Absorbance and (**D**) first derivative of the absorbance at 295 nm. (**E**) Fluorescence emission and (**F**) first derivative of the fluorescence emission at 413/542 nm (Ex/Em). Curves are shown for the second of four reversible ramps.

To study folding and unfolding of the Chili RNA and its fluorescent complex with HBI ligands, temperature-dependent changes in the absorbance of Chili and Chili–DMHBI^+^ were monitored at different wavelengths between 10 and 95°C. At 260 nm, where melting of canonical duplex structures is dominant, both systems showed strong hyperchromicity with similar transitions at 73°C, which reflect the dissociation of the bottom stem and top stem loop of Chili, respectively (Figure 6A, B). Additionally, a strongly hypochromic transition at 51°C was observed for Chili–DMHBI^+^ at 295 nm, but not for Chili in the absence of DMHBI^+^. This observation reflects the ligand-induced formation of a G-quadruplex motif (Figure 6C, D) (52). At the same time, Chili–DMHBI^+^ exhibited a linear decrease in fluorescence emission at 542 nm; no fluorescence was detected above 63°C (Figure 6E). This decline correlates with the G-quadruplex unfolding, which further emphasizes the interdependence of ligand binding, quadruplex formation and fluorescence activation for Chili (Figure 6F). Parallels can be drawn to the thermal properties of the Spinach and Mango aptamers: Spinach does not gain stabilization from ligand binding because, like in the present case, dissociation of the complex precedes the melting of Watson–Crick secondary structure motifs. Mango, on the other hand, which is essentially a minimal G-quadruplex domain, is strongly stabilized by ligand binding, i.e. melting and fluorescence loss occur simultaneously (31,19).

### Mutagenesis of the Chili aptamer

**Figure 7.**
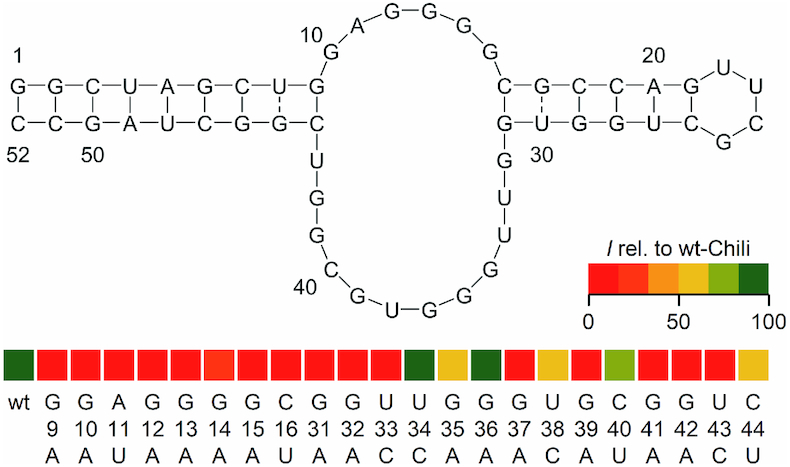
Secondary structure of Chili and mutagenesis pattern of the binding site. Color indicates fluorescence enhancement of DMHBI **1** relative to wt-Chili (0.5 μm RNA, 0.5 μm dye, 125 mm KCl, 5 mm MgCl_2_, 80 mm HEPES pH 7.5). See data in Supplementary Table S3.

Chili was designed as a folding-optimized variant of the 13–2 aptamer by modifying the stem loop regions adjacent to the central bulge, which itself was left unaltered (34). We prepared single-nucleotide transition mutants for all nucleotides of the central region (except A11, which was mutated to U, in order to avoid seven consecutive Gs) and compared their ability to activate DMHBI to that of the parent Chili RNA. The majority of nucleotides could not be mutated without rendering the aptamer virtually inactive, which is reflected by the red colored boxes in Figure 7. Mutants U34C, G35A, G36A and U38C retained their activity partially or even fully, which is consistent with previously reported structural probing data that demonstrated enhanced susceptibility to hydrolysis of this region in the parent Chili RNA (Figure 7) (34).

Based on these findings, several mutants were chosen for a more detailed analysis, including a comparison between activation of DMHBI and DMHBI^+^. G35A and G36A, representing the only positions in the binding site where mutation of a G was tolerated, were found to activate DMHBI to the same degree as the parent Chili; DMHBI^+^ fluorescence, however, was weaker, especially with G35A. This difference lessened after overnight incubation, suggesting a slower reorganization of the mutant binding site. G9 and C44 are predicted to form a base pair at the junction between the binding site and the bottom stem of Chili. Both G9A and the double mutant G9A/C44U are completely inactive. The mutation C44U, which introduces a G–U wobble base pair, was tolerated, but resulted in a significant loss of fluorescence enhancement to about one third of the parent intensity (Supplementary Figure S8). The base pair at the junction between the binding site and the top stem loop is likely formed by C16 and G31. Here, a C–G Watson–Crick base pair is strictly required for HBI activation, since both single point mutants as well as the C16U/G31A double mutant with a restored U–A base pair remained unable to bind DMHBI and DMHBI^+^ (Supplementary Figure S8). Although these data are not sufficient to unambiguously identify the guanines involved in formation of the quadruplex core structure, the formation of functional G-quartets is further corroborated by the activation of two quadruplex-binding fluorogenic dyes, thiazole orange (TO) and thioflavin T (ThT) by wt-Chili and a number of transition mutants (Supplementary Figure S9), as well as by NMR spectroscopy.

### NMR spectroscopy of the Chili aptamer

**Figure 8.**
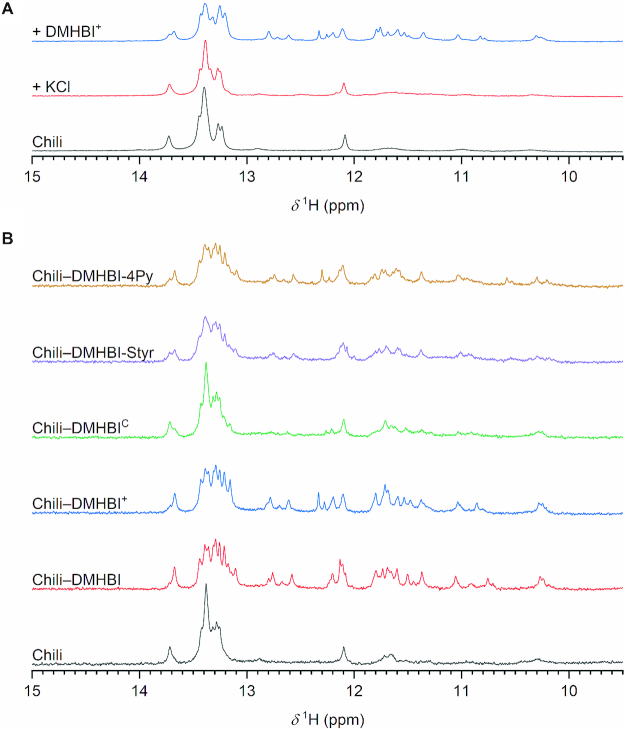
(**A**) Imino ^1^H NMR spectra of Chili before and after addition of KCl followed by DMHBI^+^ (150 μm RNA, 1 mm MgCl_2_, 25 mm TRIS pH 7.4, 10% D_2_O/90% H_2_O; + 50 mm KCl; + 150 μm dye). (**B**) Imino ^1^H NMR spectra of Chili alone and in the presence of various ligands (20 μm RNA, 20 μm dye, 50 mm KCl, 1 mm MgCl_2_, 25 mm KP_i_ pH 7.4, 10% D_2_O/90% H_2_O).

The imino region of the ^1^H NMR spectra of RNA provides insightful information on the secondary structure because the chemical shift of imino protons involved in hydrogen bonding is highly sensitive to the nature of the hydrogen bond: imino protons engaged in canonical Watson-Crick base pairs resonate in the region of 12–15 ppm, while imino protons involved in G–U wobbles or in the formation of G-quartets via Hoogsteen base pairing typically resonate between 10 and 12 ppm (53,54). The ^1^H NMR spectrum of Chili in the apo state shows signals in the imino region (Figure 8A), which likely arise from partial hybridization of the top and bottom stems, as revealed by comparison with the spectra of synthetic stem loops that comprise the nucleotides 1–9/44–52 and 16–31 of the Chili RNA (Supplementary Figure S10). The imino pattern did not change noticeably upon sequential addition of Mg^2+^ and K^+^, suggesting that metal ions are not competent to induce folding of the aptamer core. Only after addition of DMHBI^+^, a new set of well-dispersed imino signals appeared, indicating the ligand-induced formation of a binding pocket (Figure 8A). The chemical shift of the new signals suggests the formation of canonical Watson-Crick hydrogen bonds as well as non-canonical base pairs (e.g. Hoogsteen-type). Interestingly, after transferring the sample into D_2_O a set of signals in the region 10.7–11.8 ppm as well as two signals at 12.2 and 12.8 ppm, respectively, were still observed (Supplementary Figure S11). In particular, the signals around 11 ppm are still detectable at room temperature for several hours, suggesting that these protons are highly protected from exchange with water and pointing at the formation of a 2-tetrad G-quadruplex, as previously proposed for the 13–2 aptamer in presence of the non-canonical ligand DFHBI (24). The formation of a G-quadruplex structure is also supported by the K^+^ dependence of the fluorescence emission (34) and the thermal melting experiments discussed above. This process is apparently decoupled from the slow kinetic component of fluorescence activation (discussed in Figure 5), as no time-dependent changes were observed in the imino region of the NMR spectrum (Supplementary Figure S12).

Under similar experimental conditions, DMHBI gave only slightly different NMR results to DMHBI^+^ (Figure 8B). In case of DMHBI^C^, despite 80% of the RNA being bound to the ligand (estimated according to Supplementary Figure S6E) the imino resonances broadened, suggesting that DMHBI^C^ binds to the Chili RNA in the intermediate exchange regime (Figure 8B). In fact, the residence time of the ligand at the binding site is short compared to that of DMHBI^+^ (∼200 s, calculated as 1/*k*_off_, see Table 3). Because DMHBI-Styr (**21**) and its aza analog DMHBI-4Py (**24**) were found to be weakly or non-fluorogenic, respectively, they were also tested for binding by ^1^H NMR. Both ligands gave rise to an imino resonance pattern comparable to that observed for DMHBI^+^ and DMHBI in terms of number of signals and resonance dispersion, but with a larger degree of line broadening. Therefore, the ^1^H NMR spectra suggest the formation of a ligand-induced G-quadruplex structure in the binding pocket also in the presence of DMHBI-4Py and DMHBI-Styr. The low fluorescence of their Chili complexes does not primarily result from weak binding to the G-quadruplex, but instead from the lack of additional structural motifs able to modulate their fluorescence profile, such as nucleotides stacking onto their aromatic moieties. Therefore, the induction of a G-quadruplex structure is necessary but not sufficient for bright fluorescence activation. Additional capping structures must play an important role in defining the fluorogenic behavior of each dye. The imino protons detected in the NMR spectrum report only on the formation of hydrogen bonds, but do not give any information on, e.g., a nucleotide that is stacking but not base-paired. The slow rearrangement of such a capping structure could be also the reason why a slow kinetic component of fluorescence activation was detected, but no corresponding time-dependent changes in the NMR imino region were observed.

## CONCLUSIONS

The fluorogenic RNA aptamer Chili was investigated for its ability to activate the fluorescence of thirty-six differently substituted HBI chromophores. The dye structures were inspired by chromophores found in fluorescent proteins, and were synthetically obtained using a cycloaddition-based strategy, which in most cases gave more reliable results than the Erlenmeyer azlactone route. While alkyl substituents were less favored at N3 of the imidazolone ring, a set of strongly fluorogenic dyes was obtained that contained aryl groups at this position. These chromophores showed overall bright fluorescence emission, but the binding affinities varied between less than 100 nm and above 1 μm. The tightest binding was observed for ligands with cationic trimethylammoniumphenyl side chains. Combined with the expansion of the π system, this strategy resulted in new red-emitting RNA complexes with emission maxima beyond 600 nm. A large dipole moment of the chromophore was connected to strong fluorescence enhancement, and importantly, the specificity for the Chili aptamer was not impaired by enhanced electrostatic attraction. The thermodynamics of ligand binding was investigated by ITC, and the approximately tenfold stronger binding of the cationic DMHBI^+^ was identified as an entropic effect. The differences in affinity compared to uncharged analogs were also mirrored in the fluorescence activation kinetics, which showed a slower fluorescence intensity increase for lower affinity ligands. Two factors were found to contribute to fluorescence activation: Fast formation of the aptamer-ligand complex, followed by a slow structural reorganization of the binding site. ^1^H NMR spectroscopy showed that the binding site of Chili is not preorganized in the apo state, cannot be induced by metal ions alone, but readily adopts its folded structure only upon ligand binding. The newly formed imino proton signals as well as the results of temperature-dependent UV spectroscopy are characteristic for the folding of a G-quadruplex. Future NMR studies will employ site-specific isotope labeling to identify individual nucleobases interacting with the ligands. Overall, our study helps to understand the unique features of the Chili aptamer on a structural basis to guide further optimization and application of the aptamer in combination with suitable ligands as a bioanalytical tool.

## Supplementary Material

gkz1084_Supplemental_File
