# Supplementary material for: Structure–fluorescence activation relationships of a large Stokes shift fluorogenic RNA aptamer
Source: Nucleic Acids Res. 2019 Nov 19;47(22):11538–50. doi: 10.1093/nar/gkz1084 (PMC7145527; doi:10.1093/nar/gkz1084)
Supplement: gkz1084_Supplemental_File [file gkz1084_supplemental_file.pdf]

Table of contents

1 Experimental Procedures 4
1.1 General Information 4
1.1.1 Materials 4
1.1.2 NMR spectroscopy 4
1.1.3 Mass spectrometry 4
1.2 Synthetic procedures for chromophores 4
1.2.1 General procedure A, imine synthesis with volatile amines 4
1.2.2 General procedure B, imine synthesis with non-volatile amines 4
1.2.3 General procedure C, cycloaddition reaction 4
1.2.4 General procedure D, Aldol condensation 4
1.2.5 4-Hydroxy-3,5-dimethoxy-*N*-ethylbenzaldimine (**S1**) 4
1.2.6 4-Hydroxy-3,5-dimethoxy-*N*-isopropylbenzaldimine (**S2**) 5
1.2.7 4-Hydroxy-3,5-dimethoxy-*N*-(*tert*-butyl)benzaldimine (**S3**) 5
1.2.8 4-Hydroxy-3,5-dimethoxy-*N*-(*trans*-4-methylcyclohexyl)benzaldimine (**S4**) 5
1.2.9 4-Hydroxy-3,5-dimethoxy-*N*-benzylbenzaldimine (**S5**) 5
1.2.10 4-Hydroxy-3,5-dimethoxy-*N*-(4-methoxybenzyl)benzaldimine (**S6**) 6
1.2.11 4-Hydroxy-3,5-dimethoxy-*N*-phenylbenzaldimine (**S7**) 6
1.2.12 4-Hydroxy-3,5-dimethoxy-*N*-(4-methylphenyl)benzaldimine (**S8**) 6
1.2.13 4-Hydroxy-3,5-dimethoxy-*N*-(4-methoxyphenyl)benzaldimine (**S9**) 7
1.2.14 4-Hydroxy-3,5-dimethoxy-*N*-(4-trifluoromethylphenyl)benzaldimine (**S10**) 7
1.2.15 4-Hydroxy-3,5-dimethoxy-*N*-(4-trifluoromethoxyphenyl)benzaldimine (**S11**) 7
1.2.16 4-Hydroxy-3,5-dimethoxy-*N*-(4-(*tert*-butyl)phenyl)benzaldimine (**S12**) 7
1.2.17 4-Hydroxy-3-methoxy-*N*-(4-trifluoromethoxyphenyl)benzaldimine (**S13**) 8
1.2.18 3,5-dimethoxy-*N*-(4-methoxyphenyl)benzaldimine (**S14**) 8
1.2.19 Ethyl 3-phenylpropionimidate hydrochloride (**S15**) 8
1.2.20 Methyl (*Z*)-2-((1-ethoxyethylidene)amino)acetate (**S16**) 9
1.2.21 Methyl (*Z*)-2-((1-ethoxy-3-phenylpropylidene)amino)acetate (**S17**) 9
1.2.22 (*Z*)-4-(4-Acetyloxy-3-bromo-5-methoxybenzylidene)-2-methyl-5(4*H*)-oxazolone (**S18**) 9
1.2.23 (*Z*)-4-(4-Hydroxy-3,5-dimethoxybenzylidene)-1-methyl-5-oxo-4,5-dihydro-1*H*-imidazole-2-carbaldehyde (**S19**) 10
1.2.24 (Hydroxymethyl)ferrocene (**S20**) 10
1.2.25 Triphenylphosphonium bromide (**S21**) 10
1.2.26 (Ferrocenylmethyl)triphenylphosphonium bromide (**S22**) 10
1.2.27 Benzyltriphenylphosphonium bromide (**S23**) 11
1.2.28 (*Z*)-3-Ethyl-5-(4-hydroxy-3,5-dimethoxybenzylidene)-2-methyl-3,5-dihydro-4*H*-imidazol-4-one (DMHBI-Et, **2**) 11
1.2.29 (*Z*)-5-(4-Hydroxy-3,5-dimethoxybenzylidene)-3-isopropyl-2-methyl-3,5-dihydro-4*H*-imidazol-4-one (DMHBI-*i*Pr, **3**) 11
1.2.30 (*Z*)-3-(*tert*-Butyl)-5-(4-hydroxy-3,5-dimethoxybenzylidene)-2-methyl-3,5-dihydro-4*H*-imidazol-4-one (DMHBTI-*t*Bu, **4**) 12
1.2.31 (*Z*)-5-(4-Hydroxy-3,5-dimethoxybenzylidene)-2-methyl-3-(*trans*-4-methylcyclohexyl)-3,5-dihydro-4*H*-imidazol-4-one (DMHBI-MeCy, **5**) 12
1.2.32 (*Z*)-3-Benzyl-5-(4-hydroxy-3,5-dimethoxybenzylidene)-2-methyl-3,5-dihydro-4*H*-imidazol-4-one (DMHBI-Bn, **6**) 12
1.2.33 (*Z*)-5-(4-Hydroxy-3,5-dimethoxybenzylidene)-3-(4-methoxybenzyl)-2-methyl-3,5-dihydro-4*H*-imidazol-4-one (DMHBI-PMBn, **7**) 13
1.2.34 (*Z*)-5-(4-Hydroxy-3,5-dimethoxybenzylidene)-2-methyl-3-phenyl-3,5-dihydro-4*H*-imidazol-4-one (DMHBPI, **8**) 13
1.2.35 (*Z*)-5-(4-Hydroxy-3,5-dimethoxybenzylidene)-2-methyl-3-(4-methylphenyl)-3,5-dihydro-4*H*-imidazol-4-one (DMHBTI, **9**) 13

1.2.36 (*Z*)-5-(4-Hydroxy-3,5-dimethoxybenzylidene)-3-(4-methoxyphenyl)-2-methyl-3,5-dihydro-4*H*-imidazol-4-one (DMHBAI, **10**) ... 14

1.2.37 (*Z*)-5-(4-Hydroxy-3,5-dimethoxybenzylidene)-3-(4-trifluoromethylphenyl)-2-methyl-3,5-dihydro-4*H*-imidazol-4-one (DMHBTI^F, **11**) ... 14

1.2.38 (*Z*)-5-(4-Hydroxy-3,5-dimethoxybenzylidene)-3-(4-trifluoromethoxyphenyl)-2-methyl-3,5-dihydro-4*H*-imidazol-4-one (DMHBAI^F, **12**) ... 14

1.2.39 (*Z*)-3-(4-*tert*-Butylphenyl)-5-(4-hydroxy-3,5-dimethoxybenzylidene)-2-methyl-3,5-dihydro-4*H*-imidazol-4-one (DMHBI^C, **15**) ... 15

1.2.40 Methyl (*Z*)-4-(4-Hydroxy-3,5-dimethoxybenzylidene)-2-methyl-5-oxo-4,5-dihydro-1*H*-imidazol-1-yl)acetate (DMHBI-spdt, **16**) 15

1.2.41 (*Z*)-5-(4-Hydroxy-3-methoxybenzylidene)-2-methyl-3-(4-trifluoromethoxyphenyl)-3,5-dihydro-4*H*-imidazol-4-one (MHBAI, **17**) ... 16

1.2.42 (*Z*)-5-(3,5-Dimethoxybenzylidene)-3-(4-methoxyphenyl)-2-methyl-3,5-dihydro-4*H*-imidazol-4-one (DMBAI, **18**) ... 16

1.2.43 (*Z*)-5-(3-Bromo-4-hydroxy-5-methoxybenzylidene)-2,3-dimethyl-3,5-dihydro-4*H*-imidazol-4-one (BMHBI, **19**) ... 16

1.2.44 (*Z*)-5-(4-Hydroxy-3,5-dimethoxybenzylidene)-3-methyl-2-phenylethyl-3,5-dihydro-4*H*-imidazol-4-one (DMHBI-PhEt, **20**) 17

1.2.45 5-((*Z*)-4-Hydroxy-3,5-dimethoxybenzylidene)-3-methyl-2-((*E*)-2-phenylvinyl)-3,5-dihydro-4*H*-imidazol-4-one (DMHBI-Styr, **21**) 17

1.2.46 5-((*Z*)-4-Hydroxy-3,5-dimethoxybenzylidene)-3-methyl-2-((*E*)-2-(pyridin-2-yl)vinyl)-3,5-dihydro-4*H*-imidazol-4-one (DMHBI-2Py, **22**) ... 17

1.2.47 5-((*Z*)-4-Hydroxy-3,5-dimethoxybenzylidene)-3-methyl-2-((*E*)-2-(pyridin-3-yl)vinyl)-3,5-dihydro-4*H*-imidazol-4-one (DMHBI-3Py, **23**) ... 18

1.2.48 5-((*Z*)-4-Hydroxy-3,5-dimethoxybenzylidene)-3-methyl-2-((*E*)-2-(pyridin-4-yl)vinyl)-3,5-dihydro-4*H*-imidazol-4-one (DMHBI-4Py, **24**) ... 18

1.2.49 2-((*E*)-2-(1*H*-Indol-3-yl)vinyl)-5-((*Z*)-4-hydroxy-3,5-dimethoxybenzylidene)-3-methyl-3,5-dihydro-4*H*-imidazol-4-one (DMHBI-Ind, **26**) ... 18

1.2.50 5-((*Z*)-4-Hydroxy-3,5-dimethoxybenzylidene)-3-(4-methylphenyl)-2-((*E*)-2-(pyridin-2-yl)vinyl)-3,5-dihydro-4*H*-imidazol-4-one (DMHBTI-2Py, **27**) ... 19

1.2.51 5-((*Z*)-4-Hydroxy-3,5-dimethoxybenzylidene)-3-(4-methylphenyl)-2-((*E*)-2-(pyridin-3-yl)vinyl)-3,5-dihydro-4*H*-imidazol-4-one (DMHBTI-3Py, **28**) ... 19

1.2.52 5-((*Z*)-4-Hydroxy-3,5-dimethoxybenzylidene)-3-(4-methylphenyl)-2-((*E*)-2-(pyridin-4-yl)vinyl)-3,5-dihydro-4*H*-imidazol-4-one (DMHBTI-4Py, **30**) ... 20

1.2.53 2-(2-(1*H*-Imidazol-4-yl)vinyl)-5-((*Z*)-4-hydroxy-3,5-dimethoxybenzylidene)-3-(4-methylphenyl)-3,5-dihydro-4*H*-imidazol-4-one (DMHBTI-Imi, **30**) ... 20

1.2.54 2-((*E*)-2-(1*H*-Indol-3-yl)vinyl)-5-((*Z*)-4-hydroxy-3,5-dimethoxybenzylidene)-3-(4-methylphenyl)-3,5-dihydro-4*H*-imidazol-4-one (DMHBTI-Ind, **31**) ... 20

1.2.55 2-((*E*)-2-(Ferrocenyl)vinyl)-5-((*Z*)-4-hydroxy-3,5-dimethoxybenzylidene)-3-(4-methylphenyl)-3,5-dihydro-4*H*-imidazol-4-one (DMHBI-Fc, **32**) ... 21

1.2.56 (*Z*)-3-(4-(Dimethylamino)phenyl)-5-(4-hydroxy-3,5-dimethoxybenzylidene)-2-((*E*)-2-phenylvinyl)-3,5-dihydro-4*H*-imidazol-4-one (**33**) ... 21

1.2.57 (*Z*)-5-(4-Hydroxy-3,5-dimethoxybenzylidene)-2-((*E*)-2-phenylvinyl)-3-(4-(trimethylammonium)phenyl)-3,5-dihydro-4*H*-imidazol-4-one iodide (DMHBI-Styr$^+$, **34**) ... 22

1.3 RNA synthesis ... 22

1.3.1 *In vitro* transcription of RNA aptamers ... 22

1.4 UV/Vis spectroscopy ... 22

1.4.1 Melting curves ... 22

1.5 Fluorescence spectroscopy ... 23

1.5.1 Dye screening ... 23

1.5.2 Mutant screening ... 23

1.5.3 Competition assay ... 24

1.5.4 Metal ion dependence ... 24

1.5.5 Equilibrium binding titration ... 24

1.5.6 Association kinetics ... 25

1.5.7 Melting curves 25
1.6 NMR spectroscopy 25
1.6.1 H_2O/D_2O exchange 25
1.7 Isothermal titration calorimetry 25
2 Computational methods 27
2.1 Typical ORCA input file 27
3 Supporting Tables 28
4 Supporting Figures 30
5 NMR spectra 40
6 Cartesian coordinates of HBI derivatives 41
6.1 (*Z*)-5-(4-Hydroxy-3,5-dimethoxybenzylidene)-3-isopropyl-2-methyl-3,5-dihydro-4*H*-imidazol-4-one (DMHBI-*i*Pr, **3**) 41
6.2 (*Z*)-5-(4-Hydroxy-3,5-dimethoxybenzylidene)-2-methyl-3-(*trans*-4-methylcyclohexyl)-3,5-dihydro-4*H*-imidazol-4-one (DMHBI- MeCy, **5**) 41
6.3 (*Z*)-3-Benzyl-5-(4-hydroxy-3,5-dimethoxybenzylidene)-2-methyl-3,5-dihydro-4*H*-imidazol-4-one (DMHBI-Bn, **6**) 42
6.4 (*Z*)-5-(4-Hydroxy-3,5-dimethoxybenzylidene)-2-methyl-3-(4-methylphenyl)-3,5-dihydro-4*H*-imidazol-4-one (DMHBTI, **9**) 43
6.5 (*Z*)-5-(4-Hydroxy-3,5-dimethoxybenzylidene)-3-(4-methoxyphenyl)-2-methyl-3,5-dihydro-4*H*-imidazol-4-one (DMHBAI, **10**) 43
6.6 (*Z*)-5-(4-Hydroxy-3,5-dimethoxybenzylidene)-3-(4-trifluoromethylphenyl)-2-methyl-3,5-dihydro-4*H*-imidazol-4-one (DMHBTI^F, **11**) 44
6.7 (*Z*)-5-(4-Hydroxy-3,5-dimethoxybenzylidene)-3-(4-trifluoromethoxyphenyl)-2-methyl-3,5-dihydro-4*H*-imidazol-4-one (DMHBAI^F, **12**) 45
6.8 (DMHBI$^+$, **14**) 45
6.9 (*Z*)-3-(4-*tert*-Butylphenyl)-5-(4-hydroxy-3,5-dimethoxybenzylidene)-2-methyl-3,5-dihydro-4*H*-imidazol-4-one (DMHBI^C, **15**) 46
7 References 48

1 Experimental Procedures

1.1 General Information

1.1.1 Materials

All standard chemicals and solvents were purchased from commercial suppliers and were used without further purification. Organic solvents for optical spectroscopy were purchased from Acros Organics. Aluminum-backed plates coated with silica gel and a fluorescent indicator were used for thin layer chromatography (TLC). The plates were visualized with UV light. Additionally, exposing the plates to ammonia vapor rendered HBI compounds orange or violet. Silica gel 60, 0.032–0.063 mm (230–450 mesh) was used for column chromatography.

DNA templates for in vitro transcription were purchased from Microsynth and purified by denaturing PAGE (15–20% polyacrylamide). Ribonucleotide triphosphates (NTPs) were purchased from Jena Bioscience. T7 RNA polymerase was prepared in house following a published procedure with minor modifications (1).

1.1.2 NMR spectroscopy

NMR spectra were acquired on Bruker Avance III and Avance III HD spectrometers between 300 and 600 MHz as well as Varian Mercury Plus and Inova spectrometers between 300 and 600 MHz.

Chemical shifts (δ) in ppm are referenced to the solvent residual signals, an internal standard (^{1}H and ^{13}C) or on the unified scale (other nuclei) (2). Coupling constants (J) are reported in Hz with the following multiplet designations: s (singlet), d (doublet), t (triplet), q (quartet), m (multiplet), br (broad).

All spectral assignments were verified by additional 2D experiments.

1.1.3 Mass spectrometry

High resolution ESI mass spectra in positive or negative ion mode were acquired on Bruker micrOTOF, micrOTOF-Q III and maXis instruments.

1.2 Synthetic procedures for chromophores

DMHBI (**1**), DMHBI$^+$ (**14**), DMHBI-Imi (**25**) and DMHBO$^+$ (**36**) and their synthetic precursors were prepared as described previously (3).

1.2.1 General procedure A, imine synthesis with volatile amines

A suspension of the aldehyde (25.0 mmol, 1.00 eq.) and $MgSO_4$ (30.0 mmol, 1.20 eq.) in the amine (250 mmol, 10.0 eq.) was stirred (CH_2Cl_2 was added if necessary) at ambient temperature for 24 h. Afterwards, the solution was filtered over a Celite plug. The solids were rinsed with CH_2Cl_2 (3×20 mL) and the filtrate was evaporated under reduced pressure. The resulting product was usually sufficiently pure for all further reactions.

1.2.2 General procedure B, imine synthesis with non-volatile amines

A solution of the aldehyde (20.0 mmol, 1.00 eq.) and the amine (20.0 mmol, 1.00 eq.) in toluene (80 mL) was heated to reflux with a Dean-Stark trap for 16 h. Afterwards, the solvent was completely removed under reduced pressure. The resulting product was usually sufficiently pure for all further reactions.

1.2.3 General procedure C, cycloaddition reaction

A mixture of the imine (2.00 mmol, 1.00 eq.) and imidate (2.40 mmol, 1.20 eq) in either EtOH toluene or toluene (2 mL) was stirred at ambient temperature or at 120 °C, respectively, until TLC showed completion (usually 24 h, up to 5 d for some compounds). In case the product had precipitated, the solids were collected by filtration and washed with Et_2O (50 mL). Otherwise the reaction mixture was evaporated to dryness, and the crude product was purified by column chromatography.

1.2.4 General procedure D, Aldol condensation

The HBI derivative (200 µmol, 1.00 eq.), aldehyde (250 µmol, 1.25 eq.) and scandium triflate (30.0 µmol, 15.0 mol%) were dissolved in anhydrous dioxane (1 mL) in a closed vial. The mixture was stirred at 110 °C (oil bath temperature) until TLC showed completion (up to 48 h). Afterwards, the solvent was removed under reduced pressure. Purification of the residue by washing with MeOH or by column chromatography afforded the product.

1.2.5 4-Hydroxy-3,5-dimethoxy-*N*-ethylbenzaldimine (**S1**)

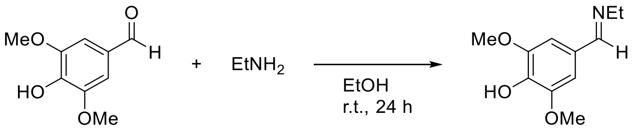

S1

The title compound was synthesized according to General procedure A on a 5.00 mmol scale using a 70% solution of $EtNH_2$ in H_2O and without the addition of $MgSO_4$. Pale yellow solid (816 mg, 4.50 mmol, 90%).

^{1}H NMR (400 MHz, $CDCl_3$): *δ* (ppm) = 8.15 (t, *J* = 1.3 Hz, 1 H, CHN), 6.99 (s, 2 H, Ph-2,6-H), 5.83 (s_{br}, 1 H, OH), 3.92 (s, 6 H, OCH_3), 3.62 (qd, *J* = 7.3, 1.3 Hz, 2 H, CH_2CH_3), 1.29 (t, *J* = 7.3 Hz, 3 H, CH_2CH_3);

^{13}C{^{1}H} NMR (100 MHz, $CDCl_3$): *δ* (ppm) = 160.2 (CHN), 147.2 (Ph-C3,5), 137.4 (Ph-C4), 127.7 (Ph-C1), 104.9 (Ph-C2,6), 56.4 (OCH_3), 55.5 (CH_2CH_3), 16.4 (CH_2CH_3);

HR-MS (ESI+): *m*/*z* calc. ($C_{11}H_{16}NO_3$, $[M+H]^+$): 210.1125, found: 210.1122.

1.2.6 4-Hydroxy-3,5-dimethoxy-*N*-isopropylbenzaldimine (S2)

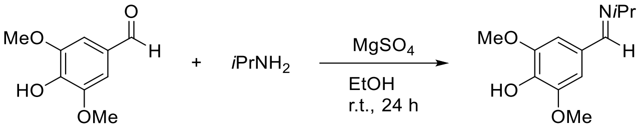

S2

The title compound was synthesized according to General procedure A on a 5.00 mmol scale. Orange foam (1.07 g, 4.79 mmol, 96%).

^{1}H NMR (400 MHz, $CDCl_3$): *δ* (ppm) = 8.17 (s, 1 H, CHN), 6.98 (s, 2 H, Ph-2,6-H), 3.92 (s, 6 H, OCH_3), 3.51 (hept, *J* = 6.4 Hz, 1 H, $CH(CH_3)_2$), 1.25 (d, *J* = 6.4 Hz, 6 H, $CH(CH_3)_2$);

^{13}C{^{1}H} NMR (100 MHz, $CDCl_3$): *δ* (ppm) = 158.2 (CHN), 147.4 (Ph-C3,5), 137.4 (Ph-C4), 128.0 (Ph-C1), 105.0 (Ph-C2,6), 61.6 ($CH(CH_3)_2$), 56.5 (OCH_3), 24.3 ($CH(CH_3)_2$);

HR-MS (ESI+): *m*/*z* calc. ($C_{12}H_{18}NO_3$, $[M+H]^+$): 224.1281, found: 224.1282.

1.2.7 4-Hydroxy-3,5-dimethoxy-*N*-(*tert*-butyl)benzaldimine (S3)

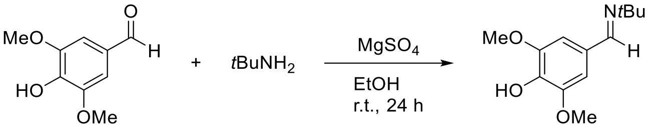

S3

The title compound was synthesized according to General procedure A on a 12.5 mmol scale. Due to its low stability the crude product was used in the next step without further characterization.

1.2.8 4-Hydroxy-3,5-dimethoxy-*N*-(*trans*-4-methylcyclohexyl)benzaldimine (S4)

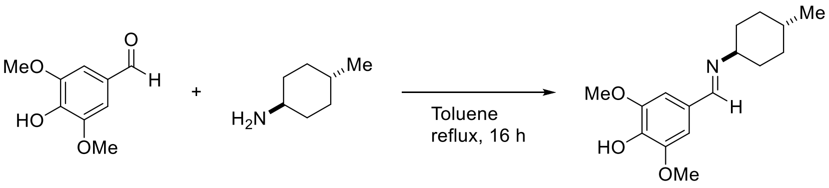

S4

The title compound was synthesized according to General procedure A on a 5.00 mmol scale. Yellow foam (1.39 g, 5.00 mmol, > 99%).

^{1}H NMR (300 MHz, $CDCl_3$): *δ* (ppm) = 8.19 (s, 1 H, CHN), 6.98 (s, 2 H, Ph-2,6-H), 3.93 (s, 6 H, OCH_3), 3.20–3.03 (m, 1 H, Cy-1-H), 1.83–1.70 (m, 4 H, Cy-2,6-H, Cy-3,5-H), 1.70–1.54 (m, 2 H, Cy-2,6-H), 1.52–1.33 (m, 1 H, Cy-4-H), 1.07 (td, *J* = 12.3, 3.6 Hz, 2 H, Cy-3,5-H), 0.92 (d, *J* = 6.5 Hz, 3 H, Cy-CH_3);

^{13}C{^{1}H} NMR (75 MHz, $CDCl_3$): *δ* (ppm) = 158.6 (CHN), 147.4 (Ph-C3,5), 128.4 (Ph-C1), 105.1 (Ph-C2,6), 70.0 (Cy-C1), 56.6 (OCH_3), 34.4 (Cy-C2,6), 33.9 (Cy-C2,6, Cy-C3,5), 32.1 (Cy-C4), 22.6 (Cy-CH_3);

HR-MS (ESI+): *m*/*z* calc. ($C_{16}H_{24}NO_3$, $[M+H]^+$): 278.1754, found: 278.1787.

1.2.9 4-Hydroxy-3,5-dimethoxy-*N*-benzylbenzaldimine (S5)

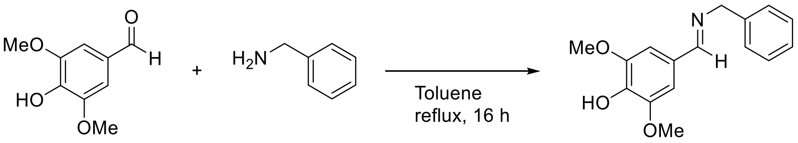

S5

The title compound was synthesized according to General procedure B on a 25.0 mmol scale. A first batch of the product crystallized from the reaction mixture after cooling to ambient temperature. It was isolated by filtration; the remainder was obtained by evaporation of the filtrate. Pale yellow solid (6.78 g, 25.0 mmol, > 99%).

^{1}H NMR (300 MHz, $CDCl_3$): *δ* (ppm) = 8.26 (t, *J* = 1.4 Hz, 1 H, CHN), 7.42–7.20 (m, 5 H, Bn-2,6H, Bn-3,5-H, Bn-4-H), 7.05 (s, 2 H, Ph-2,6-H), 5.84 (s_{br}, 1 H, OH), 4.81 (d, *J* = 1.3 Hz, 2 H, CH_2), 3.93 (s, 6 H, OCH_3);

$^{13}C\{^1H\}$ NMR (125 MHz, $CDCl_3$): *δ* (ppm) = 161.7 (CHN), 147.2 (Ph-C3,5), 139.4 (Bn-C1), 137.5 (Ph-C4), 128.6 (Bn-C3,5), 128.1 (Bn-C2,6), 127.8 (Ph-C1), 127.1 (Bn-C4), 105.3 (Ph-C2,6), 65.0 (CH_2), 56.6 (OCH_3);

HR-MS (ESI+): *m/z* calc. ($C_{16}H_{18}NO_3$, $[M+H]^+$): 272.1281, found: 272.1281.

1.2.10 4-Hydroxy-3,5-dimethoxy-*N*-(4-methoxybenzyl)benzaldimine (S6)

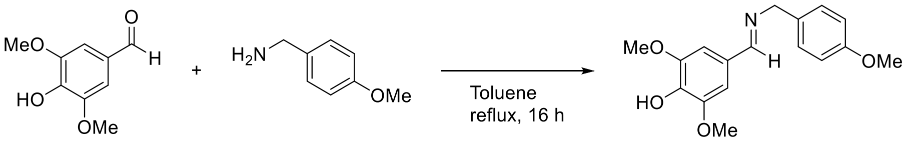

S6

The title compound was synthesized according to General procedure B on a 12.5 mmol scale. Pale yellow solid (3.77 g, 12.5 mmol, > 99%).

^{1}H NMR (600 MHz, $CDCl_3$): *δ* (ppm) = 8.22 (t, *J* = 1.4 Hz, 1 H, CHN), 7.26–7.22 (m, 2 H, NAr-2,6-H), 7.03 (s, 2 H, Ph-2,6-H), 6.90–6.87 (m, 2 H, NAr-3,5-H), 4.74 (d, *J* = 1.3 Hz, 2 H, CH_2), 3.89 (s, 6 H, Ph-OCH_3), 3.79 (s, 3 H, NAr-OCH_3);

$^{13}C\{^1H\}$ NMR (125 MHz, $CDCl_3$): *δ* (ppm) = 161.3 (CHN), 158.7 (NAr-C4), 147.3 (Ph-C3,5), 137.6 (Ph-C4), 131.4 (NAr-C1), 129.3 (NAr-C2,6), 127.7 (Ph-C1), 114.0 (NAr-C3,5), 105.2 (Ph-C2,6), 64.3 (CH_2), 56.5 (Ph-OCH_3), 55.4 (NAr-OCH_3);

HR-MS (ESI+): *m/z* calc. ($C_{17}H_{20}NO_4$, $[M+H]^+$): 302.1387, found: 302.1392.

1.2.11 4-Hydroxy-3,5-dimethoxy-*N*-phenylbenzaldimine (S7)

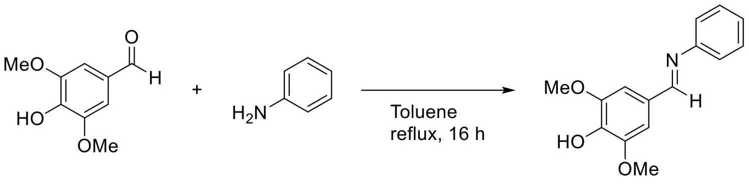

S7

The title compound was synthesized according to General procedure B on a 12.5 mmol scale. Dark yellow solid (3.11 g, 12.1 mmol, 97%).

^{1}H NMR (400 MHz, DMSO-*d_6*): *δ* (ppm) = 9.11 (s, 1 H, OH), 8.44 (s, 1 H, CHN), 7.43–7.36 (m, 2 H, NAr-3,5-H), 7.23 (s, 2 H, Ph-2,6-H), 7.23–7.17 (m, 3H, NAr-2,6-H, NAr-4-H), 3.84 (s, 6 H, OCH_3).

$^{13}C\{^1H\}$ NMR (100 MHz, DMSO-*d_6*): *δ* (ppm) = 160.5 (CHN), 151.9 (NAr-C1), 148.0 (Ph-C3,5), 139.2 (Ph-C4), 129.2 (NAr-C3,5), 126.6 (Ph-C1), 125.4 (NAr-C4), 120.9 (NAr-C2,6), 106.2 (Ph-C2,6), 56.0 (OCH_3) , 9, 10.;

HR-MS (ESI+): *m/z* calc. ($C_{15}H_{16}NO_3$, $[M+H]^+$): 258.11247, found: 258.11292.

1.2.12 4-Hydroxy-3,5-dimethoxy-*N*-(4-methylphenyl)benzaldimine (S8)

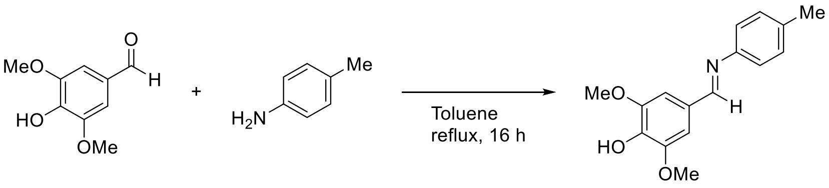

S8

The title compound was synthesized according to General procedure B on a 25.0 mmol scale. Yellow solid (6.78 g, 25.0 mmol, > 99%).

^{1}H NMR (300 MHz, $CDCl_3$): *δ* (ppm) = 8.34 (s, 1 H, CHN), 7.21–7.17 (m, 2 H, NAr-3,5-H), 7.17 (s, 2 H, Ph-2,6-H), 7.15–7.10 (m, 2 H, NAr-2,6-H), 5.90 (s_{br}, 1 H, OH), 3.97 (s, 6 H, OCH_3), 2.37 (s, 3 H, NAr-CH_3);

$^{13}C\{^1H\}$ NMR (75 MHz, $CDCl_3$): *δ* (ppm) = 159.4 (CHN), 149.6 (NAr-C1), 147.4 (Ph-C3,5), 138.0 (Ph-C4), 135.6 (NAr-C4), 129.9 (NAr-C3,5), 128.2 (Ph-C1), 120.9 (NAr-C2,6), 105.7 (Ph-C2,6), 56.6 (OCH_3), 21.1 (NAr-CH_3);

HR-MS (ESI+): *m/z* calc. ($C_{16}H_{18}NO_3$, $[M+H]^+$): 272.1281, found: 272.1283.

1.2.13 4-Hydroxy-3,5-dimethoxy-*N*-(4-methoxyphenyl)benzaldimine (S9)

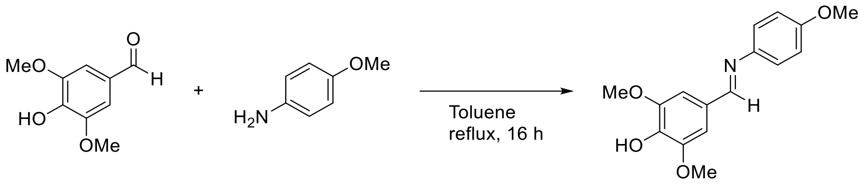

S9

The title compound was synthesized according to General procedure B on a 12.5 mmol scale. Off-white solid (3.36 g, 11.7 mmol, 94%).

^{1}H NMR (300 MHz, $CDCl_3$): *δ* (ppm) = 8.35 (s, 1 H, CHN), 7.25–7.18 (m, 2 H, NAr-2,6-H), 7.16 (s, 2 H, Ph-2,6-H), 6.96–6.89 (m, 2 H, NAr-3,5-H), 5.93 (s_{br}, 1 H, OH), 3.97 (s, 6 H, Ph-OCH_3), 3.83 (s, 3 H, NAr-OCH_3);

^{13}C{^{1}H} NMR (125 MHz, $CDCl_3$): *δ* (ppm) = 158.2 (CHN), 158.1 (NAr-C4), 147.3 (Ph-C3,5), 145.1 (NAr-C1), 137.9 (Ph-C4), 128.2 (Ph-C1), 122.1 (NAr-C2,6), 114.5 (NAr-C3,5), 105.6 (Ph-C2,6), 56.6 (Ph-OCH_3), 55.7 (NAr-OCH_3);

HR-MS (ESI+): *m/z* calc. ($C_{16}H_{18}NO_4$, $[M+H]^+$): 288.1230, found: 288.1237.

1.2.14 4-Hydroxy-3,5-dimethoxy-*N*-(4-trifluoromethylphenyl)benzaldimine (S10)

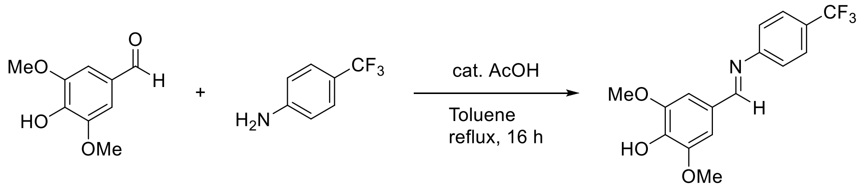

S10

A solution of 4-hydroxy-3,5-dimethoxybenzaldehyde (911 mg, 5.00 mmol, 1.00 eq.) and 4-trifluoromethylaniline (806 mg, 5.00 mmol, 1.00 eq.) in toluene (20 mL) was treated with AcOH (4 drops) and heated to reflux for 16 h. After cooling to ambient temperature, residual solids were removed by filtration over Celite and washed successively with toluene, $CHCl_3$ and MeOH (20 mL each). The filtrate was evaporated to afford the crude product (1.60 g, 4.92 mmol, 98%) as an off-white solid sufficiently pure for further reactions. An analytically pure sample was prepared by recrystallization from heptane/toluene (5:1, 1.25 g in 30 mL).

^{1}H NMR (300 MHz, $CDCl_3$): *δ* (ppm) = 8.29 (s, 1 H, CHN), 7.63 (m, 2 H, NAr-3,5-H), 7.24 (m, 2 H, NAr-2,6-H), 7.18 (s, 2 H, Ph-2,6-H), 6.14 (s_{br}, 1 H, OH), 3.95 (s, 6 H, OCH_3);

^{13}C{^{1}H} NMR (125 MHz, $CDCl_3$): *δ* (ppm) = 161.4 (CHN), 155.3 (NAr-C1), 147.4 (Ph-C3,5), 138.7 (Ph-C4), 127.5 (q, *J* = 32.5 Hz, NAr-C4), 127.5 (Ph-C1), 126.4 (q, *J* = 3.8 Hz, NAr-C3,5), 124.4 (q, *J* = 271.3 Hz, CF_3), 121.1 (NAr-C2,6), 106.1 (Ph-C2,6), 56.7 (OCH_3);

^{19}F NMR (282 MHz, $CDCl_3$): *δ* (ppm) = −62.0 (CF_3);

HR-MS (ESI+): *m/z* calc. ($C_{16}H_{15}F_3NO_3$, $[M+H]^+$): 326.0999, found: 326.1013.

1.2.15 4-Hydroxy-3,5-dimethoxy-*N*-(4-trifluoromethoxyphenyl)benzaldimine (S11)

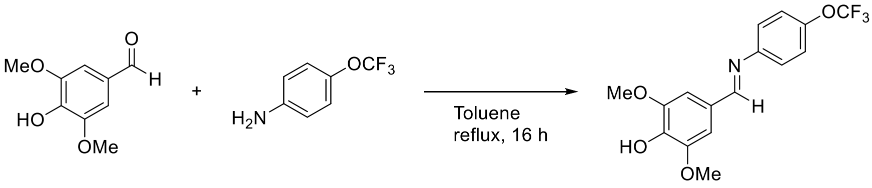

S11

The title compound was synthesized according to General procedure B on a 5.00 mmol scale. Brown solid (1.66 g, 4.86 mmol, 97%). Due to its low stability the crude product was used in the next step without further characterization.

1.2.16 4-Hydroxy-3,5-dimethoxy-*N*-(4-(*tert*-butyl)phenyl)benzaldimine (S12)

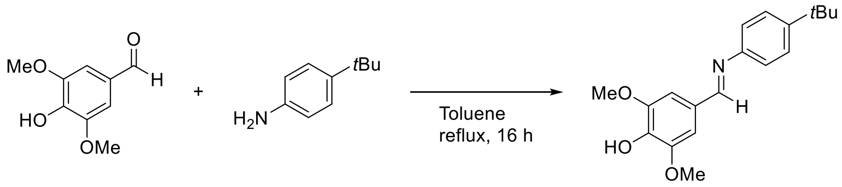

S12

The title compound was synthesized according to General procedure B on a 7.50 mmol scale. Off-white solid (2.35 g, 7.50 mmol, > 99%).

^{1}H NMR (300 MHz, $CDCl_3$): *δ* (ppm) = 8.35 (s, 1H, CHN), 7.47–7.35 (m, 2 H, NAr-2,6-H), 7.21–7.11 (m, 4 H, Ph-2,6-H, NAr-3,5-H), 5.92 (s_{br}, 1 H, OH), 3.97 (s, 6 H, OCH_3), 1.35 (s, 9 H, $C(CH_3)_3$);

^{13}C{^{1}H} NMR (75 MHz, $CDCl_3$): *δ* (ppm) = 159.4 (CHN), 149.5 (NAr-C4), 149.0 (NAr-C1), 147.4 (Ph-C3,5), 138.1 (Ph-C4), 128.2 (Ph-C1), 126.1 (NAr-C2,6), 120.6 (NAr-C3,5), 105.8 (Ph-C2,6), 56.6 (OCH_3), 34.6 ($\mathit{C}(CH_3)_3$), 31.6 ($C(\mathit{CH}_3)_3$);

HR-MS (ESI+): *m*/*z* calc. ($C_{19}H_{24}NO_3$, $[M+H]^+$): 314.1751, found: 314.1761.

1.2.17 4-Hydroxy-3-methoxy-*N*-(4-trifluoromethoxyphenyl)benzaldimine (**S13**)

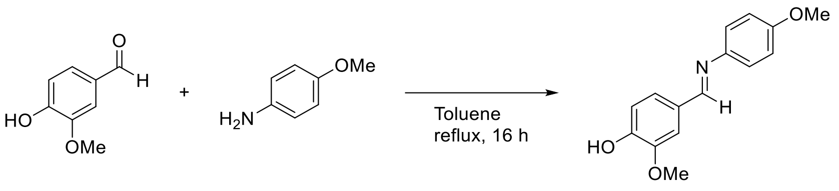

S13

The title compound was synthesized according to General procedure B on a 12.5 mmol scale. It crystallized from the reaction mixture after cooling to ambient temperature. Off-white solid (2.99 g, 11.6 mmol, 93%).

^{1}H NMR (400 MHz, DMSO-*d*$_6$): *δ* (ppm) = 9.66 (s_{br}, 1 H, OH), 8.45 (s, 1 H, CHN), 7.50 (d, *J* = 1.9 Hz, 1 H, Ph-2-H), 7.30 (dd, *J* = 8.2, 1.9 Hz, 1 H, Ph-6-H), 7.24–7.20 (m, 2 H, NAr-3,5-H), 6.98–6.92 (m, 2 H, NAr-2,6-H), 6.88 (d, *J* = 8.2 Hz, 1 H, Ph-5-H), 3.84 (s, 3 H, Ph-OCH_3), 3.76 (s, 3 H, NAr-OCH_3).;

^{13}C{^{1}H} NMR (100 MHz, DMSO-*d*$_6$): *δ* (ppm) = 158.1 (CHN), 157.4 (NAr-C1), 149.8 (Ph-C4), 147.9 (Ph-C3), 144.7 (NAr-C4), 128.1 (Ph-C1), 123.7 (Ph-C6), 122.1 (NAr-C3,5), 115.3 (Ph-C5), 114.4 (NAr-C2,6), 110.2 (Ph-C2), 55.5 (Ph-OCH_3), 55.3, (NAr-OCH_3);

HR-MS (ESI+): *m*/*z* calc. ($C_{15}H_{16}NO_3$, $[M+H]^+$): 258.11247, found: 258.11302.

1.2.18 3,5-dimethoxy-*N*-(4-methoxyphenyl)benzaldimine (**S14**)

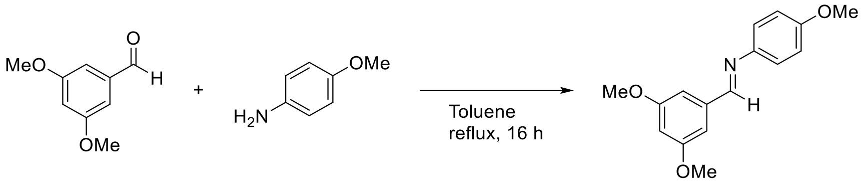

S14

The title compound was synthesized according to General procedure B on a 5.00 mmol scale. Light brown solid (1.36 g, 5.00 mmol, > 99%).

^{1}H NMR (300 MHz, $CDCl_3$): *δ* (ppm) = 8.39 (d, *J* = 0.4 Hz, 1 H, CHN), 7.30–7.18 (m, 2 H, NAr-2,6-H), 7.06 (dd, *J* = 2.3, 0.4 Hz, 2 H, Ph-2,6-H), 6.97–6.90 (m, 2 H, NAr-3,5-H), 6.57 (t, *J* = 2.3 Hz, 1 H, Ph-4-H), 3.86 (s, 6 H, Ph-OCH_3), 3.83 (s, 3 H, NAr-OCH_3);

^{13}C{^{1}H} NMR (75 MHz, $CDCl_3$): *δ* (ppm) = 161.2 (Ph-C3,5), 158.5 (NAr-C4), 158.4 (CHN), 144.8 (NAr-C1), 138.6 (Ph-C1), 122.4 (NAr-C2,6), 114.5 (NAr-C3,5), 106.4 (Ph-C2,6), 104.1 (Ph-C4), 55.7 (Ph-OCH_3), 55.6 (NAr-OCH_3);

HR-MS (ESI+): *m*/*z* calc. ($C_{16}H_{18}NO_3$, $[M+H]^+$): 272.1281, found: 272.1284.

1.2.19 Ethyl 3-phenylpropionimidate hydrochloride (**S15**)

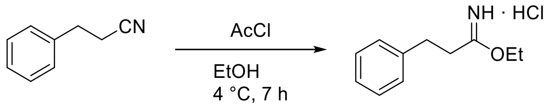

S15

The title compound was prepared according to a previously reported procedure (4): 3-Phenylpropionitrile (787 mg, 6.00 mmol, 1.00 eq.) was dissolved in EtOH (4.2 mL, 72.0 mmol, 12.0 eq.) and cooled to 0 °C. AcCl (3.41 mL, 48.0 mmol, 8.00 eq.) was added dropwise and the resulting yellow solution was stirred at 4 °C for 7 h. The product was precipitated by addition of Et_2O (20 mL), collected by filtration and washed with Et_2O (30 mL), giving a white, crystalline solid (963 mg, 4.51 mmol, 75%).

^{1}H NMR (500 MHz, DMSO-*d*$_6$): *δ* (ppm) = 11.95 (s_{br}, 1 H, NH · HCl), 11.08 (s_{br}, 1 H, NH · HCl), 7.34–7.29 (m, 2 H, Ph-3,5-H), 7.28–7.20 (m, 3 H, Ph-2,6-H, Ph-4-H), 4.37 (q, *J* = 7.0 Hz, 2 H, $O\mathit{CH}_2CH_3$), 2.94 (s, 4 H, $PhCH_2CH_2$), 1.30 (t, *J* = 7.0 Hz, 3 H, $OCH_2\mathit{CH}_3$);

^{13}C{^{1}H} NMR (125 MHz, DMSO-*d*$_6$): *δ* (ppm) = 178.3 (CNO), 138.9 (Ph-C1), 128.6 (Ph-C3,5), 128.3 (Ph-C2,6), 126.6 (Ph-C4), 69.0 ($O\mathit{C}H_2CH_3$), 34.1 ($PhCH_2\mathit{C}H_2$), 30.5 ($Ph\mathit{C}H_2CH_2$), 13.3 ($OCH_2\mathit{C}H_3$);

HR-MS (ESI+): *m/z* calc. ($C_{11}H_{16}NO$, $[M-Cl]^+$): 178.1226, found: 178.1230.

1.2.20 Methyl (*Z*)-2-((1-ethoxyethylidene)amino)acetate (**S16**)

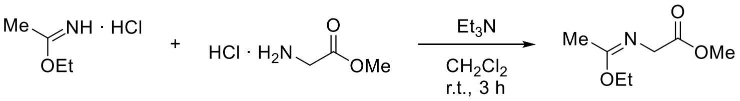

S16

To a suspension of ethyl acetimidate hydrochloride (4.63 g, 37.5 mmol, 1.00 eq.) and methyl glycinate hydrochloride (4.71 g, 37.5 mmol, 1.00 eq.) in dry CH_2Cl_2 (150 mL) was added Et_3N (5.2 mL, 37.5 mmol, 1.00 eq.). The resulting mixture was stirred at ambient temperature for 3 h. Afterwards, it was washed with H_2O (2×150 mL) and brine (150 mL) and the organic phase was dried over $MgSO_4$. Evaporation of the solvent under reduced pressure afforded the title compound as a colorless liquid (5.08 g, 31.9 mmol, 85%). Spectral data matched those reported previously (5). The product can be stored under an inert atmosphere at –20 °C for several weeks without decomposition.

^{1}H NMR (300 MHz, $CDCl_3$): *δ* (ppm) = 4.10 (q, *J* = 7.1 Hz, 2 H, OCH_2CH_3), 4.05 (s, 2 H, NCH_2), 3.73 (s, 3 H, OCH_3), 1.87 (s, 3 H, CCH_3), 1.26 (t, *J* = 7.1 Hz, 3 H, OCH_2CH_3);

HR-MS (ESI+): *m/z* calc. ($C_7H_{13}NNaO_3$, $[M+Na]^+$): 182.0788, found: 182.0788.

1.2.21 Methyl (*Z*)-2-((1-ethoxy-3-phenylpropylidene)amino)acetate (**S17**)

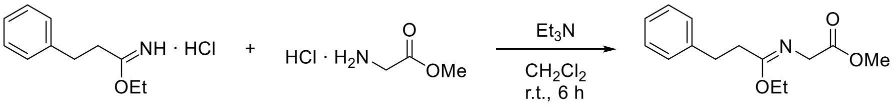

S15 **S17**

To a suspension of ethyl 3-phenylpropionimidate hydrochloride (S15, 748 mg, 3.50 mmol, 1.00 eq.) and methyl glycinate hydrochloride (439 mg, 3.50 mmol, 1.00 eq.) in dry CH_2Cl_2 (14 mL) was added Et_3N (0.49 mL, 3.50 mmol, 1.00 eq.). The resulting mixture was stirred at ambient temperature for 6 h. Afterwards, it was washed with H_2O (2×14 mL) and brine (14 mL) and the organic phase was dried over $MgSO_4$. Evaporation of the solvent under reduced pressure afforded the title compound as a pale yellow oil (631 mg, 2.53 mmol, 72%).

^{1}H NMR (300 MHz, $CDCl_3$): *δ* (ppm) = 7.35–7.25 (m, 2 H, Ph-3,5-H), 7.25–7.12 (m, 4 H, Ph-2,6-H, Ph-4-H), 4.12 (q, *J* = 7.1 Hz, 2 H, OCH_2CH_3), 3.92 (s, 2 H, NCH_2), 3.70 (s, 3 H, OCH_3), 2.87 (dd, *J* = 9.0, 6.7 Hz, 2 H, $PhCH_2CH_2$), 2.52–2.45 (m, 2 H, $PhCH_2CH_2$), 1.27 (t, *J* = 7.1 Hz, 3 H, OCH_2CH_3);

^{13}C{^{1}H} NMR (125 MHz, $CDCl_3$): *δ* (ppm) = 171.6 ($COOCH_3$), 165.8 (CNO), 140.8 (Ph-C1), 128.6 (Ph-C3,5), 128.4 (Ph-C2,6), 126.4 (Ph-C4), 61.1 (OCH_2CH_3), 52.1 (OCH_3), 50.7 (NCH_2), 32.3 ($PhCH_2CH_2$), 31.0 ($PhCH_2CH_2$), 14.4 (OCH_2CH_3);

HR-MS (ESI+): *m/z* calc. ($C_{14}H_{20}NO_3$, $[M+H]^+$): 250.1438, found: 250.1488.

1.2.22 (*Z*)-4-(4-Acetyloxy-3-bromo-5-methoxybenzylidene)-2-methyl-5(4*H*)-oxazolone (**S18**)

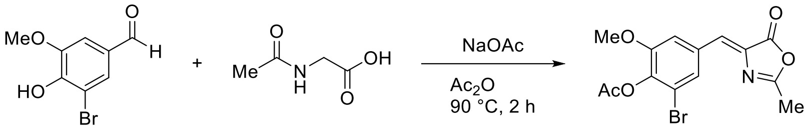

S18

3-Bromo-4-hydroxy-5-methoxybenzaldehyde (1.00 g, 4.33 mmol, 1.00 eq.), *N*-acetylglycine (507 mg, 4.33 mmol, 1.00 eq.) and NaOAc (355 mg, 4.33 mmol, 1.00 eq.) were suspended in Ac_2O (3 ml). The mixture was stirred at 90 °C for 2 h and then cooled to ambient temperature. It was diluted with EtOH (3.5 ml) and kept at 0 °C for 16 h. The precipitate was filtered off and rinsed with cold EtOH (2 ml), hot H_2O (5 ml) and hexane (2×5 ml) to afford the title compound as a yellow solid (1.05 g, 2.96 mmol, 68%).

^{1}H NMR (600 MHz, $CDCl_3$): *δ* (ppm) = 7.84 (d, *J* = 1.8 Hz, 1 H, Ph-2-H), 7.80 (d, *J* = 1.8 Hz, 1 H, Ph-6-H), 6.98 (s, 1 H, benzylidene-H), 3.89 (s, 3 H, OCH_3), 2.41 (s, 3 H, CCH_3), 2.38 (s, 3 H, $OC(O)CH_3$);

^{13}C{^{1}H} NMR (125 MHz, $CDCl_3$): *δ* (ppm) = 167.7 ($OC(O)CH_3$), 167.4 (Oxa-C5), 167.1 (Oxa-C2), 152.7 (Ph-C5), 140.1 (Ph-C4), 133.6 (Oxa-C4), 132.7 (benzylidene-C), 129.0 (Ph-C1), 128.7 (Ph-C2), 117.7 (Ph-C3), 114.4 (Ph-C6), 56.5 (OCH_3), 20.6 ($OC(O)CH3$), 15.9 (CCH_3);

HR-MS (ESI+): *m/z* calc. ($C_{15}H_{15}BrNO_5$, $[M+MeOH-H]^-$): 384.0088, found: 384.0079.

1.2.23 (*Z*)-4-(4-Hydroxy-3,5-dimethoxybenzylidene)-1-methyl-5-oxo-4,5-dihydro-1*H*-imidazole-2-carbaldehyde (S19)

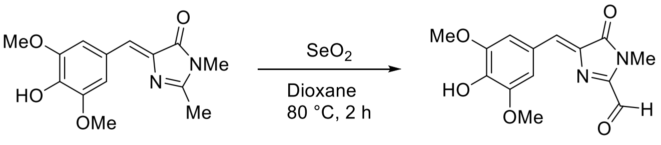

1 **S19**

DMHBI (**1**, 414 mg, 1.50 mmol, 1.00 eq.) and SeO_2 (200 mg, 1.80 mmol, 1.20 eq.) were suspended in dioxane (25 mL) and heated to reflux for 2 h. While still hot, the supernatant was decanted off from the deposited solids and the solvent was removed under reduced pressure. After purification by column chromatography ($CHCl_3$/EtOH 98:2 + 1% AcOH) the title compound was obtained as a red solid (884 mg, 3.05 mmol, 74%).

^{1}H NMR (500 MHz, $CDCl_3$): *δ* (ppm) = 9.75 (s, 1 H, CHO), 7.61 (s, 2 H, Ph-2,6-H), 7.45 (s, 1 H, benzylidene-H), 6.11 (s, 1 H, OH), 3.98 (s, 6 H, OCH_3), 3.49 (s, 3 H, NCH_3);

^{13}C{^{1}H} NMR (125 MHz, $CDCl_3$): *δ* (ppm) = 185.4 (CHO), 170.3 (Imi-C4), 153.7 (Imi-C2), 147.5 (Ph-C3,5), 139.9 (Ph-C4), 137.7 (benzylidene-C), 137.3 (Imi-C5), 125.5 (Ph-C1), 111.2 (Ph-C2,6), 56.6 (OCH_3), 28.1 (NCH_3);

HR-MS (ESI+): *m*/*z* calc. ($C_{15}H_{19}N_2O_6$, $[M+MeOH+H]^+$): 323.1238, found: 323.1233.

1.2.24 (Hydroxymethyl)ferrocene (S20)

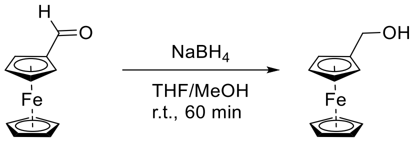

S20

Ferrocene carbaldehyde (562 mg, 2.63 mmol, 1.00 eq.) was dissolved in a mixture of THF (25 ml) and MeOH (5 ml) at ambient temperature. $NaBH_4$ (99.9 mg, 2.64 mmol, 1.01 eq.) was added in 5 portions over the course of 30 min; stirring was continued for 30 min. After removal of the solvent, the residue was taken up in EtOAc (25 ml), washed with H_2O (2×10 ml) and brine (10 ml). The organic phase was dried over Na_2SO_4 and solvent was evaporated under reduced pressure to afford the title compound as a yellow, crystalline solid (554 mg, 2.56 mmol, 97%). Spectral data matched those reported previously (6).

^{1}H NMR (500 MHz, $CDCl_3$): *δ* (ppm) = 4.33 (s, 2 H, CH_2), 4.24 (t, *J* = 1.9 Hz, 2 H, Fc-H), 4.18 (s, 5 H, Fc-H), 4.18 (t, *J* = 1.9 Hz, 2 H, Fc-H), 1.58 (s, 1 H, OH);

^{13}C{^{1}H} NMR (125 MHz, $CDCl_3$): *δ* (ppm) = 88.6 (Fc-C1), 68.4 (Fc-C), 68.4 (Fc-C), 68.0 (Fc-C), 60.9 (CH_2);

HR-MS (ESI+): *m*/*z* calc. ($C_{11}H_{10}Fe$, $[M-H_2O]^+$): 199.0205, found: 199.0211.

1.2.25 Triphenylphosphonium bromide (S21)

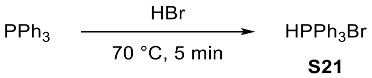

S21

Triphenylphosphine (13.1 g, 50.0 mmol, 1.00 eq.) was suspended in 48% aq. HBr (35 ml) and stirred at 70 °C for 5 min. The resulting clear solution was cooled to ambient temperature and extracted with $CHCl_3$ (3×15 ml). The combined organic phases were dried over Na_2SO_4. Evaporation of the solvent under reduced pressure afforded the title compound as a white solid (15.6 g, 45.5 mmol, 91%). Spectral data matched those reported previously (7).

^{1}H NMR (500 MHz, $CDCl_3$): *δ* (ppm) = 12.15 (s_{br}, 1 H, PH), 7.74–7.66 (m, 6 H, Ar-H), 7.67–7.54 (m, 3 H, Ar-4-H), 7.57–7.47 (m, 6 H, Ar-H);

^{13}C{^{1}H} NMR (125 MHz, $CDCl_3$): *δ* (ppm) = 134.1 (d, *J* = 13.6 Hz, Ph-C4), 133.0 (Ph-C2,6), 129.8 (d, *J* = 11.0 Hz, Ph-C3,5), 123.7 (Ph-C1);

^{31}P NMR (203 MHz, $CDCl_3$): *δ* (ppm) = −9.07 (PH);

HR-MS (ESI+): *m*/*z* calc. ($C_{18}H_{16}P$, $[M-Br]^+$): 263.0984, found: 263.0987.

1.2.26 (Ferrocenylmethyl)triphenylphosphonium bromide (S22)

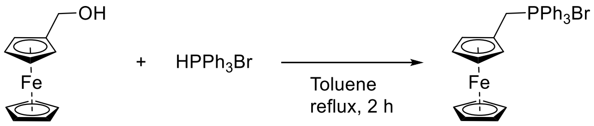

S20 **S21** **S22**

A suspension of (hydroxymethyl)ferrocene (**S20**, 350 mg, 1.62 mmol, 1.00 eq.) and triphenylphosphonium bromide (**S21**, 556 mg, 1.62 mmol, 1.00 eq.) in toluene (100 ml) was heated to reflux with a Dean-Stark trap for 2 h. After cooling to ambient temperature, the precipitate was filtered off and washed with cold Et_2O (10 ml) to afford the title compound as a yellow solid (620 mg, 1.15 mmol, 71%). Spectral data matched those reported previously (8).

^{1}H NMR (500 MHz, $CDCl_3$): *δ* (ppm) = 7.73 (m, 9 H), Ph-H, 7.64 (m, 6 H, Ph-H), 5.08 (s, 2 H), 4.37 (s, 5 H, Fc-H), 4.04 (s, 2 H), 3.97 (s, 2 H);

^{13}C{^{1}H} NMR (125 MHz, $CDCl_3$): *δ* (ppm) = 135.0 (d, *J* = 2.9 Hz, Ph-C4), 134.8 (d, *J* = 9.8 Hz, Ph-C2,6), 130.4 (d, *J* = 12.4 Hz, Ph-C3,5), 118.3 (d, *J* = 84.8 Hz, Ph-C1), 73.8 (Fc-C1), 71.1 (Fc-C), 70.4 (Fc-C), 68.9 (Fc-C), 29.3 (d, *J* = 42.5 Hz, CH_2);

^{31}P{^{1}H} NMR (203 MHz, $CDCl_3$): *δ* (ppm) = 19.31 (P);

HR-MS (ESI+): *m/z* calc. ($C_{29}H_{26}FeP$, $[M-Br]^+$): 461.1116, found: 461.1118.

1.2.27 Benzyltriphenylphosphonium bromide (S23)

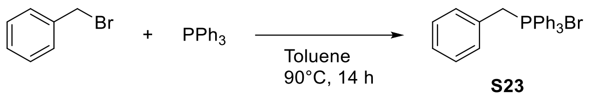

Benzyl bromide (5.94 ml, 50.0 mmol, 1.00 eq.) and triphenylphosphine (13.1 g, 50.0 mmol, 1.00 eq.) were suspended in toluene (125 ml) and stirred at 90 °C for 15 h. After cooling to ambient temperature, the precipitate was filtered off and washed with Et_2O (3×20 ml) to afford the title compound as a white solid (19.5 g, 45.1 mmol, 90%). Spectral data matched those reported previously (9).

^{1}H NMR (400 MHz, $CDCl_3$): *δ* (ppm) = δ 7.80–7.70 (m, 3 H, Ph-4-H), 7.76–7.65 (m, 6 H, Ph-2,6-H), 7.66–7.56 (m, 6 H, Ph-3,5-H), 7.25–7.15 (m, 1 H, Bn-4-H), 7.14–7.06 (m, 2 H, Bn-3,5-H), 7.11–7.03 (m, 2 H, Bn-2,6*H), 5.35 (d, *J* = 14.4 Hz, 2 H, CH_2);

^{13}C{^{1}H} NMR (100 MHz, $CDCl_3$): *δ* (ppm) = 135.1 (d, *J* = 3.1 Hz, Ph-C4), 134.5 (d, *J* = 9.8 Hz, Ph-C2,6), 131.6 (d, *J* = 5.5 Hz, Bn-C2,6), 130.3 (d, *J* = 12.6 Hz, Ph-C3,5), 128.9 (d, *J* = 3.4 Hz, Bn-C3,5), 128.5 (d, *J* = 3.9 Hz, Bn-C4), 127.2 (d, *J* = 8.6 Hz, Bn-C1), 117.9 (d, *J* = 85.7 Hz, Ph-C1), 30.9 (d, *J* = 47.0 Hz, CH_2);

^{31}P{^{1}H} NMR (162 MHz, $CDCl_3$): *δ* (ppm) = 23.14 (P);

1.2.28 (*Z*)-3-Ethyl-5-(4-hydroxy-3,5-dimethoxybenzylidene)-2-methyl-3,5-dihydro-4*H*-imidazol-4-one (DMHBI-Et, 2)

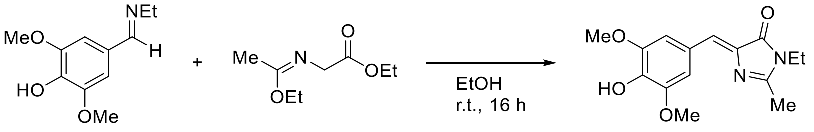

S1 **S16** **2**

The title compound was synthesized according to General procedure C on a 2.00 mmol scale. After purification by column chromatography ($CHCl_3$/EtOH 99:1–96:4 + 1% AcOH) it was obtained as a yellow solid (202 mg, 697 µmol, 35%).

^{1}H NMR (400 MHz, $CDCl_3$): *δ* (ppm) = 7.50 (s, 2 H, Ph-2,6-H), 7.01 (q, *J* = 0.6 Hz, 1H, benzylidene-H), 5.91 (s_{br}, 1 H, OH), 3.95 (s, 6 H, OCH_3), 3.67 (q, *J* = 7.3 Hz, 2 H, CH_2CH_3), 2.40 (d, *J* = 0.6 Hz, 3 H, CCH_3), 1.25 (t, *J* = 7.3 Hz, 3 H, CH_2CH_3);

^{13}C{^{1}H} NMR (100 MHz, $CDCl_3$): *δ* (ppm) = 170.4 (Imi-C4), 161.4 (Imi-C2), 147.2 (Ph-C3,5), 137.5 (Ph-C4), 136.7 (Imi-C5), 128.0 (benzylidene-C), 125.9 (Ph-C1), 109.5 (Ph-C2,6), 56.5 (OCH_3), 35.5 (CH_2CH_3), 15.8 (CCH_3), 14.8 (CH_2CH_3);

HR-MS (ESI+): *m/z* calc. ($C_{15}H_{19}N_2O_4$, $[M+H]^+$): 291.13353, found: 291.13393.

1.2.29 (*Z*)-5-(4-Hydroxy-3,5-dimethoxybenzylidene)-3-isopropyl-2-methyl-3,5-dihydro-4*H*-imidazol-4-one (DMHBI-*i*Pr, 3)

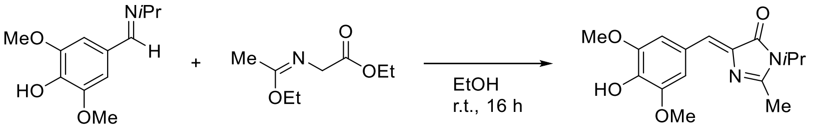

S2 **S16** **3**

The title compound was synthesized according to General procedure C on a 2.00 mmol scale. Brown solid (563 mg, 1.86 mmol, 93%).

^{1}H NMR (400 MHz, $CDCl_3$): *δ* (ppm) = 7.50–7.47 (m, 2 H, Ph-2,6-H), 6.95 (q, *J* = 0.6 Hz, 1 H, benzylidene-H), 5.94 (s_{br}, 1H, OH), 4.26 (hept, *J* = 6.9 Hz, 1 H, $CH(CH_3)_2$), 3.94 (s, 6 H, OCH_3), 2.43 (d, *J* = 0.6 Hz, 3 H, CCH_3), 1.47 (d, *J* = 7.0 Hz, 6 H, $CH(CH_3)_2$);

^{13}C{^{1}H} NMR (100 MHz, $CDCl_3$): *δ* (ppm) = 170.7 (Imi-C4), 161.7 (Imi-C2), 147.2 (Ph-C3,5), 137.4 (Ph-C4), 127.5 (benzylidene-C), 126.0 (Ph-C1), 109.4 (Ph-C2,6), 56.5 (OCH_3), 45.5 ($CH(CH_3)_2$), 20.7 ($CH(CH_3)_2$), 17.2 (CCH_3);

HR-MS (ESI+): *m/z* calc. ($C_{16}H_{21}N_2O_4$, $[M+H]^+$): 305.1496, found: 305.1492.

1.2.30 (*Z*)-3-(*tert*-Butyl)-5-(4-hydroxy-3,5-dimethoxybenzylidene)-2-methyl-3,5-dihydro-4*H*-imidazol-4-one (DMHBTI-*t*Bu, **4**)

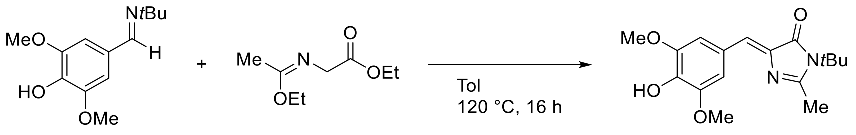

S3 **S16** **4**

The title compound was synthesized according to General procedure C on a 2.00 mmol scale. After purification by column chromatography (Hex/EtOAc 100:0–0:100 + 1% AcOH) the crude product was obtained as a yellow solid. Residual impurities were removed by sublimation (120 °C, 0.001 mbar). Yellow foam (118 mg, 0.37 mmol, 18%).

^{1}H NMR (600 MHz, $CDCl_3$): *δ* (ppm) = 7.49 (s, 2 H, Ph-2,6-H), 6.89 (s, 1 H, benzylidene-H), 5.85 (s, 1 H, OH), 3.94 (s, 6 H, OCH_3), 2.55 (s, 3 H, CCH_3), 1.63 (s, 9 H, $C(CH_3)_3$);

^{13}C{^{1}H} NMR (125 MHz, $CDCl_3$): *δ* (ppm) = 171.9 (Imi-C4), 162.4 (Imi-C2), 147.1 (Ph-C3,5), 137.2 (Ph-C4, Imi-C5), 126.8 (benzylidene-C), 126.2 (Ph-C1), 109.3 (Ph-C2,6), 57.7 (*C*$(CH_3)_3$), 56.5 (OCH_3), 29.9 ($C(CH_3)_3$), 22.2 (CCH_3);

HR-MS (ESI+): *m*/*z* calc. ($C_{17}H_{23}N_2O_4$, $[M+H]^+$): 319.1652, found: 319.1654.

1.2.31 (*Z*)-5-(4-Hydroxy-3,5-dimethoxybenzylidene)-2-methyl-3-(*trans*-4-methylcyclohexyl)-3,5-dihydro-4*H*-imidazol-4-one (DMHBI-MeCy, **5**)

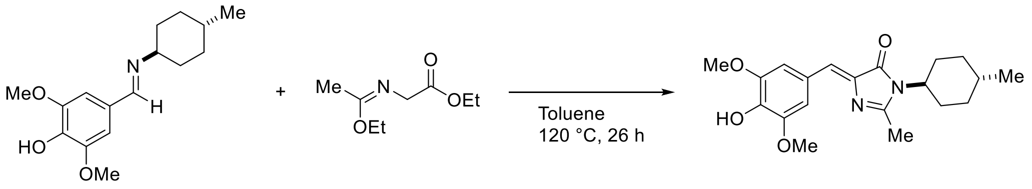

S4 **S16** **5**

The title compound was synthesized according to General procedure C on a 2.00 mmol scale. After purification by column chromatography (CH_2Cl_2/MeOH 99:1–96:4 + 1% AcOH) it was obtained as a light brown solid (286 mg, 799 µmol, 40%).

^{1}H NMR (600 MHz, $CDCl_3$): *δ* (ppm) = 7.49 (s, 2 H, Ph-2,6-H), 6.94 (s, 1 H, benzylidene-H), 5.86 (s, 1 H, OH), 3.95 (s, 6 H, OCH_3), 3.76 (tt, *J* = 12.4, 4.0 Hz, 1 H, Cy-1-H), 2.43 (s, 3 H, CCH_3), 2.18 (qd, *J* = 12.9, 3.6 Hz, 2 H, Cy-2,6-H^{ax}), 1.86–1.80 (m, 2 H, Cy-3,5-H^{eq}), 1.73 (dd, *J* = 13.4, 3.7 Hz, 2 H, Cy-2,6-H^{eq}), 1.54–1.41 (m, 1 H, Cy-4-H), 1.06 (qd, *J* = 13.2, 3.5 Hz, 2 H, Cy-3,5-H^{ax}), 0.93 (d, *J* = 6.5 Hz, 3 H, Cy-CH_3);

^{13}C{^{1}H} NMR (150 MHz, $CDCl_3$): *δ* (ppm) = 170.8 (Imi-C4), 161.9 (Imi-C2), 147.1 (Ph-C3,5), 137.3 (Ph-C4), 127.4 (Ph-C1, benzylidene-C), 126.0 (Imi-C5), 109.4 (Ph-C2,6), 56.5 (OCH_3), 53.9 (Cy-C1), 34.7 (Cy-C3,5), 31.7 (Cy-C4), 30.2 (Cy-C2,6)', 22.3 (Cy-CH_3), 17.4 (CCH_3);

HR-MS (ESI+): *m*/*z* calc. ($C_{20}H_{27}N_2O_4$, $[M+H]^+$): 359.1965, found: 359.1964.

1.2.32 (*Z*)-3-Benzyl-5-(4-hydroxy-3,5-dimethoxybenzylidene)-2-methyl-3,5-dihydro-4*H*-imidazol-4-one (DMHBI-Bn, **6**)

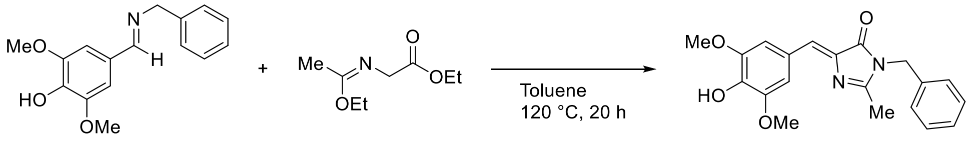

S5 **S16** **6**

The title compound was synthesized according to General procedure C on a 2.50 mmol scale. After purification by column chromatography ($CHCl_3$/EtOH 99:1–96:4 + 1% AcOH) it was obtained as a yellow solid (678 mg, 1.92 mmol, 77%).

^{1}H NMR (500 MHz, $CDCl_3$): *δ* (ppm) = 7.52 (s, 2 H, Ph-2,6-H), 7.36–7.30 (m, 2 H, Bn-3,5-H), 7.30–7.26 (m, 1 H, Bn-4-H), 7.24–7.20 (m, 2 H, Bn-2,6-H), 7.09 (q, *J* = 0.6 Hz, 1 H, benzylidene-H), 5.91 (s, 1 H, OH), 4.83 (s, 2 H, CH_2), 3.95 (s, 6 H, OCH_3), 2.25 (d, *J* = 0.6 Hz, 3 H, CCH_3);

^{13}C{^{1}H} NMR (125 MHz, $CDCl_3$): *δ* (ppm) = 170.7 (Imi-C4), 161.4 (Imi-C2), 147.2 (Ph-C3,5), 137.5 (Ph-C4), 136.9 (Imi-C5), 136.3 (Bn-C1), 129.1 (Bn-C3,5), 128.7 (benzylidene-C), 128.0 (Bn-C4), 127.1 (Bn-C2,6), 125.9 (Ph-C1), 109.5 (Ph-C2,6), 56.5 (OCH_3), 44.0 (CH_2), 16.3 (CCH_3);

HR-MS (ESI+): *m*/*z* calc. ($C_{21}H_{21}N_2O_4$, $[M+H]^+$): 353.1496, found: 353.1508;

TLC ($CHCl_3$/EtOH 96:4 + 1% AcOH): R_f = 0.56.

1.2.33 (*Z*)-5-(4-Hydroxy-3,5-dimethoxybenzylidene)-3-(4-methoxybenzyl)-2-methyl-3,5-dihydro-4*H*-imidazol-4-one (DMHBI-PMBn, 7)

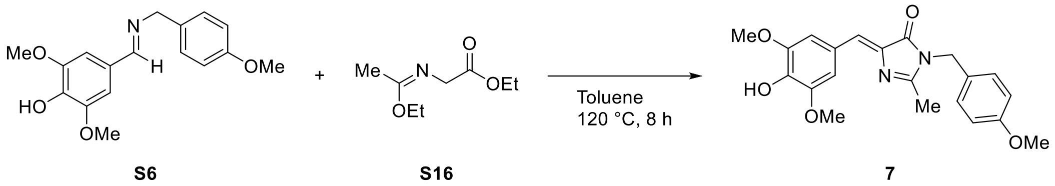

The title compound was synthesized according to General procedure C on a 2.00 mmol scale. After purification by column chromatography ($CHCl_3$/EtOH 99:1–96:4 + 1% AcOH) it was obtained as a yellow foam (509 mg, 1.33 mmol, 67%).

^{1}H NMR (600 MHz, $CDCl_3$): *δ* (ppm) = 7.50 (s, 2 H, Ph-2,6-H), 7.18–7.13 (m, 2 H, Bn-2,6-H), 7.06 (q, *J* = 0.6 Hz, 1 H, benzylidene-H), 6.88–6.82 (m, 2 H, Bn-3,5-H), 6.00 (s_{br}, 1 H, OH), 4.75 (s, 2 H, CH_2), 3.93 (s, 6 H, Ph-OCH_3), 3.78 (s, 3 H, Bn-OCH_3), 2.25 (d, *J* = 0.6 Hz, 3 H, CCH_3);

^{13}C{^{1}H} NMR (125 MHz, $CDCl_3$): *δ* (ppm) = 170.5 (Imi-C4), 161.4 (Imi-C2), 159.3 (Bn-C4), 147.1 (Ph-C3,5), 137.5 (Ph-C4), 136.7 (Imi-C5), 128.5 (Bn-C2,6), 128.3 (Bn-C1), 128.3 (benzylidene-C), 125.8 (Ph-C1), 114.4 (Bn-C3,5), 109.6 (Ph-C2,6), 56.5 (Ph-OCH_3), 55.5 (Bn-OCH_3), 43.5 (CH_2), 16.4 (CCH_3).

170.7 (Imi-C4), 161.4 (Imi-C2), 147.2 (Ph-C3,5), 137.5 (Ph-C4), 136.9 (Imi-C5), 136.3 (Bn-C1), 129.1 (Bn-C3,5), 128.7 (benzylidene-C), 128.0 (Bn-C4), 127.1 (Bn-C2,6), 125.9 (Ph-C1), 109.5 (Ph-C2,6), 56.5 (OCH_3), 44.0 (CH_2), 16.3 (CCH_3);

HR-MS (ESI+): *m*/*z* calc. ($C_{21}H_{23}N_2O_5$, $[M+H]^+$): 383.1601, found: 383.1612;

TLC ($CHCl_3$/EtOH 96:4 + 1% AcOH): R_f = 0.63.

1.2.34 (*Z*)-5-(4-Hydroxy-3,5-dimethoxybenzylidene)-2-methyl-3-phenyl-3,5-dihydro-4*H*-imidazol-4-one (DMHBPI, 8)

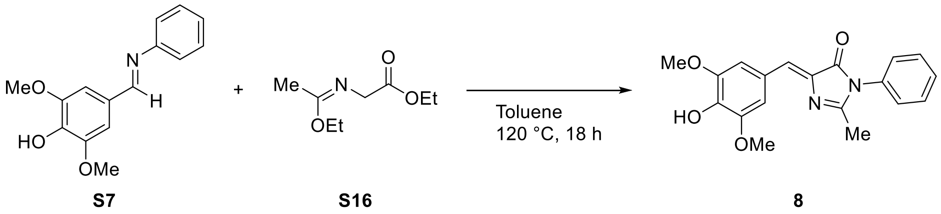

The title compound was synthesized according to General procedure C on a 1.25 mmol scale. After purification by column chromatography (Hex/EtOAc 50:50 + 1% AcOH) it was obtained as a yellow solid (100 mg, 0.30 mmol, 93%).

^{1}H NMR (400 MHz, $CDCl_3$): *δ* (ppm) = 7.56 (s, 2 H, Ph-2,6-H), 7.55–7.47 (m, 2 H, NAr-3,5-H), 7.47–7.39 (m, 1 H, NAr-4-H), 7.28–7.21 (m, 2 H, NAr-2,6-H), 7.11 (q, *J* = 0.6 Hz, 1 H, benzylidene-H), 5.95 (s, 1 H, OH), 3.96 (s, 6 H, OCH_3), 2.27 (d, *J* = 0.6 Hz, 3 H, CCH_3);

^{13}C{^{1}H} NMR (1 MHz, $CDCl_3$): *δ* (ppm) = 170.0 (Imi-C4), 160.6 (Imi-C2), 147.2 (Ph-C3,5), 137.6 (Ph-C4), 136.6 (Imi-C5), 133.8 (NAr-C1), 129.8 (NAr-C3,5), 128.9 (NAr-C4), 128.7 (benzylidene-C), 127.4 (NAr-C2,6), 125.9 (Ph-C1), 109.5 (Ph-C2,6), 56.5 (OCH_3), 16.7 (CCH_3);

HR-MS (ESI+): *m*/*z* calc. ($C_{19}H_{18}N_2NaO_4$, $[M+Na]^+$): 361.11588, found: 361.11490.

1.2.35 (*Z*)-5-(4-Hydroxy-3,5-dimethoxybenzylidene)-2-methyl-3-(4-methylphenyl)-3,5-dihydro-4*H*-imidazol-4-one (DMHBTI, 9)

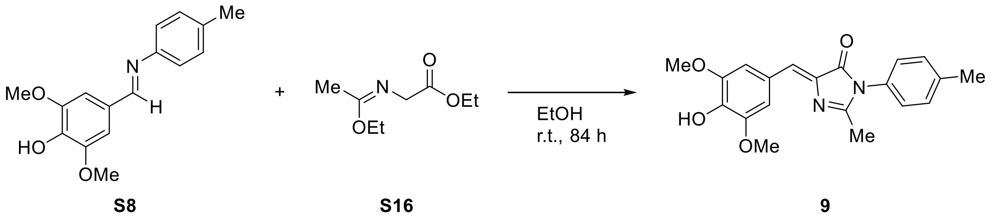

The title compound was synthesized according to General procedure C on a 1.25 mmol scale. After purification by column chromatography (Hex/EtOAc 70:30–25:75 + 1% AcOH) it was obtained as a yellow solid (237 mg, 0.67 mmol, 54%). Analytical data for a side product that was isolated during column chromatography are given below (see 1.2.40).

^{1}H NMR (500 MHz, $CDCl_3$): *δ* (ppm) = 7.56 (s, 2 H, Ph-2,6-H), 7.33–7.29 (m, 2 H, NAr-3,5-H), 7.14–7.11 (m, 2 H, NAr-2,6-H), 7.10 (q, *J* = 0.6 Hz, 1 H, benzylidene-H), 5.88 (s, 1 H, OH), 3.97 (s, 6 H, OCH_3), 2.41 (s, 3 H, NAr-CH_3), 2.25 (d, *J* = 0.6 Hz, 3 H, CCH_3);

^{13}C{^{1}H} NMR (125 MHz, $CDCl_3$): *δ* (ppm) = 170.2 (Imi-C4), 161.0 (Imi-C2), 147.2 (Ph-C3,5), 139.0 (NAr-C1), 137.5 (Ph-C4), 136.8 (Imi-C5), 131.1 (NAr-C4), 130.5 (NAr-C3,5), 128.5 (benzylidene-C), 127.3 (NAr-C2,6), 126.0 (Ph-C1), 109.5 (Ph-C2,6), 56.5 (OCH_3), 21.4 (NAr-CH_3), 16.7 (CCH_3);

HR-MS (ESI+): *m/z* calc. ($C_{20}H_{21}N_2O_4$, $[M+H]^+$): 353.1496, found: 353.1494;

TLC (Hex/EtOAc 60:40 + 1% AcOH): R_f = 0.30.

1.2.36 (*Z*)-5-(4-Hydroxy-3,5-dimethoxybenzylidene)-3-(4-methoxyphenyl)-2-methyl-3,5-dihydro-4*H*-imidazol-4-one (DMHBAI, 10)

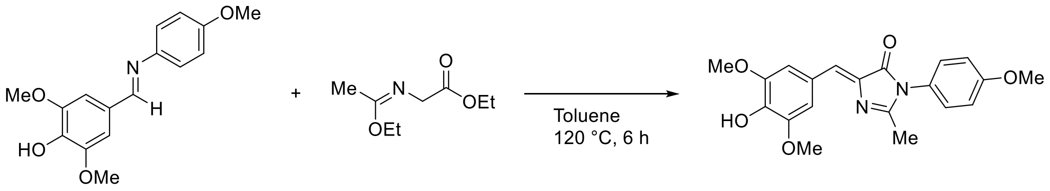

S9 **S16** **10**

The title compound was synthesized according to General procedure C on a 2.00 mmol scale. After purification by column chromatography (Hex/EtOAc 50:50–25:75 + 1% AcOH) it was obtained as a yellow solid (223 mg, 0.61 mmol, 30%).

^{1}H NMR (600 MHz, $CDCl_3$): *δ* (ppm) = 7.55 (s, 2 H, Ph-2,6-H), 7.18–7.12 (m, 2 H, NAr-2,6-H), 7.09 (s, 1 H, benzylidene-C), 7.03–6.97 (m, 2 H, NAr-3,5-H), 5.91 (s_{br}, 1 H, OH), 3.96 (s, 7 H, Ph-OCH_3), 3.85 (s, 3 H, NAr-OCH_3), 2.24 (s, 3 H, CCH_3);

^{13}C{^{1}H} NMR (125 MHz, $CDCl_3$): *δ* (ppm) = 170.2 (Imi-C4), 161.0 (Imi-C2), 159.8 (NAr-C4), 147.1 (Ph-C3,5), 137.5 (Ph-C4), 136.7 (Imi-C5), 128.6 (NAr-C2,6), 128.4 (benzylidene-C), 126.3 (NAr-C1), 126.0 (Ph-C1), 115.1 (NAr-C3,5), 109.6 (Ph-C2,6), 56.5 (Ph-OCH_3), 55.7 (NAr-OCH_3), 16.7 (CCH_3);

HR-MS (ESI+): *m/z* calc. ($C_{20}H_{21}N_2O_5$, $[M+H]^+$): 369.1445, found: 369.1453.

1.2.37 (*Z*)-5-(4-Hydroxy-3,5-dimethoxybenzylidene)-3-(4-trifluoromethylphenyl)-2-methyl-3,5-dihydro-4*H*-imidazol-4-one (DMHBTI^{F}, 11)

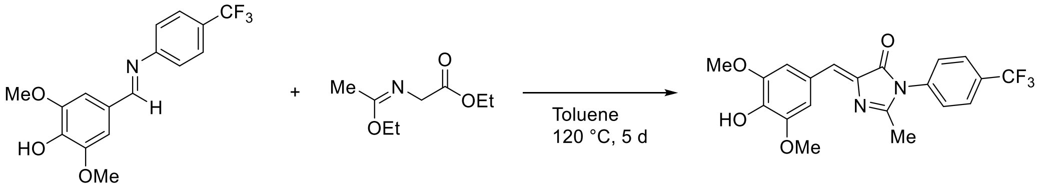

S10 **S16** **11**

The title compound was synthesized according to General procedure C on a 1.00 mmol scale. After purification by column chromatography (Hex/EtOAc 80:20–30:70 + 1% AcOH) it was obtained as a yellow solid (108 mg, 0.27 mmol, 27%).

^{1}H NMR (500 MHz, $CDCl_3$): *δ* (ppm) = 7.81–7.76 (m, 2 H, NAr-3,5-H), 7.56 (s, 2H , Ph-2,6-H), 7.43–7.39 (m, 2H, NAr-2,6-H), 7.14 (d, *J* = 0.7 Hz, 1 H, benzylidene-H), 5.92 (s, 1 H, OH), 3.97 (s, 6 H, OCH_3), 2.32 (d, *J* = 0.6 Hz, 3 H, CCH_3);

^{13}C{^{1}H} NMR (125 MHz, $CDCl_3$): *δ* (ppm) = 169.5 (Imi-C4), 159.2 (Imi-C2), 147.2 (Ph-C3,5), 137.9 (Ph-C4), 137.0 (NAr-C1), 136.1 (Imi-C5), 130.9 (q, *J* = 33.2 Hz, NAr-C4), 129.6 (benzylidene-C), 127.7 (NAr-C2,6), 127.0 (q, *J* = 3.7 Hz, NAr-C3,5), 125.7 (Ph-C1), 123.8 (q, *J* = 272.3 Hz, CF_3), 109.7 (Ph-C2,6), 56.5 (OCH_3), 16.8 (CCH_3);

^{19}F NMR (470 MHz, $CDCl_3$): *δ* (ppm) = −62.7 (CF_3);

HR-MS (ESI+): *m/z* calc. ($C_{20}H_{18}F_3N_2O_4$, $[M+H]^+$): 407.1213, found: 407.1213.

1.2.38 (*Z*)-5-(4-Hydroxy-3,5-dimethoxybenzylidene)-3-(4-trifluoromethoxyphenyl)-2-methyl-3,5-dihydro-4*H*-imidazol-4-one (DMHBAI^{F}, 12)

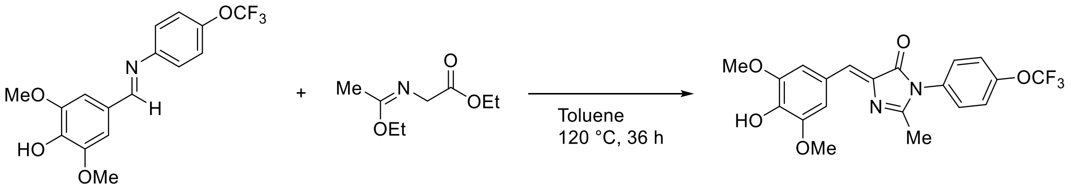

S11 **S16** **12**

The title compound was synthesized according to General procedure C on a 2.00 mmol scale. Precipitation of the product was completed by adding Et_2O (20 mL) to the reaction mixture. Orange crystalline solid (378 mg, 0.89 mmol, 45%).

^{1}H NMR (500 MHz, $CDCl_3$): *δ* (ppm) = 7.56 (s, 2 H, Ph-2,6-H), 7.38–7.34 (m, 2 H, NAr-3,5-H), 7.32–7.28 (m, 2 H, NAr-2,6-H), 7.12 (q, *J* = 0.7 Hz, 1H, benzylidene-H), 5.94 (s, 1 H, OH), 3.97 (s, 6 H, OCH_3), 2.29 (d, *J* = 0.7 Hz, 3 H, CCH_3);

^{13}C{^{1}H} NMR (125 MHz, $CDCl_3$): *δ* (ppm) = 169.7 (Imi-C4), 159.8 (Imi-C2), 149.2 (d, *J* = 2.0 Hz, NAr-C4), 147.2 (Ph-C3,5), 137.9 (Ph-C4), 136.1 (Imi-C5), 132.2 (NAr-C1), 129.3 (benzylidene-C), 128.9 (NAr-C2,6), 125.8 (Ph-C1), 122.3 (NAr-C3,5), 109.7 (Ph-C2,6), 56.5 (OCH_3), 16.7 (CCH_3);

^{19}F NMR (470 MHz, $CDCl_3$): *δ* (ppm) = −57.9 (OCF_3);

HR-MS (ESI+): *m*/*z* calc. ($C_{20}H_{18}F_3N_2O_5$, $[M+H]^+$): 423.1162, found: 423.1169.

1.2.39 (*Z*)-3-(4-*tert*-Butylphenyl)-5-(4-hydroxy-3,5-dimethoxybenzylidene)-2-methyl-3,5-dihydro-4*H*-imidazol-4-one (DMHBI^C, **15**)

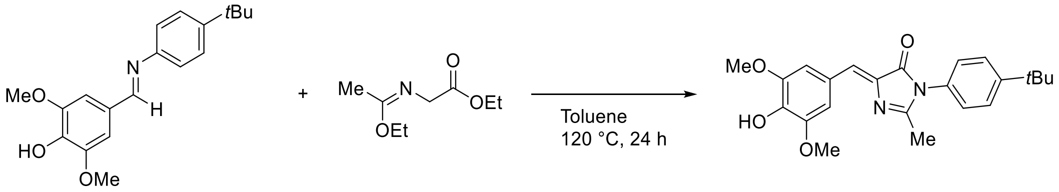

S12 **S16** **15**

The title compound was synthesized according to General procedure C on a 2.00 mmol scale. Precipitation of the product was completed by adding Et_2O (20 mL) to the reaction mixture. Dark yellow solid (604 mg, 1.53 mmol, 76%).

^{1}H NMR (500 MHz, $CDCl_3$): *δ* (ppm) = 7.56 (s, 2 H, Ph-2,6-H), 7.53–7.48 (m, 2 H, NAr-3,5-H), 7.19–7.13 (m, 2 H, NAr-2,6-H), 7.13–7.08 (m, 1 H, benzylidene-H), 5.92 (s_{br}, 1 H, OH), 3.96 (s, 6 H, OCH_3), 2.29 – 2.26 (m, 3 H, Imi-2-CH_3), 1.35 (s, 9 H, $C(CH_3)_3$);

^{13}C{^{1}H} NMR (125 MHz, $CDCl_3$): *δ* (ppm) = 170.2 (Imi-C4), 161.1 (Imi-C2), 152.0 (NAr-C4), 147.2 (Ph-C3,5), 137.5 (Ph-C4), 136.7 (Imi-C5), 131.0 (NAr-C1), 128.5 (benzylidene-C), 126.9 (Nar-C2,6), 126.8 (Nar-C3,5)', 126.0 (Ph-C1), 109.5 (Ph-C2,6), 56.5 (OCH_3), 34.9 ($\mathit{C}(CH_3)_3$), 31.4 ($C(\mathit{CH}_3)_3$), 16.8 (Imi-2-CH_3);

HR-MS (ESI+): *m*/*z* calc. ($C_{23}H_{27}N_2O_4$, $[M+H]^+$): 395.1965, found: 395.1965.

1.2.40 Methyl (*Z*)-4-(4-Hydroxy-3,5-dimethoxybenzylidene)-2-methyl-5-oxo-4,5-dihydro-1*H*-imidazol-1-yl)acetate (DMHBI-spdt, **16**)

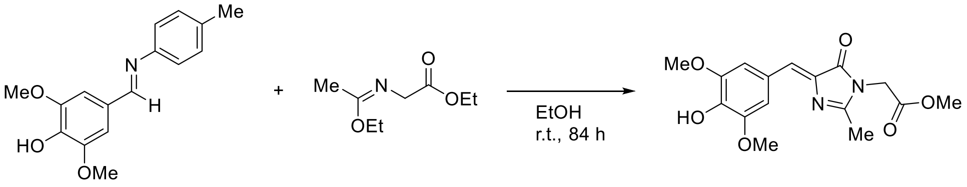

S8 **S16** **17**

The title compound was obtained as a side product during the synthesis of DMHBTI (**9**, see 1.2.35). Yellow solid (102 mg, 0.31 mmol, 24%).

^{1}H NMR (500 MHz, CD_3OD): *δ* (ppm) = 7.53 (s, 2 H, Ph-2,6-H), 7.01 (s, 1 H, benzylidene-H), 4.51 (s, 2 H, NCH_2), 3.90 (s, 6 H, Ph-3,5-OCH_3), 3.79 (s, 3 H, $COCH_3$), 2.34 (s 3 H, CCH_3);

^{13}C{^{1}H} NMR (125 MHz, CD_3OD): *δ* (ppm) = 171.7 (Imi-C5), 170.1 (*C*OCH_3), 162.5 (Imi-C2), 149.2 (Ph-C3,5), 140.3 (Ph-C4), 136.9 (Imi-C4), 130.2 (benzylidene-C), 126.2 (Ph-C1), 111.3 (Ph-C2,6), 56.8 (Ph-3,5-OCH_3), 53.2 (CO*C*H_3), 42.2 (NCH_2), 15.3 (CCH_3);

HR-MS (ESI+): *m*/*z* calc. ($C_{16}H_{18}N_2NaO_6$, $[M+Na]^+$): 357.1057, found: 357.1057;

TLC (Hex/EtOAc 60:40 + 1% AcOH): R_f = 0.09.

1.2.41 (*Z*)-5-(4-Hydroxy-3-methoxybenzylidene)-2-methyl-3-(4-methoxyphenyl)-3,5-dihydro-4*H*-imidazol-4-one (MHBAI, 17)

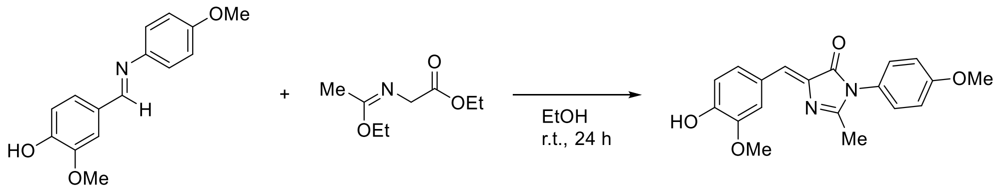

The title compound was synthesized according to General procedure C on a 1.25 mmol scale. After purification by column chromatography ($CHCl_3$/EtOH 99:1–94:6 + 1% AcOH) it was obtained as a dark yellow solid (107 mg, 316 µmol, 25%).

^{1}H NMR (400 MHz, $CDCl_3$): *δ* (ppm) = 8.04 (d, *J* = 1.9 Hz, 1H, Ph-C-2-H), 7.57 (ddd, *J* = 8.3, 1.9, 0.6 Hz, 1 H, Ph-6-H), 7.18–7.13 (m, 2 H, NAr-3,5-H), 7.13–7.12 (m, 1 H, benzylidene-H), 7.03–6.98 (m, 2 H, NAr-2,6-H), 6.96 (d, *J* = 8.3 Hz, 1 H, Ph-5-H), 6.05 (s_{br}, 1 H, OH), 3.98 (s, 3 H, Ph-OCH_3), 3.85 (s, 3 H, NAr-OCH_3), 2.24 (d, *J* = 0.6 Hz, 3 H, CCH_3);

^{13}C{^{1}H} NMR (100 MHz, $CDCl_3$): *δ* (ppm) = 170.4 (Imi-C4), 160.9 (Imi-C2), 159.9 (NAr-C1), 148.3 (Ph-C4), 146.8 (Ph-C3), 136.4 (Imi-C5), 128.7 (NAr-C3,5), 128.5 (benzylidene-C), 127.7 (Ph-C6), 127.2 (Ph-C1), 126.4 (NAr-C4), 115.1 (NAr-C2,6), 114.8 (Ph-C5), 113.9 (Ph-C2), 56.1 (Ph-OCH_3), 55.7 (NAr-OCH_3), 16.6 (CCH_3);

HR-MS (ESI+): *m/z* calc. ($C_{19}H_{18}N_2NaO_4$, $[M+Na]^+$): 361.11483, found: 361.11588.

1.2.42 (*Z*)-5-(3,5-Dimethoxybenzylidene)-3-(4-methoxyphenyl)-2-methyl-3,5-dihydro-4*H*-imidazol-4-one (DMBAI, 18)

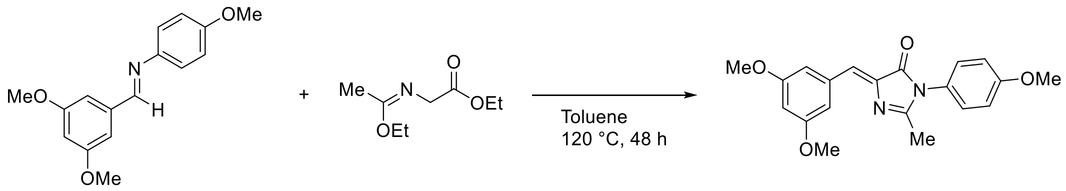

The title compound was synthesized according to General procedure C on a 2.00 mmol scale. Precipitation of the product was completed by adding pentane (20 mL) to the reaction mixture. Orange crystalline solid (484 mg, 1.37 mmol, 69%).

^{1}H NMR (300 MHz, $CDCl_3$): *δ* (ppm) = 7.40 (dd, *J* = 2.3, 0.5 Hz, 2 H, Ph-2,6-H), 7.19–7.12 (m, 2 H, NAr-3,5-H), 7.11–7.08 (m, 1 H, benzylidene-H), 7.05–6.97 (m, 2 H, NAr-2,6-H), 6.53 (t, *J* = 2.3 Hz, 1 H, Ph-4-H), 3.85 (s, 3 H, NAr-OCH_3), 3.85 (s, 6H, Ph-OCH_3), 2.24 (d, *J* = 0.7 Hz, 3 H, CCH_3);

^{13}C{^{1}H} NMR (75 MHz, $CDCl_3$): *δ* (ppm) = 170.40 (Imi-C4), 162.5 (Imi-C2), 160.9 (Ph-C3,5), 160.0 (NAr-C1), 138.8 (Imi-C5), 136.0 (Ph-C1), 128.7 (NAr-C3,5), 127.9 (benzylidene-C), 126.2 (NAr-C4), 115.1 (NAr-C2,6), 110.1 (Ph-C2,6), 103.3 (Ph-C4), 55.7 (NAr-OCH_3), 55.6 (Ph-OCH_3), 16.6 (CCH_3);

HR-MS (ESI+): *m/z* calc. ($C_{20}H_{21}N_2O_4$, $[M+H]^+$): 353.1496, found: 353.1495.

1.2.43 (*Z*)-5-(3-Bromo-4-hydroxy-5-methoxybenzylidene)-2,3-dimethyl-3,5-dihydro-4*H*-imidazol-4-one (BMHBI, 19)

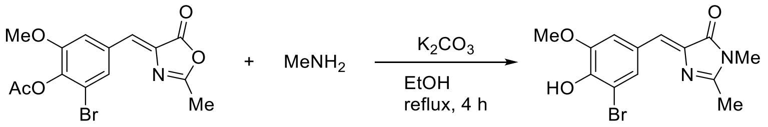

Oxazolone **S18** (300 mg, 847 µmol, 1.00 eq.), a 40% solution of $MeNH_2$ in H_2O (0.23 ml, 2.67 mmol 3.15 eq.) and K_2CO_3 (162 mg, 1.17 mmol, 1.38 eq.) were suspended in EtOH (4 ml) and heated to reflux for 4 h. After cooling to ambient temperature, the precipitate was filtered off and dissolved in aqueous acetate buffer (50 ml, pH 3.5). The solution was extracted with EtOAc (1×50 ml, 2×20 ml) and the combined organic phases were dried over Na_2SO_4. After removal of the solvent, the residue was purified by column chromatography (CH_2Cl_2/MeOH 97:3) to afford the title compound as a dark yellow solid (58 mg, 174 µmol, 21%).

^{1}H NMR (300 MHz, $CDCl_3$): *δ* (ppm) = 7.89 (d, *J* = 1.8 Hz, 1 H, Ph-6-H), 7.80 (d, *J* = 1.8 Hz, 1 H, Ph-2-H), 6.94 (q, *J* = 0.7 Hz, 1 H, benzylidene-H), 6.29 (s_{br}, 1 H, OH), 3.96 (s, 3 H, OCH_3), 3.18 (s, 3 H, NCH_3), 2.38 (d, *J* = 0.7 Hz, 3 H, CCH_3);

^{13}C{^{1}H} NMR (125 MHz, $CDCl_3$): *δ* (ppm) = 170.7 (Imi-C4), 162.2 (Imi-C2), 147.2 (Ph-C5), 145.1 (Ph-C4), 137.9 (Imi-C5), 129.8 (Ph-C2), 127.8 (Ph-C1), 126.1 (benzylidene-C), 113.2 (Ph-C6), 108.4 (Ph-C3), 56.6 (OCH_3), 26.8 (NCH_3), 15.9 (CCH_3);

HR-MS (ESI+): *m/z* calc. ($C_{13}H_{13}BrKN_2O_3$, $[M+K]^+$): 362.9741, found: 362.9750.

1.2.44 (*Z*)-5-(4-Hydroxy-3,5-dimethoxybenzylidene)-3-methyl-2-phenylethyl-3,5-dihydro-4*H*-imidazol-4-one (DMHBI-PhEt, 20)

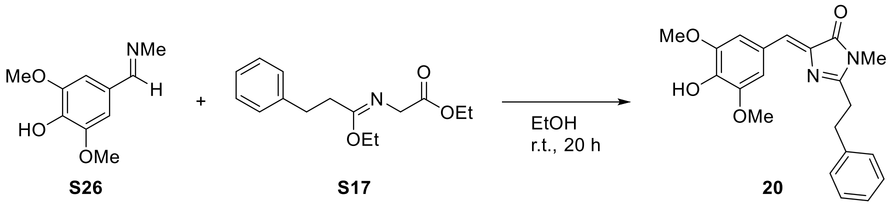

The title compound was synthesized according to General procedure C on a 1.50 mmol scale. Imine **S26** was prepared as reported previously (3). Part of the product precipitated from the reaction mixture and was filtered off. A second batch was obtained by evaporation of the filtrate and purified by column chromatography ($CHCl_3$/EtOH 98:2–90:10 + 1% AcOH). Yellow foam (399 mg, 1.09 mmol, 73%).

^{1}H NMR (600 MHz, $CDCl_3$): δ (ppm) = 7.58 (s, 2 H, Ph-2,6-H), 7.34–7.28 (m, 2 H, alkyl-Ar-3,5-H), 7.30–7.27 (m, 2 H, alkyl-Ar-2,6-H), 7.24 (m, 1 H, alkyl-Ar-4-H), 7.04 (s, 1 H, benzylidene-H), 6.00 (s_{br}, 1 H, OH), 3.92 (s, 6 H, OCH_3), 3.20 (dd, *J* = 8.5, 7.2 Hz, 2 H, CCH_2CH_2Ar), 3.08 (s, 3 H, NCH_3), 2.88 (dd, *J* = 8.5, 7.2 Hz, 2 H, CCH_2CH_2Ar);

^{13}C{^{1}H} NMR (125 MHz, $CDCl_3$): δ (ppm) = 170.8 (Imi-C4), 163.4 (Imi-C2), 147.0 (Ph-C3,5), 140.5 (alkyl-Ar-C1), 137.4 (Ph-C4), 137.0 (Imi-C5), 128.7 (alkyl-Ar-C3,5), 128.4 (alkyl-Ar-C2,6), 128.0 (benzylidene-C), 126.6 (alkyl-Ar-C4), 125.9 (Ph-C1), 109.6 (Ph-C2,6), 56.4 (OCH_3), 31.2 (CCH_2CH_2Ar), 30.8 (CCH_2CH_2Ar), 26.5 (NCH_3);

HR-MS (ESI+): *m/z* calc. ($C_{21}H_{23}N_2O_4$, $[M+H]^+$): 367.1652, found: 367.1654.

1.2.45 5-((*Z*)-4-Hydroxy-3,5-dimethoxybenzylidene)-3-methyl-2-((*E*)-2-phenylvinyl)-3,5-dihydro-4*H*-imidazol-4-one (DMHBI-Styr, 21)

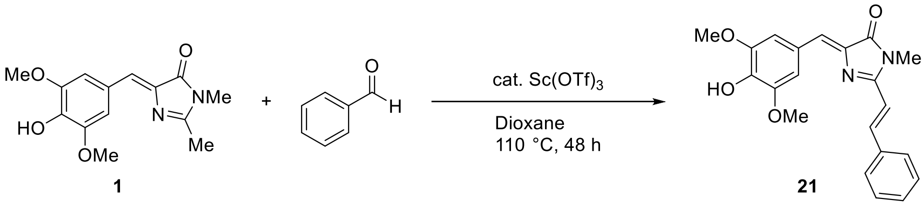

The title compound was synthesized according to General procedure D on a 1.00 mmol scale. After purification by column chromatography ($CHCl_3$/EtOH 99:1–96:4 + 1% AcOH) it was obtained as an orange-red solid (176 mg, 0.48 mmol, 48%).

^{1}H NMR (500 MHz, DMSO-d_6): δ (ppm) = 9.23 (s_{br}, 1 H, OH), 7.96 (d, *J* = 15.9 Hz, 1 H, CCHC*H*Ar), 7.84–7.79 (m, 2 H, vinyl-Ar-2,6-H), 7.76 (s, 2 H, Ph-2,6-h), 7.51–7.40 (m, 3 H, vinyl-Ar-3,5-H, vinyl-Ar-4-H), 7.25 (d, *J* = 15.9 Hz, 1 H, CC*H*CHAr), 6.99 (s, 1 H, benzylidene-H), 3.86 (s, 6 H, OCH_3), 3.28 (s, 3 H, NCH_3);

^{13}C{^{1}H} NMR (125 MHz, DMSO-d_6): δ (ppm) = 169.9 (Imi-C4), 158.7 (Imi-C2), 147.9 (Ph-C3,5), 139.5 (CCH*C*HAr), 138.7 (Ph-C4), 137.2 (Imi-C5), 135.2 (vinyl-Ar-C1), 130.1 (vinyl-Ar-C4), 129.0 (vinyl-Ar-C3,5), 128.3 (vinyl-Ar-C2,6), 126.4 (benzylidene-C), 125.0 (Ph-C1), 114.1 (C*C*HCHAr), 110.1 (Ph-C2,6), 55.9 (OCH_3), 26.4 (NCH_3);

HR-MS (ESI+): *m/z* calc. ($C_{21}H_{19}N_2O_4$, $[M-H]^-$): 363.1350, found: 363.1333.

1.2.46 5-((*Z*)-4-Hydroxy-3,5-dimethoxybenzylidene)-3-methyl-2-((*E*)-2-(pyridin-2-yl)vinyl)-3,5-dihydro-4*H*-imidazol-4-one (DMHBI-2Py, 22)

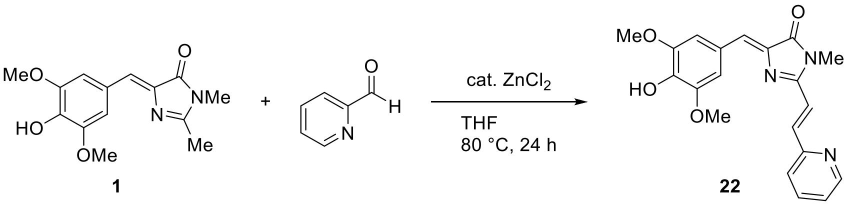

DMHBI (**1**, 207 mg, 750 µmol, 1.00 eq.), pyridine-2-carbaldehyde (90.1 mg, 841 µmol, 1.01 eq.) and anhydrous $ZnCl_2$ (10.2 mg, 75.0 µmol, 10mol%) were dissolved in THF (1.5 ml) and heated to 80 °C in a sealed tube for 16 h. Then, a second portion of the aldehyde (36.3 mg, 339 µmol, 0.45 eq.) was added and the reaction was continued under the same conditions for 8 h. The solvent was removed under reduced pressure and the residue was washed with MeOH (50 ml) to afford the title compound as an orange solid (41.4 mg, 113 µmol, 15%).

^{1}H NMR (500 MHz, DMSO-d_6): δ (ppm) = 9.23 (s_{br}, 1 H, OH), 8.68 (ddd, *J* = 4.8, 1.2, 0.9 Hz, 1 H, vinyl-Ar-6-H), 7.95 (d, *J* = 15.6 Hz, 1 H, CC*H*CHAr), 7.89 (ddd, *J* = 7.7, 7.6, 1.7 Hz, 1 H, vinyl-Ar-4-H), 7.81 (ddd, *J* = 7.7, 1.2, 0.9 Hz, 1 H, vinyl-Ar-3-H), 7.76 (s, 2 H, Ph-

2,6-H), 7.54 (d, J = 15.6 Hz, 1 H, CCHC*H*Ar), 7.41 (ddd, J = 7.6, 4.8, 1.2 Hz, 1 H, vinyl-Ar-5-H), 7.04 (s, 1 H, benzylidene-H), 3.87 (s, 6 H, OCH_3), 3.28 (s, 3 H, NCH_3);

$^{13}C\{^1H\}$ NMR (125 MHz, DMSO-d_6): δ (ppm) = 169.7 (Imi-C4), 158.2 (Imi-C2), 152.9 (vinyl-Ar-C2), 150.0 (vinyl-Ar-C6), 147.9 (Ph-C3,5), 139.0 (Ph-C4), 138.4 (C*C*HCHAr), 137.1 (vinyl-Ar-C4), 137.1 (Imi-C5), 127.3 (benzylidene-C), 124.8 (Ph-C1), 124.5 (vinyl-Ar-C3), 124.2 (vinyl-Ar-C5), 117.4 (CCH*C*HAr), 110.4 (Ph-C2,6), 56.0 (OCH_3), 26.3 (NCH_3);

HR-MS (ESI+): *m*/*z* calc. ($C_{20}H_{20}N_3O_4$, $[M+H]^+$): 366.1448, found: 366.1446.

1.2.47 5-((*Z*)-4-Hydroxy-3,5-dimethoxybenzylidene)-3-methyl-2-((*E*)-2-(pyridin-3-yl)vinyl)-3,5-dihydro-4*H*-imidazol-4-one (DMHBI-3Py, 23)

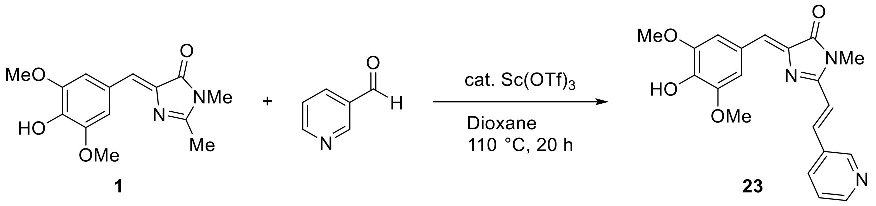

The title compound was synthesized according to General procedure D on a 200 µmol scale. Brown solid (25.3 mg, 69.2 µmol, 35%).

^1H NMR (300 MHz, DMSO-d_6): δ (ppm) = 9.27 (s_{br}, 1 H, OH), 8.97 (d, J = 2.2 Hz, 1 H, vinyl-Ar-2-H), 8.59 (dd, J = 4.8, 1.9 Hz, 1 H, vinyl-Ar-6-H), 8.30 (ddd, J = 8.0, 2.2, 1.9 Hz, 1 H, vinyl-Ar-4-H), 7.96 (d, J = 15.9 Hz, 1 H, CCHC*H*Ar), 7.76 (s, 2 H, Ph-2,6-H), 7.50 (dd, J = 8.0, 4.8 Hz, 1 H, vinyl-5-H), 7.40 (d, J = 15.9 Hz, 1 H, CC*H*CHAr), 7.02 (s, 1 H, benzylidene-C), 3.86 (s, 6 H, OCH_3), 3.29 (s, 3 H, NCH_3);

$^{13}C\{^1H\}$ NMR (125 MHz, DMSO-d_6): δ (ppm) = 169.8 (Imi-C4), 158.3 (Imi-C2), 150.4 (vinyl-Ar-C6), 149.8 (vinyl-Ar-C2), 147.9 (Ph-C3,5), 138.9 (Ph-C4), 137.0 (Imi-C5), 135.8 (CCH*C*HAr), 134.4 (vinyl-Ar-C4), 130.9 (vinyl-Ar-C3), 126.9 (benzylidene-C), 124.8 (Ph-C1), 123.8 (vinyl-Ar-C5), 116.1 (C*C*HCHAr), 110.3 (Ph-C2,6), 55.9 (OCH_3), 26.3 (NCH_3);

HR-MS (ESI+): *m*/*z* calc. ($C_{20}H_{20}N_3O_4$, $[M+H]^+$): 366.1448, found: 366.1441.

1.2.48 5-((*Z*)-4-Hydroxy-3,5-dimethoxybenzylidene)-3-methyl-2-((*E*)-2-(pyridin-4-yl)vinyl)-3,5-dihydro-4*H*-imidazol-4-one (DMHBI-4Py, 24)

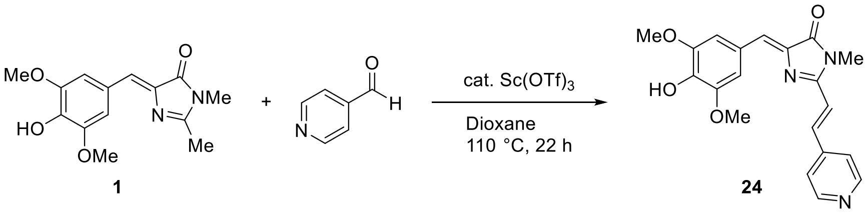

The title compound was synthesized according to General procedure D on a 250 µmol scale. After purification by column chromatography (CH_2Cl_2/MeOH 96:4–80:20) it was obtained as a dark brown solid (60.9 mg, 167 µmol, 67%).

^1H NMR (600 MHz, DMSO-d_6): δ (ppm) = 9.22 (s_{br}, 1 H, OH), 8.66 (dd, J = 4.2, 1.6 Hz, 2 H, vinyl-Ar-2,6-H), 7.88 (d, J = 15.8 Hz, 1 H, CCHC*H*Ar), 7.76 (dd, J = 4.2, 1.6 Hz, 2 H, vinyl-Ar-3,5-H), 7.75 (s, 3 H, Ph-2,6-H), 7.48 (d, J = 15.8 Hz, 1 H, CC*H*CHAr), 7.05 (s, 1 H, benzylidene-H), 3.87 (s, 6 H, OCH_3), 3.29 (s, 3 H, NCH_3);

$^{13}C\{^1H\}$ NMR (125 MHz, DMSO-d_6): δ (ppm) = 169.5 (Imi-C4), 157.8 (Imi-C2), 150.1 (vinyl-Ar-C2,6), 147.7 (Ph-C3,5), 141.9 (vinyl-Ar-C4), 139.0 (Ph-C4), 136.8 (Imi-C5), 136.2 (CCH*C*HAr), 127.5 (benzylidene-C), 124.6 (Ph-C1), 121.8 (vinyl-Ar-C3,5), 118.7 (C*C*HCHAr), 110.4 (Ph-C2,6), 55.9 (OCH_3), 26.4 (NCH_3);

HR-MS (ESI+): *m*/*z* calc. ($C_{20}H_{20}N_3O_4$, $[M+H]^+$): 366.1448, found: 366.1447.

1.2.49 2-((*E*)-2-(1*H*-Indol-3-yl)vinyl)-5-((*Z*)-4-hydroxy-3,5-dimethoxybenzylidene)-3-methyl-3,5-dihydro-4*H*-imidazol-4-one (DMHBI-Ind, 26)

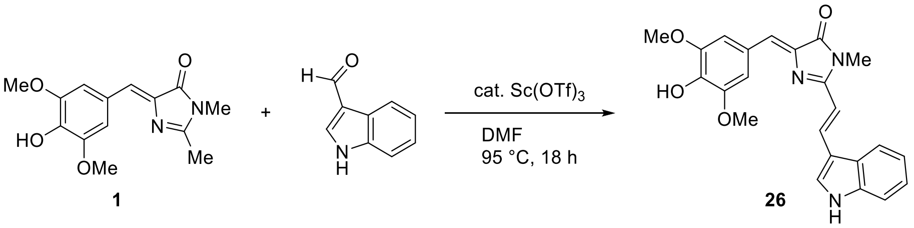

The title compound was synthesized according to General procedure D on a 200 µmol scale using DMF at 95 °C as the solvent instead of dioxane. After purification by column chromatography ($CHCl_3$/EtOH 99:1–75:25) it was obtained as an orange solid (24.4 mg, 60.5 µmol, 30%).

^{1}H NMR (500 MHz, DMSO-*d*$_6$): *δ* (ppm) = 8.28–8.22 (m, 1 H, CCHC*H*Ar), 8.04 (d, *J* = 4.5 Hz, 1 H, vinyl-Ar-2-H), 7.99 (d, *J* = 7.5 Hz, 1 H, vinyl-Ar-4-H), 7.75 (s, 2 H, Ph-2,6-H), 7.53–7.47 (m, 1 H, vinyl-Ar-7-H), 7.28–7.24 (m, 1 H, vinyl-Ar-6-H), 7.24–7.18 (m, 1 H, vinyl-Ar-5-H), 6.92–6.86 (m, 1 H, CC*H*CHAr), 6.85 (s, 1 H, benzylidene-H), 3.88 (s, 6 H, OCH_3), 3.30 (s, 3 H, NCH_3);

^{13}C{^{1}H} NMR (125 MHz, DMSO-*d*$_6$): *δ* (ppm) = 170.0 (Imi-C4), 159.6 (Imi-C2)', 147.9 (Ph-C3,5), 138.9 (Ph-C4), 137.4 (vinyl-Ar-C7a), 137.3 (Imi-C5), 134.1 (CCHC*H*Ar), 131.5 (vinyl-Ar-C2), 125.0 (vinyl-Ar-C3a), 124.9 (Ph-C1), 123.5 (benzylidene-C), 122.6 (vinyl-Ar-C6), 120.9 (vinyl-Ar-C5), 119.8 (vinyl-Ar-C4), 113.2 (vinyl-Ar-C3), 112.4 (vinyl-Ar-C7), 109.9 (Ph-C2,6), 106.7 (C*C*HCHAr), 55.9 (OCH_3), 26.1 (NCH_3);

HR-MS (ESI+): *m*/*z* calc. ($C_{23}H_{22}N_3O_4$, $[M+H]^+$): 404.1605, found: 404.1596.

1.2.50 5-((*Z*)-4-Hydroxy-3,5-dimethoxybenzylidene)-3-(4-methylphenyl)-2-((*E*)-2-(pyridin-2-yl)vinyl)-3,5-dihydro-4*H*-imidazol-4-one (DMHBTI-2Py, 27)

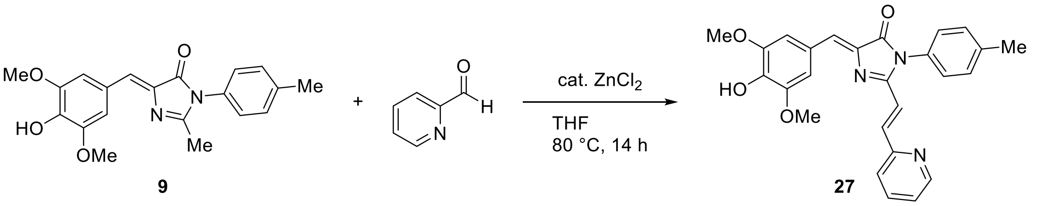

DMHBTI (**9**, 118 mg, 335 µmol, 1.00 eq.), pyridine-2-carbaldehyde (56.2 mg, 525 µmol, 1.57 eq.) and anhydrous $ZnCl_2$ (4.8 mg, 35.0 µmol, 10mol%) were dissolved in THF (1.5 ml) and heated to 80 °C in a sealed tube for 14 h. The solvent was removed under reduced pressure and the residue was purified by column chromatography (Hex/EtOAc 50:50–20:80 + 1% AcOH) to afford the title compound as an orange-red solid (135 mg, 309 µmol, 91%).

^{1}H NMR (600 MHz, $CDCl_3$): *δ* (ppm) = 8.59 (ddd, *J* = 4.8, 1.9, 0.8 Hz, 1 H), 7.90 (d, *J* = 15.5 Hz, 1 H), 7.69 (s, 1 H), 7.71–7.65 (m, 2 H), 7.36– 7.30 (m, 2 H), 7.32 (dt, *J* = 7.8, 1.0 Hz, 1 H), 7.25–7.18 (m, 5 H), 6.00 (s, 1 H), 4.02 (s, 6 H), 2.43 (s, 3 H);

^{13}C{^{1}H} NMR (125 MHz, $CDCl_3$): *δ* (ppm) = 170.1, 157.7, 153.4, 150.2, 147.2, 138.8, 137.9, 137.5, 136.8, 130.7, 130.4, 129.2, 127.4, 126.5, 124.4, 123.9, 118.3, 110.0, 56.6, 21.5;

(complete spectral assignment was not possible due to strongly overlapping ^{1}H resonances)

HR-MS (ESI+): *m*/*z* calc. ($C_{26}H_{24}N_3O_4$, $[M+H]^+$): 442.1761, found: 442.1757.

1.2.51 5-((*Z*)-4-Hydroxy-3,5-dimethoxybenzylidene)-3-(4-methylphenyl)-2-((*E*)-2-(pyridin-3-yl)vinyl)-3,5-dihydro-4*H*-imidazol-4-one (DMHBTI-3Py, 28)

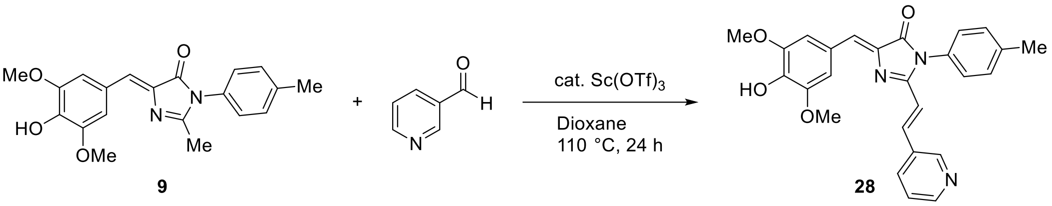

The title compound was synthesized according to General procedure D on a 200 µmol scale. After purification by column chromatography (CH_2Cl_2/MeOH 99:1–94:6 + 5% AcOH) it was obtained as an orange-brown solid (37.7 mg, 85.3 µmol, 43%).

^{1}H NMR (500 MHz, DMSO-*d*$_6$): *δ* (ppm) = 9.28 (s_{br}, 1 H, OH), 8.76 (d, *J* = 2.1 Hz, 1 H, vinyl-Ar-2-H), 8.55 (dd, *J* = 4.7, 1.8 Hz, 1 H, vinyl-Ar-6-H), 8.02 (ddd, *J* = 8.1, 2.1, 1.8 Hz, 1 H, vinyl-Ar-4-H), 7.90 (d, *J* = 16.0 Hz, 1H, CCHC*H*Ar), 7.81 (s, 2 H, Ph-2,6-H), 7.41 (dd, *J* = 8.1, 4.7 Hz, 1 H, vinyl-Ar-5-H), 7.38 (d, *J* = 8.1 Hz, 2 H, NAr-3,5-H), 7.31–7.26 (m, 2 H, NAr-2,6-H), 7.12 (s, 1 H, benzylidene-H), 6.77 (d, *J* = 16.0 Hz, 1 H, CC*H*CHAr), 3.88 (s, 6 H, OCH_3), 2.41 (s, 3 H, NAr-CH_3);

^{13}C{^{1}H} NMR (125 MHz, DMSO-*d*$_6$): *δ* (ppm) = 168.9 (Imi-C4), 156.6 (Imi-C2), 150.5 (vinyl-Ar-C6), 149.6 (vinyl-Ar-C2), 147.9 (Ph-C3,5), 139.5 (Ph-C4), 138.1 (NAr-C4), 136.0 (Imi-C5), 135.9 (CCHC*H*Ar), 134.0 (vinyl-Ar-C4), 130.7 (vinyl-Ar-C3), 130.5 (NAr-C1), 130.0 (NAr-C3,5), 128.0 (benzylidene-C), 127.4 (NAr-C2,6), 124.6 (Ph-C1), 123.9 (vinyl-Ar-C5), 116.0 (C*C*HCHAr), 110.5 (Ph-C2,6), 56.0 (OCH_3), 20.7 (NAr-CH_3);

HR-MS (ESI+): *m*/*z* calc. ($C_{26}H_{24}N_3O_4$, $[M+H]^+$): 442.1761, found: 442.1758.

1.2.52 5-((*Z*)-4-Hydroxy-3,5-dimethoxybenzylidene)-3-(4-methylphenyl)-2-((*E*)-2-(pyridin-4-yl)vinyl)-3,5-dihydro-4*H*-imidazol-4-one (DMHBTI-4Py, 29)

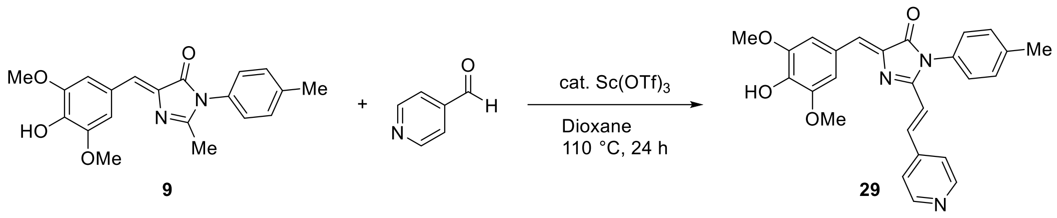

The title compound was synthesized according to General procedure D on a 200 µmol scale. After purification by column chromatography (CH_2Cl_2/MeOH 98:2 + 5% AcOH) it was obtained as a red solid (33.7 mg, 76.3 µmol, 38%).

^{1}H NMR (500 MHz, DMSO-*d*$_6$): *δ* (ppm) = 8.56 (d, *J* = 5.1 Hz, 2 H, vinyl-Ar-2,6-H), 7.76 (s, 2 H, Ph-2,6-H), 7.71 (d, *J* = 16.0 Hz, 1 H, CCHC*H*Ar), 7.49 (d, *J* = 5.1 Hz, 2 H, vinyl-Ar-3,5-H), 7.38 (m, 2 H, NAr-3,5-H), 7.28 (m, 2 H, NAr-2,6-H), 7.11 (s, 1 H, benzylidene-H), 6.86 (d, *J* = 16.0 Hz, 1 H, CC*H*CHAr), 3.85 (s, 6 H, OCH_3), 2.41 (s, 3 H, NAr-CH_3);

^{13}C{^{1}H} NMR (125 MHz, DMSO-*d*$_6$): *δ* (ppm) = 168.4 (Imi-C4), 154.0 (Imi-C2), 150.3 (vinyl-Ar-C2,6), 148.6 (Ph-C3,5), 142.1 (vinyl-Ar-C4), 137.9 (NAr-C4), 135.0 (CCHC*H*Ar), 130.8 (NAr-C1), 129.9 (NAr-C3,5), 129.2 (benzylidene-C), 128.8, 128.1, 127.4 (NAr-C2,6), 125.3, 121.5 (vinyl-Ar-C3,5), 118.7 (C*C*HCHAr), 111.1 (Ph-C2,6), 55.8 (OCH_3), 20.7 (NAr-CH_3);

HR-MS (ESI+): *m*/*z* calc. ($C_{26}H_{24}N_3O_4$, $[M+H]^+$): 442.1761, found: 442.1757.

1.2.53 2-(2-(1*H*-Imidazol-4-yl)vinyl)-5-((*Z*)-4-hydroxy-3,5-dimethoxybenzylidene)-3-(4-methylphenyl)-3,5-dihydro-4*H*-imidazol-4-one (DMHBTI-Imi, 30)

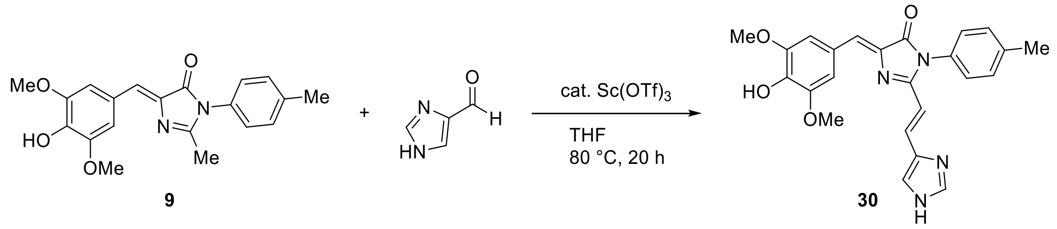

The title compound was synthesized according to General procedure D on a 150 µmol scale using THF at 80 °C as the solvent instead of dioxane. After purification by column chromatography (CH_2Cl_2/MeOH 93:7) it was obtained as an orange solid (16.9 mg, 39.2 µmol, 25%, 10:1 mixture of *E*/*Z* isomers at the newly formed C–C double bond).

^{1}H NMR (500 MHz, DMSO-*d*$_6$): *δ* (ppm) = 8.28–8.22 (m, 1 H, CCHC*H*Ar), 8.04 (d, *J* = 4.5 Hz, 1 H, vinyl-Ar-2-H), 7.99 (d, *J* = 7.5 Hz, 1 H, vinyl-Ar-4-H), 7.75 (s, 2 H, Ph-2,6-H), 7.53–7.47 (m, 1 H, vinyl-Ar-7-H), 7.28–7.24 (m, 1 H, vinyl-Ar-6-H), 7.24–7.18 (m, 1 H, vinyl-Ar-5-H), 6.92–6.86 (m, 1 H, CC*H*CHAr), 6.85 (s, 1 H, benzylidene-H), 3.88 (s, 6 H, OCH_3), 3.30 (s, 3 H, NCH_3);

^{13}C{^{1}H} NMR (125 MHz, DMSO-*d*$_6$): *δ* (ppm) = 170.0 (Imi-C4), 159.6 (Imi-C2)', 147.9 (Ph-C3,5), 138.9 (Ph-C4), 137.4 (vinyl-Ar-C7a), 137.3 (Imi-C5), 134.1 (CCHC*H*Ar), 131.5 (vinyl-Ar-C2), 125.0 (vinyl-Ar-C3a), 124.9 (Ph-C1), 123.5 (benzylidene-C), 122.6 (vinyl-Ar-C6), 120.9 (vinyl-Ar-C5), 119.8 (vinyl-Ar-C4), 113.2 (vinyl-Ar-C3), 112.4 (vinyl-Ar-C7), 109.9 (Ph-C2,6), 106.7 (C*C*HCHAr), 55.9 (OCH_3), 26.1 (NCH_3);

HR-MS (ESI+): *m*/*z* calc. ($C_{24}H_{23}N_4O_4$, $[M+H]^+$): 431.1714, found: 431.1712.

1.2.54 2-((*E*)-2-(1*H*-Indol-3-yl)vinyl)-5-((*Z*)-4-hydroxy-3,5-dimethoxybenzylidene)-3-(4-methylphenyl)-3,5-dihydro-4*H*-imidazol-4-one (DMHBTI-Ind, 31)

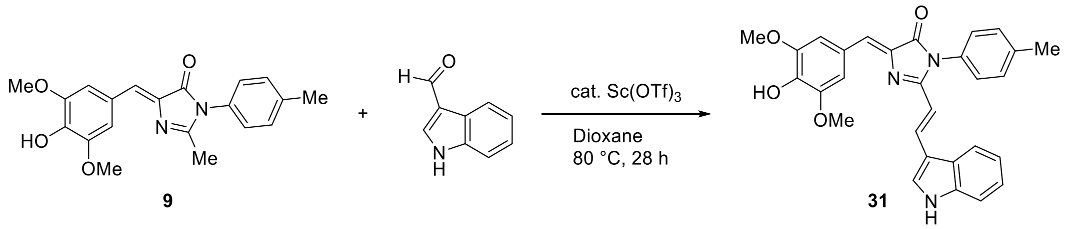

The title compound was synthesized according to General procedure D on a 150 µmol scale at 80 °C. After purification by column chromatography (CH_2Cl_2/AcOH 100:1 – CH_2Cl_2/MeOH/AcOH 10:1:1 – MeOH/AcOH 10:1) it was obtained as a brownish solid (20.3 mg, 42.3 µmol, 28%).

^{1}H NMR (500 MHz, DMSO-*d*$_6$): *δ* (ppm) = 8.14 (d, *J* = 15.7 Hz, 1 H, CCHC*H*Ar), 7.94 (s, 1 H, vinyl-Ar-2-H), 7.77 (s, 2 H, Ph-2,6-H), 7.46 (d, *J* = 8.1 Hz, 1 H, vinyl-Ar-4-H), 7.42 (m, 2 H, NAr-3,5-H), 7.38 (d, *J* = 8.1 Hz, 1 H, vinyl-Ar-7-H), 7.30 (m, 2 H, NAr-2,6-H), 7.19 (m, 1 H, vinyl-Ar-6-H), 7.09 (m, 1 H, vinyl-Ar-5-H), 6.93 (s, 1 H, benzylidene-H), 6.42 (d, *J* = 15.7 Hz, 1 H, CC*H*CHAr), 3.87 (s, 6 H, OCH_3), 2.43 (s, 3 H, NAr-CH_3);

$^{13}C\{^1H\}$ NMR (125 MHz, DMSO-*d*$_6$): *δ* (ppm) = 169.4 (Imi-C4), 157.7 (Imi-C2), 148.4 (Ph-C3,5), 138.3 (NAr-C4), 137.6 (vinyl-Ar-C7a), 134.0 (CCH*C*HAr), 131.8 (vinyl-Ar-C2), 131.2 (NAr-C1), 130.2 (NAr-C3,5), 127.8 (NAr-C2,6), 125.2 (benzylidene-C), 125.0 (vinyl-Ar-C3a), 122.9 (vinyl-Ar-C6), 121.2 (vinyl-Ar-C5), 119.1 (vinyl-Ar-C7), 113.2 (vinyl-Ar-C3), 112.9 (vinyl-Ar-C4), 110.2 (Ph-C2,6), 107.3 (C*C*HCHAr), 56.1 (OCH_3), 21.0 (NAr-CH_3);

(the ^{13}C resonances of Ph-C1, Ph-C4 and Imi-C5 were not observed)

HR-MS (ESI+): *m/z* calc. ($C_{29}H_{26}N_3O_4$, $[M+H]^+$): 480.1918, found: 480.1918.

1.2.55 2-((*E*)-2-(Ferrocenyl)vinyl)-5-((*Z*)-4-hydroxy-3,5-dimethoxybenzylidene)-3-(4-methylphenyl)-3,5-dihydro-4*H*-imidazol-4-one (DMHBI-Fc, **32**)

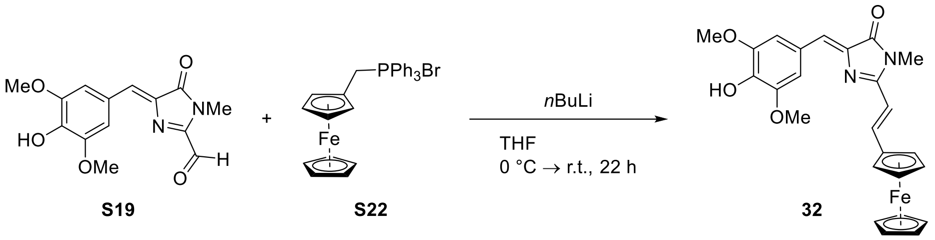

A suspension of the phosphonium salt (**S22**, 217 mg, 400 µmol, 1.00 eq.) in THF (3 ml) was cooled to 0 °C. *n*BuLi (2.5 m in hexane, 0.34 ml, 840 µmol, 2.10 eq.) was added dropwise and the resulting mixture was stirred for 30 min at the same temperature. Afterwards, the HBI derivative (**S19**, 116 mg, 400 µmol, 1.00 eq.) was added as a solid in three portions over the course of 30 min. The reaction was stirred at ambient temperature until TLC showed no further changes (22 h) and then quenched by addition of sat. aq. NH_4Cl (3 ml) and H_2O (10 ml). The mixture was extracted with CH_2Cl_2 (4×30 ml) and the combined organic phases were dried over Na_2SO_4. After removal of the solvent under reduced pressure the residue was purified by column chromatography ($CHCl_3$/EtOH 98:2–94:6 + 1% AcOH) and then filtered over silica to remove residual Ph_3PO (eluting with pentane/Et_2O followed by $CHCl_3$). The title compound was obtained as a red-brown solid (23.6 mg, 50.0 µmol, 12%).

^{1}H NMR (500 MHz, $CDCl_3$): δ (ppm) = 7.89 (d, J = 15.5 Hz, 1 H, CCHC*H*Fc), 7.64 (s, 2 H, Ph-2,6-H), 7.05 (s, 1 H, benzylidene-H), 6.39 (d, J = 15.5 Hz, 1 H, CC*H*CHFc), 5.89 (s, 1 H, OH), 4.57 (t, J = 1.9 Hz, 2 H, vinyl-Fc-2,5-H), 4.50 (t, J = 1.9 Hz, 2 H, vinyl-Fc-3,4-H), 4.20 (s, 5 H, Fc-H), 4.00 (s, 6 H, OCH3), 3.28 (s, 3 H, NCH3);

$^{13}C\{^1H\}$ NMR (125 MHz, $CDCl_3$): δ (ppm) = 171.0 (Imi-C4), 159.1 (Imi-C2), 147.2 (Ph-C3,5), 142.2 (CCH*C*HFc), 138.5 (Imi-C5), 137.2 (Ph-C4), 126.9 (Ph-C1), 126.1 (benzylidene-C), 109.7 (C*C*HCHFc), 109.5 (Ph-C2,6), 80.2 (vinyl-Fc-C1), 71.4 (vinyl-Fc-C3,4), 69.9 (Fc-C), 68.6 (vinyl-Fc-C2,5), 56.4 (OCH3), 26.8 (NCH3);

HR-MS (ESI+): m/z calc. (C25H25FeN2O4, [M+H]+): 473.1159, found: 473.1147.

1.2.56 (*Z*)-3-(4-(Dimethylamino)phenyl)-5-(4-hydroxy-3,5-dimethoxybenzylidene)-2-((*E*)-2-phenylvinyl)-3,5-dihydro-4*H*-imidazol-4-one (**33**)

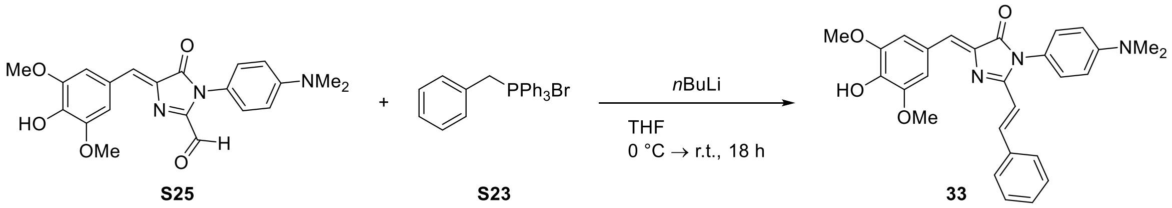

A suspension of the phosphonium salt (**S23**, 347 mg, 800 µmol, 1.00 eq.) in THF (5.4 ml) was cooled to 0 °C. *n*BuLi (2.5 M in hexane, 1.05 ml, 1.68 mmol, 2.10 eq.) was added dropwise and the resulting mixture was stirred for 30 min at the same temperature. Afterwards, the HBI derivative (**S25**, synthesized according to (3), 316 mg, 800 µmol, 1.00 eq.) was added as a solid in four portions over the course of 30 min. The reaction was stirred at ambient temperature until TLC showed no further changes (18 h) and then quenched by addition of sat. aq. NH_4Cl (10 ml) and H_2O (10 ml). The mixture was extracted with CH_2Cl_2 (4×20 ml) and the combined organic phases were dried over $MgSO_4$. After removal of the solvent under reduced pressure the residue was purified by column chromatography (CH_2Cl_2/acetone 95:5–90:10 + 1% AcOH) to afford the title compound as a brown solid (139 mg, 295 µmol, 37%).

^{1}H NMR (400 MHz, DMSO-*d*$_6$): *δ* (ppm) = 9.27 (s_{br}, 1 H, OH), 7.89 (d, *J* = 16.0 Hz, 1H, CCHC*H*Ar), 7.81 (s, 2 H, Ph-2,6-H), 7.58–7.52 (m, 2 H, vinyl-Ar-2,6-H), 7.43–7.37 (m, 3 H, vinyl-Ar-3,5-H, vinyl-Ar-4-H), 7.22–7.14 (m, 2 H, NAr-2,6-H), 7.07 (s, 1 H, benzylidene-H), 6.88–6.82 (m, 2 H, NAr-3,5-H), 6.61 (d, *J* = 16.0 Hz, 1 H, CC*H*CHAr), 3.88 (s, 6 H, OCH_3), 2.98 (s, 6 H, $N(CH_3)_2$);

$^{13}C\{^1H\}$ NMR (100 MHz, DMSO-*d*$_6$): *δ* (ppm) = 169.6 (Imi-C4), 157.9 (Imi-C2), 150.2 (NAr-C4), 147.9 (Ph-C3,5), 139.4 (CCH*C*HAr), 138.8 (Ph-C4), 136.6 (Imi-C5), 134.8 (vinyl-Ar-C1), 130.1 (vinyl-Ar-C4), 129.1 (vinyl-Ar-C3,5), 128.4 (NAr-C2,6), 127.9 (vinyl-Ar-C2,6), 127.0 (benzylidene-C), 125.0 (Ph-C1), 121.2 (NAr-C1), 114.1 (C*C*HCHAr), 112.4 (NAr-C3,5), 110.2 (Ph-C2,6), 55.9 (OCH_3), 40.1 ($N(CH_3)_2$);

HR-MS (ESI+): *m/z* calc. ($C_{28}H_{27}N_3NaO_4$, $[M+Na]^+$): 492.18938, found: 492.18866.

1.2.57 (*Z*)-5-(4-Hydroxy-3,5-dimethoxybenzylidene)-2-((*E*)-2-phenylvinyl)-3-(4-(trimethylammonium)phenyl)-3,5-dihydro-4*H*-imidazol-4-one iodide (DMHBI-Styr$^+$, **34**)

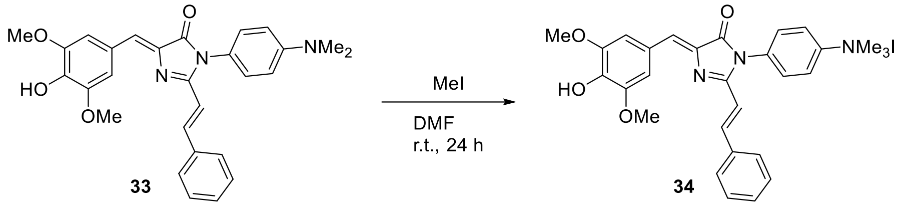

The HBI derivative (**33**, 70.4 mg, 150 µmol, 1.00 eq.) and methyl iodide (0.1 ml, 150 mmol, 1.00 eq.) were dissolved in DMF (3 mL) and stirred at ambient temperature for 24 h. Removal of the solvent under reduced pressure afforded the pure product as a brown solid (91.7 mg, 150 µmol, > 99%).

^{1}H NMR (400 MHz, DMSO-d_6): *δ* (ppm) = 9.39 (s, 1 H, OH), 8.22–8.14 (m, 2 H, NAr-3,5-H), 7.94 (d, *J* = 15.8 Hz, 1 H, CCHC*H*Ar), 7.84 (s, 2 H, Ph-2,6-H), 7.78–7.73 (m, 2 H, NAr-2,6-H), 7.63–7.58 (m, 2 H, vinyl-Ar-2,6-H), 7.46–7.39 (m, 3 H, vinyl-Ar-3,5-H, vinyl-Ar-4-H), 7.16 (s, 1 H, benzylidene-H), 6.69 (d, *J* = 15.8 Hz, 1 H, CC*H*CHAr), 3.89 (s, 6 H, OCH_3), 3.69 (s, 9 H, $N(CH_3)_3$);

^{13}C{^{1}H} NMR (100 MHz, DMSO-d_6): *δ* (ppm) = 168.7 (Imi-C4), 156.3 (Imi-C2), 147.9 (Ph-C3,5), 146.6 (NAr-C4), 139.8 (CCH*C*HAr), 139.2 (Ph-C4), 135.9 (Imi-C5), 134.8 (vinyl-Ar-C1), 134.6 (NAr-C1), 130.3 (vinyl-Ar-C4), 129.1 (vinyl-Ar-C3,5), 129.0 (NAr-C2,6), 128.1 (vinyl-Ar-C2,6), 128.0 (benzylidene-C), 124.8 (Ph-C1), 122.0 (NAr-C3,5), 113.8 (C*C*HCHAr), 110.4 (Ph-C2,6), 56.6 ($N(CH_3)_3$), 56.0 (OCH_3);

HR-MS (ESI+): *m*/*z* calc. ($C_{29}H_{30}N_3O_4$, $[M-I]^+$): 484.22308, found: 484.22400.

1.3 RNA synthesis

1.3.1 *In vitro* transcription of RNA aptamers

In vitro transcription reactions were performed with T7 RNA polymerase using the corresponding DNA template and T7 promoter strand (1 µM each) in an aqueous solution containing 40 mM Tris-HCl, pH 8.0, 30 mM $MgCl_2$, 10 mM DTT, 4 mM of each NTP and 2 mM spermidine at 37 °C for 5 h. The transcription products were purified by denaturing PAGE (15% acrylamide/bis-acrylamide 19:1, 7 M urea, 0.7×200×300 mm) with running buffer 1x TBE (89 mM Tris, 89 mM boric acid, 2 mM EDTA, pH 8.3), at 35 W constant power. The products were visualized by UV shadowing on a TLC plate and extracted by crush & soak into TEN buffer (10 mM Tris-HCl, pH 8.0, 1 mM EDTA, 300 mM NaCl) and recovered by precipitation with ethanol. Typical yields were 1–2 nmol RNA from 100 µL transcription reactions as determined by UV absorbance.

1.4 UV/Vis spectroscopy

Steady-state UV/Vis spectra were measured with a JASCO V-770 spectrophotometer equipped with a PAC-743 cell changer.

Melting curves were measured with a VARIAN CARY 100 Bio spectrophotometer equipped with a 6x6 Multicell Block Peltier Series II cell changer and a VARIAN CARY Temperature Controller

An Implen NanoPhotometer P 360 was used for RNA quantification.

Regular absorption spectra were measured in disposable semi-micro polystyrene cuvettes (10 mm path length).

Melting curves were measured in semi-micro quartz cuvettes (10 mm path length)

Stock solutions of each dye in DMSO were prepared at a concentration of 10 mM. These were diluted stepwise with DMSO to a concentration of 100 µM before being used in the preparation of analytical samples. The final DMSO concentration in all samples was < 2%.

All measurements were conducted at 25 °C unless noted otherwise.

1.4.1 Melting curves

The following samples were prepared:

- Chili RNA aptamer (2 µM) in buffer containing KCl (125 mM) and HEPES pH 7.5 (40 mM) was annealed at 95 °C for 3 min and then kept at ambient temperature for 20 min before adding DMHBI$^+$ (2 µM)
- Chili RNA aptamer (2 µM) in buffer containing KCl (125 mM) and HEPES pH 7.5 (40 mM) was annealed at 95 °C for 3 min and then kept at ambient temperature for 20 min

Inside the cuvettes, the samples were overlaid with 0.5 cm of silicon oil to minimize evaporation during the measurement. Five temperature ramps between 10 and 95 °C were collected with the following parameter settings:

- Wavelengths: 260 nm, 295 nm
- Spectral bandwidth: 1 nm
- Averaging time: 2 s
- Heating rate: 0.5 °C/min

1.5 Fluorescence spectroscopy

Steady-state fluorescence spectra were measured with a JASCO FP-8300 spectrofluorometer equipped with an FCT-817S cell changer.

Melting curves and microplate-based assays were measured with a VARIAN CARY Eclipse spectrofluorometer equipped with either a Peltier Multicell Holder Holder cell changer and a Varian CARY Temperature Controller or an Agilent Microplate Reader Accessory.

Regular emission and excitation spectra were measured in Hellma ultra-micro quartz cuvettes (1.5×1.5 mm, 3×3 mm or 10×2 mm path lengths). For kinetic assays a JASCO FMM-200 micro quartz cuvette with a magnetic stir bar (5×5 mm path length) was used.

Microplate-based assays were performed in black Corning 96 Well Half Area plates with flat bottom.

Stock solutions of each dye in DMSO were prepared at a concentration of 10 mM. These were diluted stepwise with DMSO to a concentration of 100 µM before being used in the preparation of analytical samples. The final DMSO concentration in all samples was < 2%.

All measurements were conducted at 25 °C unless noted otherwise.

1.5.1 Dye screening

The following solutions were prepared:

- RNA aptamer (1 µM) in buffer containing KCl (125 mM) and HEPES pH 7.5 (80 mM) was annealed at 95 °C for 3 min and then kept at ambient temperature for 20 min before adding $MgCl_2$ (5 mM)
- Dye (1 µM) in buffer containing KCl (125 mM), $MgCl_2$ (5 mM) and HEPES pH 7.5 (80 mM)
- Buffer containing KCl (125 mM), $MgCl_2$ (5 mM) and HEPES pH 7.5 (80 mM)

Samples were prepared by mixing the RNA and dye solutions (7.5 µL each) and incubating at ambient temperature for 3 min. For time-dependent assays, the same samples were measured again after incubating at 4 °C for 24 h. A background spectrum was obtained from a mixture of the dye and buffer solutions (7.5 µL each).

All fluorescence spectra were measured using identical parameter settings:

- Ex wavelength: maximum of the RNA aptamer–dye complex
- Em range: Ex+20–750 nm
- Ex bandwidth: 2.5 nm
- Em bandwidth: 5 nm
- Response: 50 ms
- PMT voltage: 680 V
- Data interval: 0.2 nm
- Scan speed: 500 nm/min

After background subtraction, the resulting fluorescence spectrum was integrated.

1.5.2 Mutant screening

Method A (Microplate):

The following solutions were prepared:

- RNA aptamer (0.5 µM) in buffer containing KCl (125 mM) and HEPES pH 7.5 (40 mM) was annealed at 95 °C for 3 min and then kept at ambient temperature for 20 min before adding $MgCl_2$ (5 mM) and DMHBI (2 µM)
- DMHBI (2 µM) in buffer containing KCl (125 mM), $MgCl_2$ (5 mM) and HEPES pH 7.5 (40 mM)

95 µL of each sample were transferred to a 96 well plate for measurement. A background spectrum was obtained from the DMHBI sample.

All fluorescence spectra were measured using identical parameter settings:

- Ex wavelength: 405 nm
- Em range: 450–600 nm
- Ex bandwidth: 10 nm
- Em bandwidth: 20 nm
- PMT voltage: high
- Data interval: 1.0 nm
- Scan speed: 600 nm/min

After background subtraction, the resulting fluorescence intensity at 540 nm was analyzed.

Method B (Cuvette):

Samples were prepared and measured as described for the dye screening (1.5.1).

1.5.3 Competition assay

The following samples were prepared and split into two 15 µL aliquots each:

- Chili RNA aptamer (0.5 µM) in buffer containing KCl (125 mM) and HEPES pH 7.5 (40 mM) was annealed at 95 °C for 3 min and then kept at ambient temperature for 20 min before adding $MgCl_2$ (5 mM) and DMHBAI (0.5 µm)
- Chili RNA aptamer (0.5 µM) in buffer containing KCl (125 mM) and HEPES pH 7.5 (40 mM) was annealed at 95 °C for 3 min and then kept at ambient temperature for 20 min before adding $MgCl_2$ (5 mM) and DMBAI (0.5 µm)

All samples were incubated at ambient temperature for 3 min. After measuring a first set of fluorescence spectra, one of the DMHBAI-containing samples was mixed with 15 µL of DMBAI in H_2O (10 µM) and the other one was mixed with 15 µL of H_2O. Likewise, one of the DMBAI-containing samples was mixed with 15 µL of DMHBAI in H_2O (1 µM) and the other one was mixed with 15 µL of DMHBAI in H_2O (10 µM). The samples were incubated again at ambient temperature for 3 min before the second set of fluorescence spectra was measured.

All fluorescence spectra were measured using identical parameter settings:

- Ex wavelength: 410 nm
- Em range: 430–750 nm
- Ex bandwidth: 2.5 nm
- Em bandwidth: 5 nm
- Response: 50 ms
- PMT voltage: 680 V
- Data interval: 0.2 nm
- Scan speed: 500 nm/min

1.5.4 Metal ion dependence

Samples were prepared and measured as described for the dye screening (1.5.1), using $BaCl_2$ instead $MgCl_2$ where appropriate.

1.5.5 Equilibrium binding titration

Typical aptamer, dye and buffer solutions were prepared as follows:

2x Aptamer solution:

	Added volume	Final concentration
Chili RNA aptamer (62.5 µM)	11.5 µL	16 µM
H_2O	33.5 µL	
Final volume	45 µL	

4x Buffer solution:

	Added volume	Final concentration
KCl (1 M)	250 µL	500 mM
HEPES pH 7.5 (0.5 M)	160 µL	160 mM
H_2O	90 µL	
Final volume	500 µL	

4x Dye solution:

	Added volume	Final concentration
Dye (100 µM, DMSO)	2 µL	0.4 µM
$MgCl_2$ (0.1 M)	100 µL	20 mM
H_2O	398 µL	
Final volume	500 µL	

The 2x aptamer solution was serially diluted 1:1 with H_2O to make a 15-step dilution series with a sample volume of 7.5 µL each. Next, the 4x buffer solution (3.75 µL each) was added and the samples were annealed at 95 °C for 3 min and then kept at ambient temperature for 20 min. Finally, the 4x dye solution (3.75 µL each) was added to bring the sample volume up to a total of 15 µL each. All samples were incubated at 4 °C for 16 h. A background spectrum was obtained from the 4x dye and 4x buffer solutions in H_2O.

As described above, the samples contained 0.0005–8 µM RNA and 0.1 µM dye. Samples with different concentrations (see Supplementary Figure **6**.) were prepared analogously.

Fluorescence spectra were measured using the following parameters:

- Ex wavelength: maximum of the RNA aptamer–dye complex
- Em range: Ex+20–750 nm
- Ex bandwidth: 2.5 nm
- Em bandwidth: 5 nm

- Response: 1 s
- PMT voltage: adjusted for optimal signal intensity at the highest RNA concentration
- Data interval: 0.2 nm
- Scan speed: 500 nm/min

After background subtraction, the resulting fluorescence spectra were integrated. The data points were fitted with the following expression describing one-site binding with ligand depletion:

$$I = \frac{x}{2}\left[\left(c_{\text{dye, initial}} + c_{\text{RNA, initial}} + K_{\text{d}}\right) - \sqrt{\left(c_{\text{dye, initial}} + c_{\text{RNA, initial}} + K_{\text{d}}\right)^2 - 4 \cdot c_{\text{dye, initial}} \cdot c_{\text{RNA, initial}}}\right] \quad (1)$$

If the data quality did not warrant fitting with this model, the Hill equation was used instead.

1.5.6 Association kinetics

The following solutions were prepared:

- Chili RNA aptamer (26.25 nM) in buffer containing KCl (131.25 mM) and HEPES pH 7.5 (42 mM) was annealed at 95 °C for 3 min and then kept at ambient temperature for 20 min before adding $MgCl_2$ (5.5 mM)
- Dye (15.75, 21, 31.5 and 42 µM) in H_2O

For each concentration, the dye solution (20 µL) was quickly injected into the RNA solution (400 µL) and the fluorescence intensity was monitored for up to 30 min while stirring the mixture.

- Fluorescence time courses of each sample were measured using identical parameter settings:
- Ex wavelength: maximum of the RNA aptamer–dye complex
- Em wavelength: maximum of the RNA aptamer–dye complex
- Ex bandwidth: 1 nm
- Em bandwidth: 20 nm
- Response: 50 ms
- PMT voltage: adjusted for optimal signal intensity
- Data interval: 2 s

The data points were fitted with a biexponential association model to obtain the apparent rate constants k_{obs}. Plots of k_{obs} against the dye concentration were fitted with a linear equation to obtain the respective association rates k_{on}.

1.5.7 Melting curves

A UV/Vis melting sample containing both Chili and $DMHBI^+$ (1.4.1) was reused to collect five temperature ramps between 10 and 95 °C with the following parameter settings:

- Ex wavelength: 413 nm
- Em wavelength: 542 nm
- Ex bandwidth: 5 nm
- Em bandwidth: 5 nm
- Averaging time: 100 ms
- PMT voltage: 800 V
- Heating rate: 0.5 °C/min

1.6 NMR spectroscopy

All NMR experiments with oligonucleotides were performed on a Bruker Avance III 600 NMR spectrometer equipped with a DCH ^{13}C / ^{1}H cryoprobe. The NMR spectra were acquired and processed using the software Topspin 3.2 (Bruker BioSpin, Germany). The suppression of the water signal was achieved using the jump-return-Echo scheme (10). All NMR samples were referenced using 3-(trimethylsilyl)-1-propanesulfonic acid (DSS) and dissolved in 10% D_2O / 90% H_2O containing either 25 mM Tris buffer (pH 7.4) or 25 mM KP_i buffer (pH 7.4). The ligands were added to the NMR samples directly in the NMR tube from a 10 mM stock solution in DMSO-d_6 (final concentration of DMSO-d_6 in the NMR sample < 2%). Measurements were conducted at 25 °C unless noted otherwise.

1.6.1 H_2O/D_2O exchange

The D_2O exchange experiment was performed as follows: 298 µL of 9.4% D_2O / 89.3% H_2O / 1.3% DMSO-d_6 containing Chili RNA (130 mM), $DMHBI^+$ (1.00 eq.), KCl (50 mM), $MgCl_2$ (1 mM) and Tris buffer pH 7.4 (25 mM) were frozen in liquid nitrogen and lyophilized to dryness. The sample was redissolved in 298 µL of 98.7% D_2O / 1.3% DMSO-d_6 immediately before acquiring the spectra.

1.7 Isothermal titration calorimetry

A stock solution of the Chili RNA aptamer (150 µL) was dialyzed against ultrapure H_2O using a Slide-A-Lyzer MINI device (3.5K MWCO, 0.5 mL, ThermoFisher Scientific) according to the manufacturer's instructions. Typical aptamer, dye and buffer solutions were prepared as follows:

Aptamer solution:

	Added volume	Final concentration
Chili RNA aptamer (150 µM, dialyzed)	150 µL	15 µM
KCl (1 M)	187.5 µL	125 mM
HEPES pH 7.5 (0.5 M)	120 µL	40 mM
DMSO	22.5 µL	1.5%
Anneal 3 min at 95 °C		
Incubate 20 min at 25 °C		
$MgCl_2$ (0.1 M)	75 µL	5 mM
H_2O	945 µL	
Final volume	1500 µL	

Dye solution:

	Added volume	Final concentration
H_2O	150 µL	
KCl (1 M)	187.5 µL	125 mM
HEPES pH 7.5 (0.5 M)	120 µL	40 mM
Dye (10 mM, DMSO)	22.5 µL	150 µM
Heat 3 min at 95 °C		
Incubate 20 min at 25 °C		
$MgCl_2$ (0.1 M)	75 µL	5 mM
H_2O	945 µL	
Final volume	1500 µL	

Buffer solution:

	Added volume	Final concentration
H_2O	150 µL	
KCl (1 M)	187.5 µL	125 mM
HEPES pH 7.5 (0.5 M)	120 µL	40 mM
DMSO	22.5 µL	1.5%
Heat 3 min at 95 °C		
Incubate 20 min at 25 °C		
$MgCl_2$ (0.1 M)	75 µL	5 mM
H_2O	945 µL	
Final volume	1500 µL	

For $DMHBI^+$, the final concentrations of RNA and dye were reduced to 10 and 100 µM, respectively. All measurements used the following parameter settings:

- Volume of aptamer solution in the cell: 280 µL
- Volume of dye solution in the syringe: 40 µL
- Temperature: 25.0 °C
- Reference power: 41.9 µW
- Feedback: High
- Stir speed: 750 rpm
- Initial delay: 60 s
- First injection: 0.4 µL over 0.8 s
- Other injections: 12 × 3.0 µL over 6.0 s (DMHBI) or 18 × 2.0 µL over 4.0 s ($DMHBI^+$)
- Spacing: 150 s

A baseline correction was performed by subtracting the mean injection heat of dye into buffer from the titration data. The data points were fitted with a model describing a set of identical binding sites as implemented in the device software. Initially, the number of binding sites was constrained to 1 and the active concentration of RNA in the cell was varied.

2 Computational methods

DFT-optimized geometries were calculated with the software package ORCA version 4.0.1.2 (11,12) using the B3LYP functional with D3BJ dispersion correction (13,14), a def2-TZVP basis set (15,16) and the corresponding auxiliary basis set for the RIJCOSX approximation (17) on all light atoms. For iodine, the augmented ma-def2-TZVP basis set was used together with the default ECP (18). Stationary points were characterized as minima on the potential energy surface by analytical frequency calculations. Tight convergence criteria were used throughout.

2.1 Typical ORCA input file

```
#  !B3LYP D3BJ def2-TZVP RIJCOSX def2/J Grid5 FinalGrid6 GridX6 TightSCF TightOpt Freq
#
#  %basis
#         newgto I "ma-def2-TZVP" end
#  end
#
#  *xyzfile 0 1 dmhb_p-trimethylammoniumphenyl_i_iodide_start.xyz
```

3 Supporting Tables

Supplementary Table 1. Calculated dipole moments of HBI derivatives in the gas phase (B3LYP-D3/def2-TZVP).

Compound	$\|\mu\|$<br>D
DMHBI-*i*Pr (**3**)	1.66302
DMHBI-MeCy (**5**)	1.62409
DMHBI-Bn (**6**)	1.49230
DMHBTI (**9**)	1.7779
DMHBAI (**10**)	2.31785
DMHBTI^F (**11**)	3.16969
DMHBAI^F (**12**)	2.64354
DMHBI$^+$ (**14**)	16.71988
DMHBI^C (**15**)	1.88552

Supplementary Table 2. Excitation and emission wavelengths for selected HBI derivatives in aqueous solution.

Compound	λ_{Ex}<br>nm	λ_{Em}<br>nm
DMHBI (**1**)[c]	378,479	485,537
DMHBI-Et (**2**)	391,478	484,533
DMHBI-*i*Pr (**3**)	386,476	484,534
DMHBI-*t*Bu (**4**)	382,477	486,535
DMHBI-MeCy (**5**)	389,473	486,533
DMHBPI (**8**)	389,479	487,536
DMHBTI (**9**)	392,477	488,536
DMHBAI (**10**)	396,478	487,538
DMHBTI^F (**11**)	389,481	489,536
DMHBAI^F (**12**)	378,479	488,538
DMHBI-DMA (**13**)	477	487,535
DMHBI$^+$ (**14**)[a]	379,493	486,540
DMHBI^C (**15**)	478	486,533

[a] Reported previously in (3).

Supplementary Table 3. Fluorescence intensity of Chili mutant–DMHBI complexes (Ex/Em 405/540 nm) from the microplate-based screening assay.

Chili mutant	I_{540}
Wt Chili RNA aptamer	200.2
G9A	38.5
G10A	41.5
A11U	40.9
G12A	46.2
G13A	37.8
G14A	65.2
G15A	47.2
C16U	32.2
G31A	44.2
G32A	48.8
U33C	46.9
U34C	203.1
G35A	138.2
G36A	192.9
G37A	53.5
U38C	129.3
G39A	44.5
C40U	156.1
G41A	43.4
G42A	41.2
U43C	53.6
C44U	122.6

Supplementary Table 4. DNA and RNA sequences.

Description	5'-Sequence-3'	nt
**RNA**		
wt Chili RNA aptamer	GGCUAGCUGGAGGGGCGCCAGUUCGCUGGUGGUUGGGUGCGGUCGGCUAGCC	52
Chili bottom stem loop	GGCUAGCUG············UUCG··················CGGCUAGCC	22
Chili top stem loop	CGCCAGUUCGCUGGUG	16
Chili mutants:		
G9A	GGCUAGCUAGAGGGGCGCCAGUUCGCUGGUGGUUGGGUGCGGUCGGCUAGCC	52
G10A	GGCUAGCUGAAGGGGCGCCAGUUCGCUGGUGGUUGGGUGCGGUCGGCUAGCC	52
A11U	GGCUAGCUGGUGGGGCGCCAGUUCGCUGGUGGUUGGGUGCGGUCGGCUAGCC	52
G12A	GGCUAGCUGGAAGGGCGCCAGUUCGCUGGUGGUUGGGUGCGGUCGGCUAGCC	52
G13A	GGCUAGCUGGAGAGGCGCCAGUUCGCUGGUGGUUGGGUGCGGUCGGCUAGCC	52
G14A	GGCUAGCUGGAGGAGCGCCAGUUCGCUGGUGGUUGGGUGCGGUCGGCUAGCC	52
G15A	GGCUAGCUGGAGGGACGCCAGUUCGCUGGUGGUUGGGUGCGGUCGGCUAGCC	52
C16U	GGCUAGCUGGAGGGGUGCCAGUUCGCUGGUGGUUGGGUGCGGUCGGCUAGCC	52
G31A	GGCUAGCUGGAGGGGCGCCAGUUCGCUGGUAGUUGGGUGCGGUCGGCUAGCC	52
G32A	GGCUAGCUGGAGGGGCGCCAGUUCGCUGGUGAUUGGGUGCGGUCGGCUAGCC	52
U33C	GGCUAGCUGGAGGGGCGCCAGUUCGCUGGUGGCUGGGUGCGGUCGGCUAGCC	52
U34C	GGCUAGCUGGAGGGGCGCCAGUUCGCUGGUGGUCGGGUGCGGUCGGCUAGCC	52
G35A	GGCUAGCUGGAGGGGCGCCAGUUCGCUGGUGGUUAGGUGCGGUCGGCUAGCC	52
G36A	GGCUAGCUGGAGGGGCGCCAGUUCGCUGGUGGUUGAGUGCGGUCGGCUAGCC	52
G37A	GGCUAGCUGGAGGGGCGCCAGUUCGCUGGUGGUUGGAUGCGGUCGGCUAGCC	52
U38C	GGCUAGCUGGAGGGGCGCCAGUUCGCUGGUGGUUGGGCGCGGUCGGCUAGCC	52
G39A	GGCUAGCUGGAGGGGCGCCAGUUCGCUGGUGGUUGGGUACGGUCGGCUAGCC	52
C40U	GGCUAGCUGGAGGGGCGCCAGUUCGCUGGUGGUUGGGUGUGGUCGGCUAGCC	52
G41A	GGCUAGCUGGAGGGGCGCCAGUUCGCUGGUGGUUGGGUGCAGUCGGCUAGCC	52
G42A	GGCUAGCUGGAGGGGCGCCAGUUCGCUGGUGGUUGGGUGCGAUCGGCUAGCC	52
U43C	GGCUAGCUGGAGGGGCGCCAGUUCGCUGGUGGUUGGGUGCGGCCGGCUAGCC	52
C44U	GGCUAGCUGGAGGGGCGCCAGUUCGCUGGUGGUUGGGUGCGGUUGGCUAGCC	52
G9A/C44U	GGCUAGCUAGAGGGGCGCCAGUUCGCUGGUGGUUGGGUGCGGUUGGCUAGCC	52
C16U/G31C	GGCUAGCUGGAGGGGUGCCAGUUCGCUGGUAGUUGGGUGCGGUCGGCUAGCC	52
**DNA**		
T7 promotor	CTGTAATACGACTCACTATA	20
Txn template for wt Chili	GGCTAGCCGACCGCACCCAACCACCAGCGAACTGGCGCCCCTCCAGCTAGCCTATAGTGAGTCGTATTACAG	72
Txn templates for Chili mutants:		
G9A	GGCTAGCCGACCGCACCCAACCACCAGCGAACTGGCGCCCCTCTAGCTAGCCTATAGTGAGTCGTATTACAG	72
G10A	GGCTAGCCGACCGCACCCAACCACCAGCGAACTGGCGCCCCTTCAGCTAGCCTATAGTGAGTCGTATTACAG	72
A11U	GGCTAGCCGACCGCACCCAACCACCAGCGAACTGGCGCCCCACCAGCTAGCCTATAGTGAGTCGTATTACAG	72
G12A	GGCTAGCCGACCGCACCCAACCACCAGCGAACTGGCGCCCTTCCAGCTAGCCTATAGTGAGTCGTATTACAG	72
G13A	GGCTAGCCGACCGCACCCAACCACCAGCGAACTGGCGCCTCTCCAGCTAGCCTATAGTGAGTCGTATTACAG	72
G14A	GGCTAGCCGACCGCACCCAACCACCAGCGAACTGGCGCTCCTCCAGCTAGCCTATAGTGAGTCGTATTACAG	72
G15A	GGCTAGCCGACCGCACCCAACCACCAGCGAACTGGCGTCCCTCCAGCTAGCCTATAGTGAGTCGTATTACAG	72
C16U	GGCTAGCCGACCGCACCCAACCACCAGCGAACTGGCACCCCTCCAGCTAGCCTATAGTGAGTCGTATTACAG	72
G31A	GGCTAGCCGACCGCACCCAACTACCAGCGAACTGGCGCCCCTCCAGCTAGCCTATAGTGAGTCGTATTACAG	72
G32A	GGCTAGCCGACCGCACCCAATCACCAGCGAACTGGCGCCCCTCCAGCTAGCCTATAGTGAGTCGTATTACAG	72
U33C	GGCTAGCCGACCGCACCCAGCCACCAGCGAACTGGCGCCCCTCCAGCTAGCCTATAGTGAGTCGTATTACAG	72
U34C	GGCTAGCCGACCGCACCCGACCACCAGCGAACTGGCGCCCCTCCAGCTAGCCTATAGTGAGTCGTATTACAG	72
G35A	GGCTAGCCGACCGCACCTAACCACCAGCGAACTGGCGCCCCTCCAGCTAGCCTATAGTGAGTCGTATTACAG	72
G36A	GGCTAGCCGACCGCACTCAACCACCAGCGAACTGGCGCCCCTCCAGCTAGCCTATAGTGAGTCGTATTACAG	72
G37A	GGCTAGCCGACCGCATCCAACCACCAGCGAACTGGCGCCCCTCCAGCTAGCCTATAGTGAGTCGTATTACAG	72
U38C	GGCTAGCCGACCGCGCCCAACCACCAGCGAACTGGCGCCCCTCCAGCTAGCCTATAGTGAGTCGTATTACAG	72
G39A	GGCTAGCCGACCGTACCCAACCACCAGCGAACTGGCGCCCCTCCAGCTAGCCTATAGTGAGTCGTATTACAG	72
C40U	GGCTAGCCGACCACACCCAACCACCAGCGAACTGGCGCCCCTCCAGCTAGCCTATAGTGAGTCGTATTACAG	72
G41A	GGCTAGCCGACTGCACCCAACCACCAGCGAACTGGCGCCCCTCCAGCTAGCCTATAGTGAGTCGTATTACAG	72
G42A	GGCTAGCCGATCGCACCCAACCACCAGCGAACTGGCGCCCCTCCAGCTAGCCTATAGTGAGTCGTATTACAG	72
U43C	GGCTAGCCGGCCGCACCCAACCACCAGCGAACTGGCGCCCCTCCAGCTAGCCTATAGTGAGTCGTATTACAG	72
C44U	GGCTAGCCAACCGCACCCAACCACCAGCGAACTGGCGCCCCTCCAGCTAGCCTATAGTGAGTCGTATTACAG	72
G9A/C44U	GGCTAGCCAACCGCACCCAACCACCAGCGAACTGGCGCCCCTCTAGCTAGCCTATAGTGAGTCGTATTACAG	72
C16U/G31A	GGCTAGCCGACCGCACCCAACTACCAGCGAACTGGCACCCCTCCAGCTAGCCTATAGTGAGTCGTATTACAG	72

4 Supporting Figures

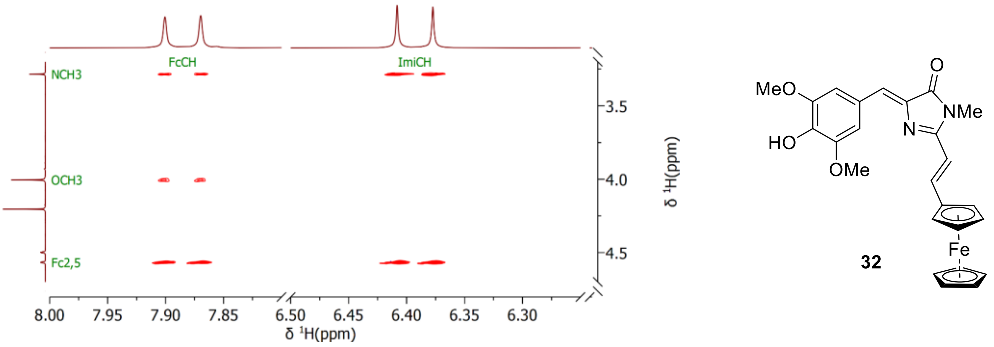

Supplementary Figure 1. ^{1}H-^{1}H NOESY NMR spectrum of DMHBI-Fc (**32**). Correlations between the NCH_3 group, the OCH_3 group and the respective positions of the double bond at C2 suggest a predominant *s*-cis configuration as shown above.

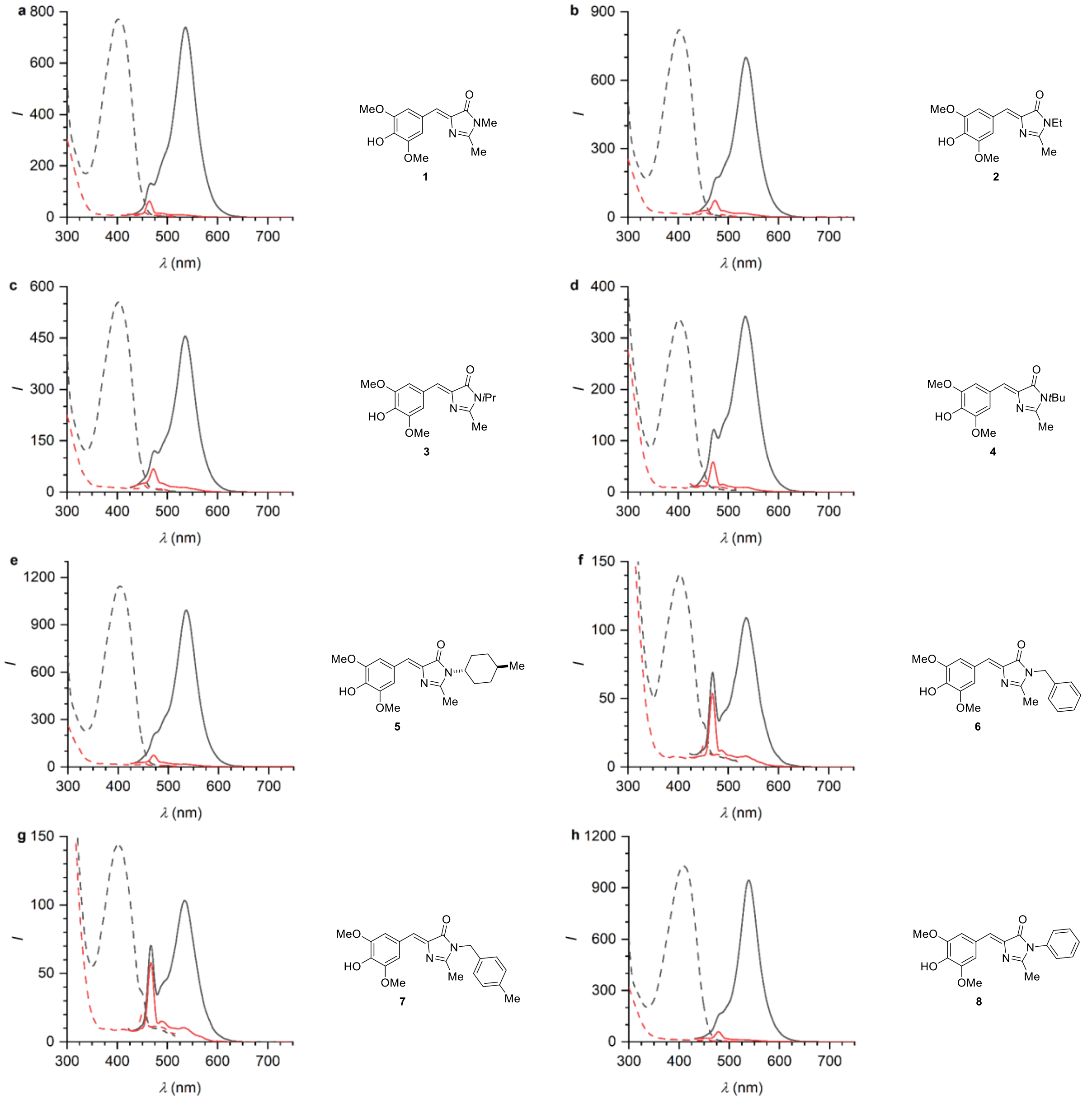

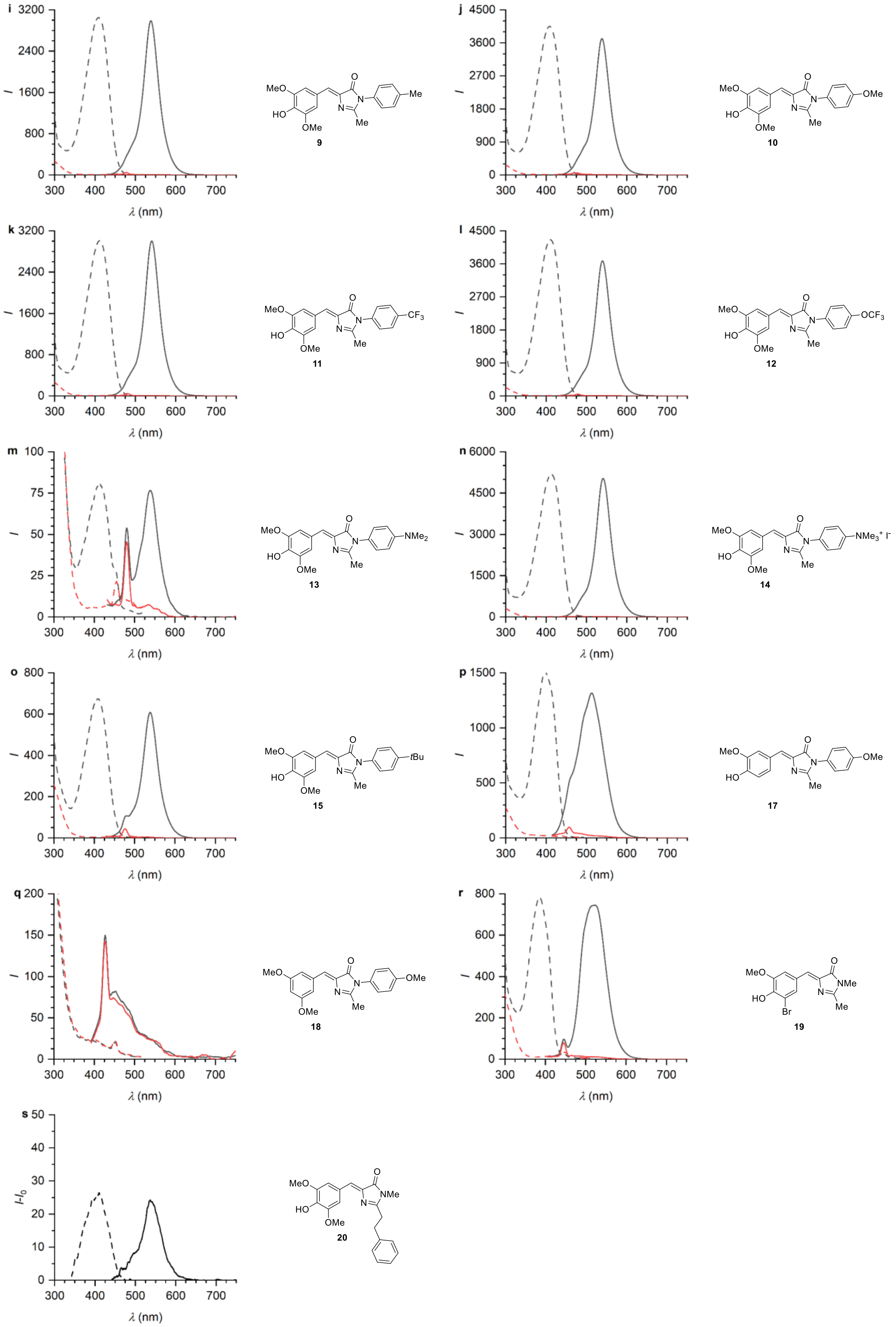

Supplementary Figure 2. Uncorrected fluorescence emission (solid) and excitation (dashed) spectra of Chili–HBI complexes with green fluorescence (black) and of the respective HBI ligands alone (red). The excitation and emission wavelengths used to obtain the spectra are given in parentheses. a) DMHBI (**1**, 400/537 nm), b) DMHBI-Et (**2**, 400/537 nm), c) DMHBI-*i*Pr (**3**, 400/537 nm), d) DMHBI-

*t*Bu (**4**, 400/534 nm), e) DMHBI-MeCy (**5**, 400/537 nm), f) DMHBI-Bn (**6**, 400/537 nm), g) DMHBI-PMBn (**7**, 400/535 nm), h) DMHBPI (**8**, 410/539 nm), i) DMHBTI (**9**, 410/539 nm), j) DMHBAI (**10**, 410/538 nm), k) DMHBTI^F (**11**, 413/540 nm), l) DMHBAI^F (**12**, 413/540 nm), m) DMHBI-DMA (**13**, 413/540 nm), n) DMHBI$^+$ (**14**, 413/542 nm), o) DMHBI^C (**15**, 410/539 nm), p) MHBAI (**17**, 395/513 nm), q) DMBAI (**18**, 372/535 nm, no signal was obtained at these or any other wavelengths), r) BMHBI (**19**, 386/520 nm), s) DMHBI-PhEt (**20**, 400/539 nm).

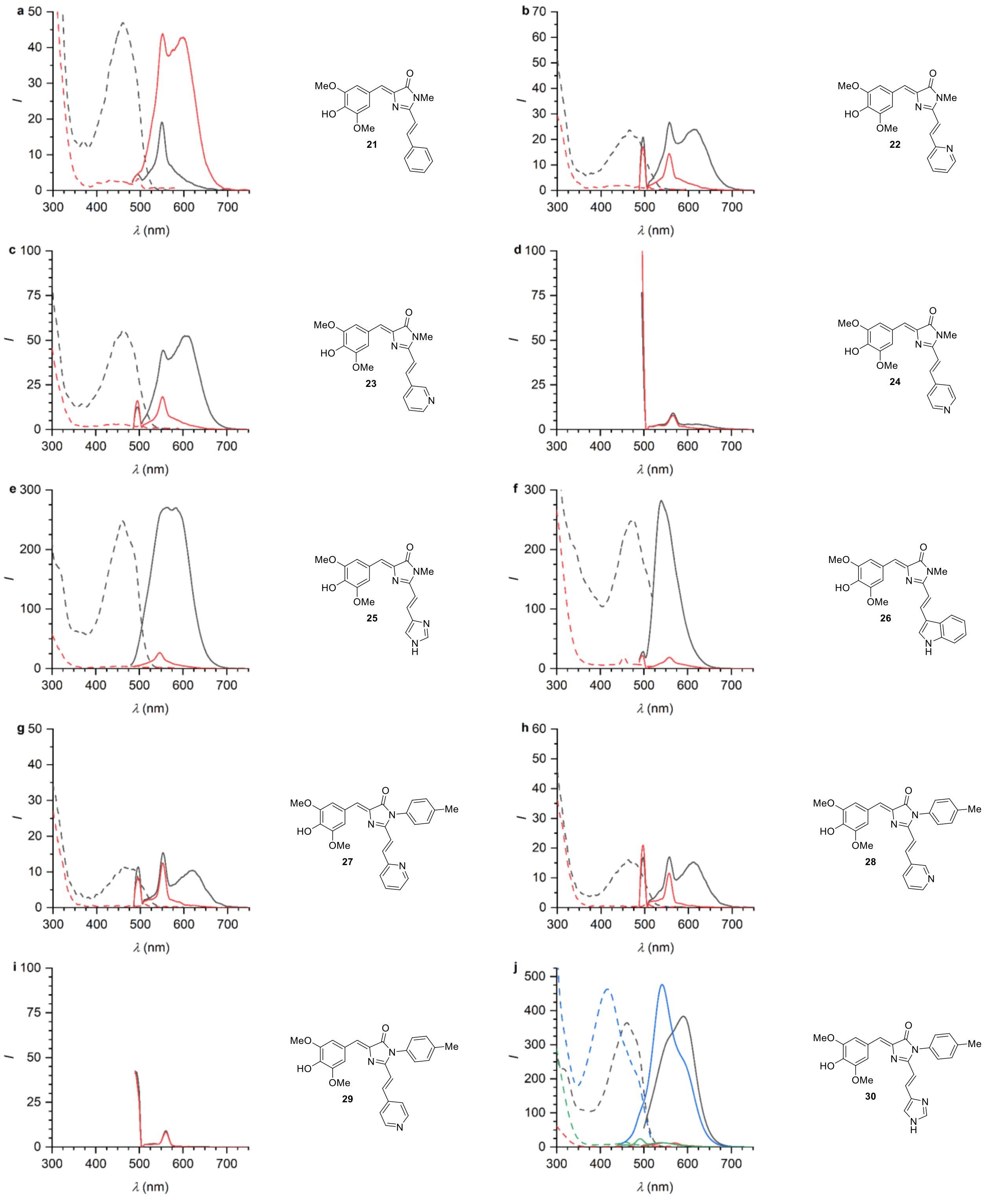

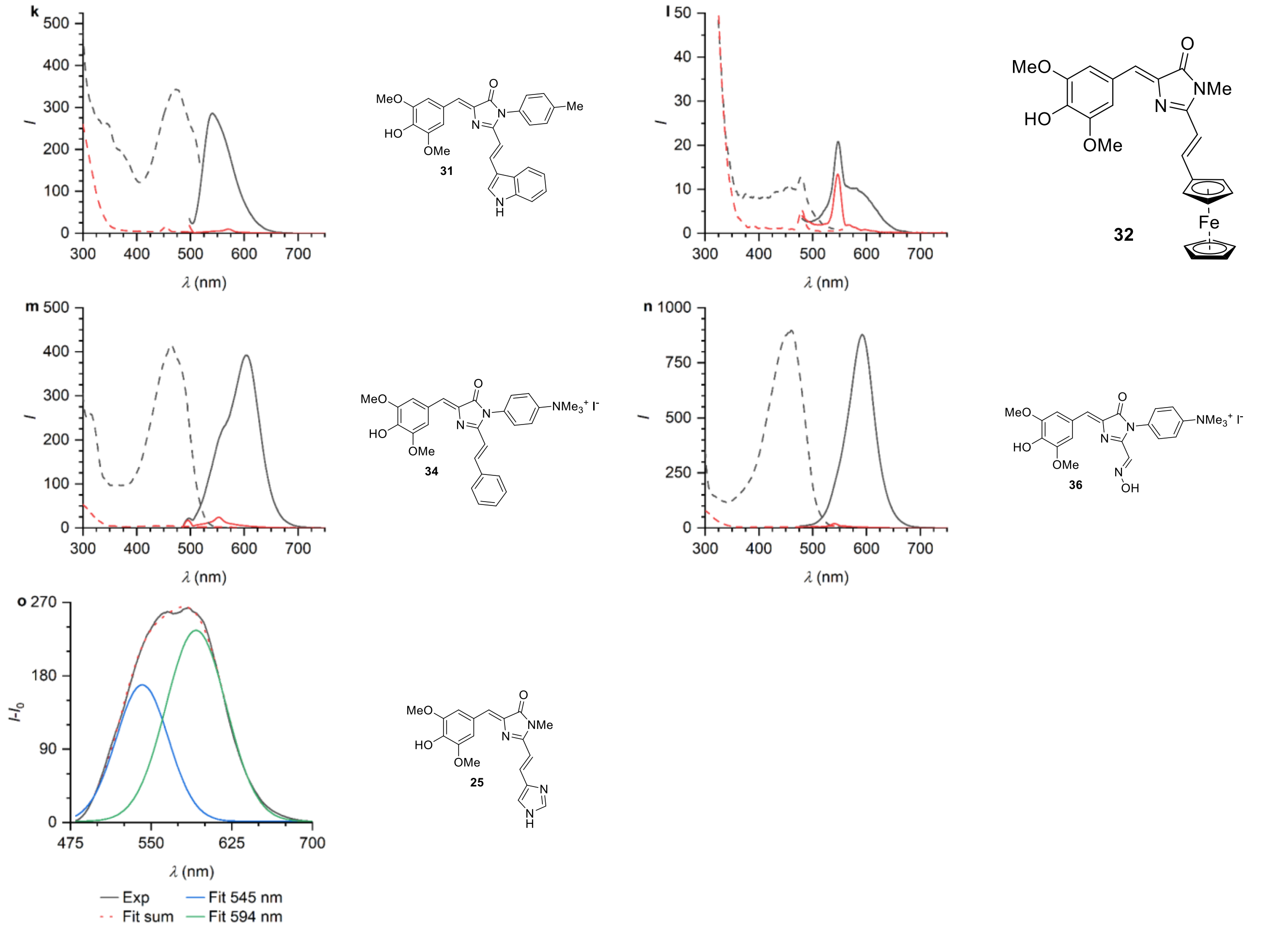

Supplementary Figure 3. Uncorrected fluorescence emission (solid) and excitation (dashed) spectra of the Chili–HBI complexes with π-conjugated C2 substituents, i.e. with red fluorescence (black), and of the respective HBI ligands alone (red). The respective excitation and emission wavelengths used to obtain the spectra are given in parentheses. a) DMHBI-Styr (**21**, 462/601 nm), b) DMHBI-2Py (**22**, 467/616 nm), c) DMHBI-3Py (**23**, 465/611 nm), d) DMHBI-4Py (**24**, 475/–, no signal was obtained at this or any other wavelength), e) DMHBI-Imi, (**25**, 463/594 nm), f) DMHBI-Ind (**26**, 469/539 nm), g) DMHBI-2Py (**28**, 464/618 nm), h) DMHBI-3Py (**29**, 467/613 nm), i) DMHBI-4Py (**30**, 470/–, no signal was obtained at this or any other wavelength), j) DMHBTI-Imi (**31**, black/red: 480/598 nm, blue/green: 420/541 nm), k) DMHBTI-Ind (**32**, 478/539 nm), l) DMHBI-Fc (**32**, 460/573 nm), m) DMHBI-Styr^{+} (**34**, 465/603 nm), n) DMHBO^{+} (**36**, 456/592 nm). o) The blank-corrected emission spectrum of Chili–DMHBI-Imi (**25**) was deconvoluted with two Gaussian peaks that are centered at 545 and 594 nm, respectively.

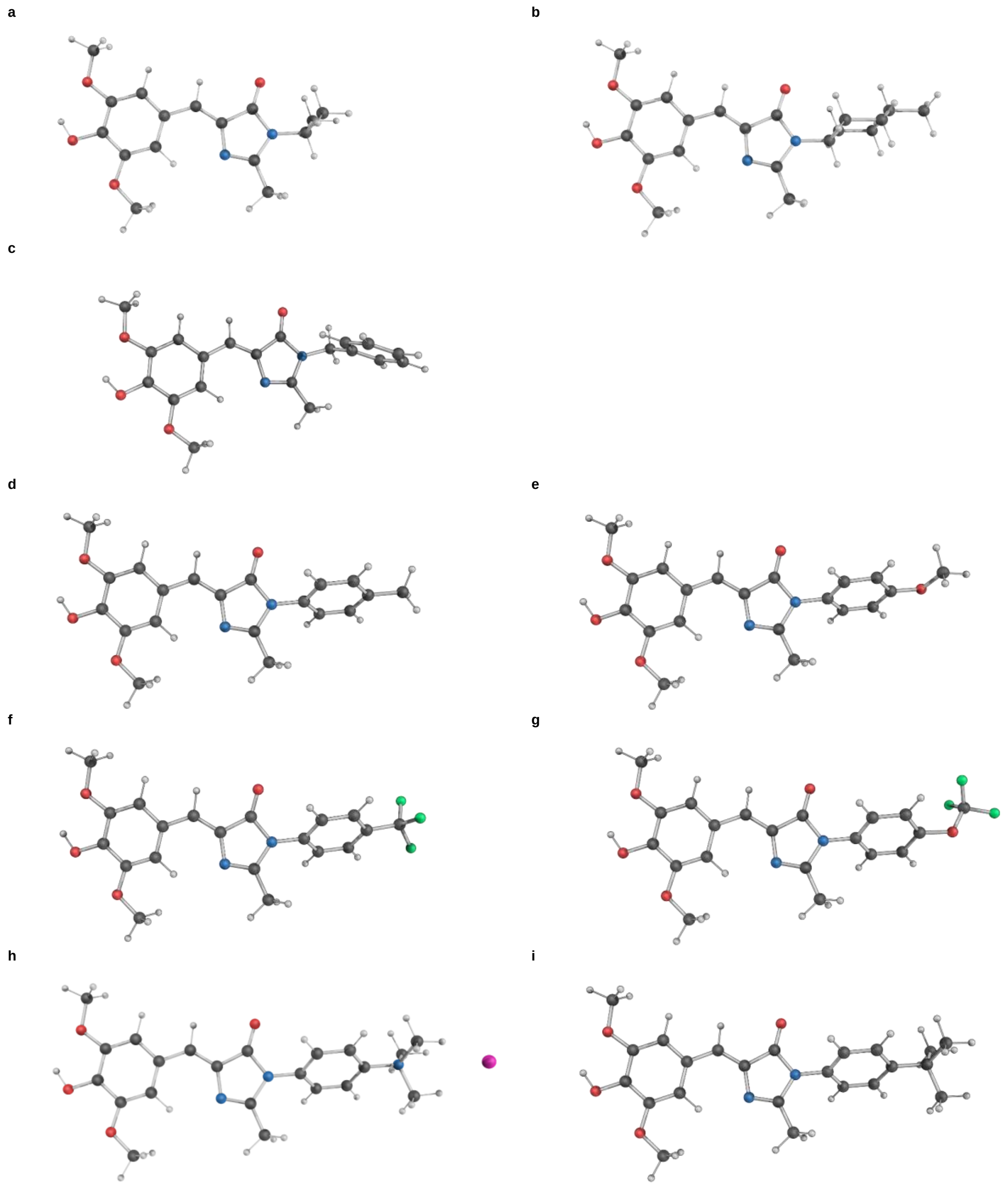

Supplementary Figure 4. DFT-optimized structures of a) DMHBI-*i*Pr (**3**), b) DMHBI-MeCy (**5**), c) DMHBI-Bn (**6**), d) DMHBTI (**9**), e) DMHBAI (**10**), f) DMHBTI^{F} (**11**), g) DMHBAI^{F} (**12**), h) DMHBI^{+} (**14**) and i) DMHBI^{C} (**15**) in the gas phase (B3LYP-D3/def2-TZVP).

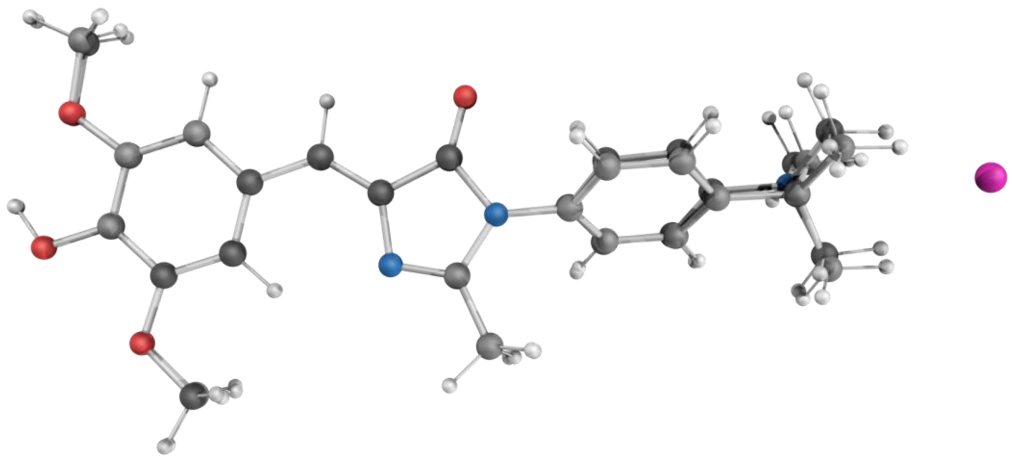

Supplementary Figure 5. Structural alignment of DMHBI$^+$ (**14**) and DMHBI^C (**15**). There is minimal deviation between the two molecules in the gas phase.

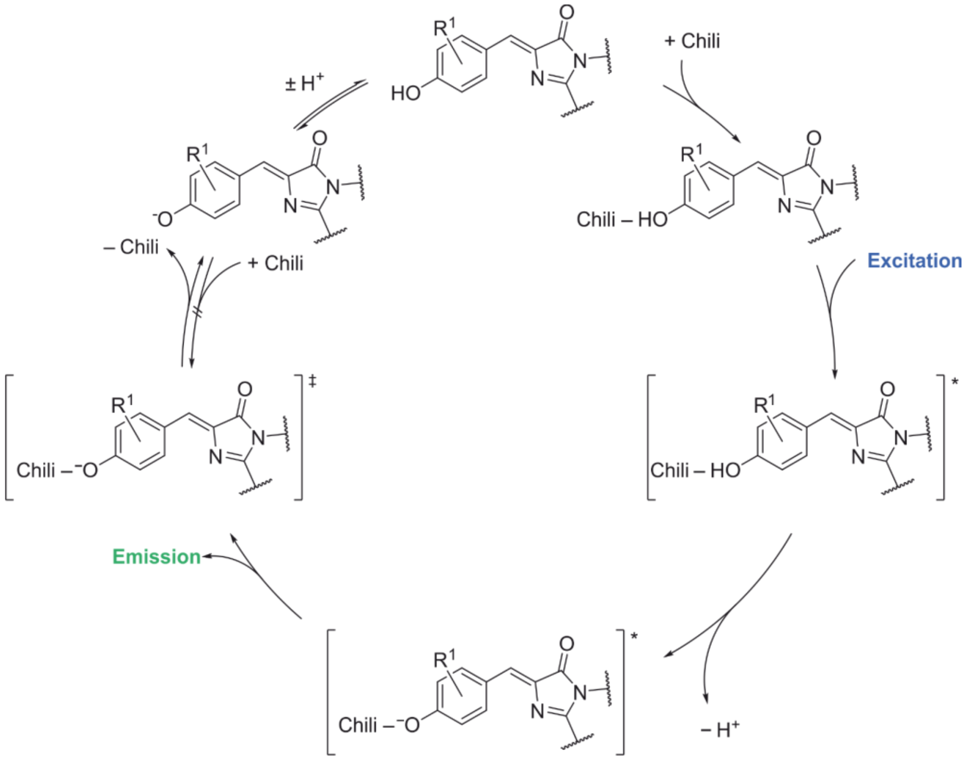

Supplementary Scheme 1. Proton transfer cycle for the binding and fluorescence activation of HBI dyes by the Chili aptamer. Only neutral HBI phenols can bind to the RNA. Upon excitation, proton loss is followed by fluorescence emission and subsequent dissociation of the ligand.

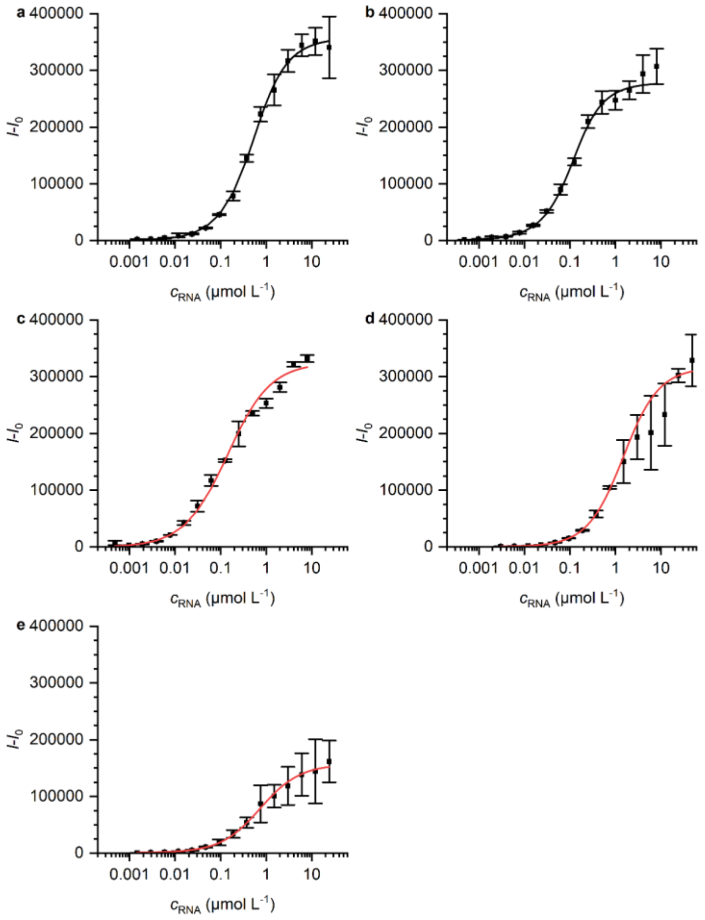

Supplementary Figure 6. Fluorescence titration curves of Chili with various HBI derivatives. The data points were fitted with either a one-site-binding model (black) or the Hill equation (red) in case of poor convergence for the first model. a) DMHBTI (**9**, c_{dye} = 0.3 µM, c_{RNA} up to 24 µM, K_D = 0.377±0.024 µM). b) DMHBAI (**10**, c_{dye} = 0.1 µM, c_{RNA} up to 8 µM, K_D = 0.065±0.007 µM). c) DMHBTI^F (**11**, c_{dye} = 0.1 µM, c_{RNA} up to 8 µM, K_{Hill} = 0.141±0.005 µM). d) DMHBAI^F (**12**, c_{dye} = 0.5 µM, c_{RNA} up to 48 µM, K_{Hill} = 1.47±0.15 µM). e) DMHBI^C (**15**, c_{dye} = 0.5 µM, c_{RNA} up to 24 µM, K_{Hill} = 0.74±0.07 µM).

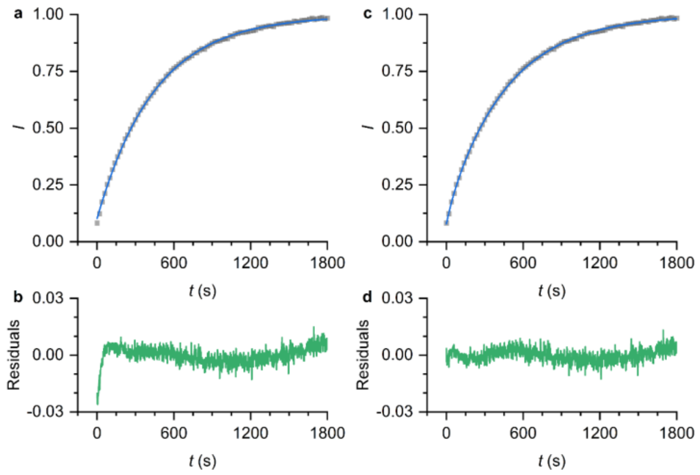

Supplementary Figure 7. Fluorescence activation kinetics of Chili with DMHBAI^F (**12**) under pseudo-first order conditions (0.025 µM RNA, 2 µM dye, 125 mM KCl, 5 mM $MgCl_2$, 40 mM HEPES pH 7.5). Data points were collected at 2 s intervals, every 10th point is plotted. Fit curves (blue) and residuals (green) are shown for a monoexponential (a, b) and a biexponential (c, d) association model. The second exponential term is needed to fully describe the initial behavior.

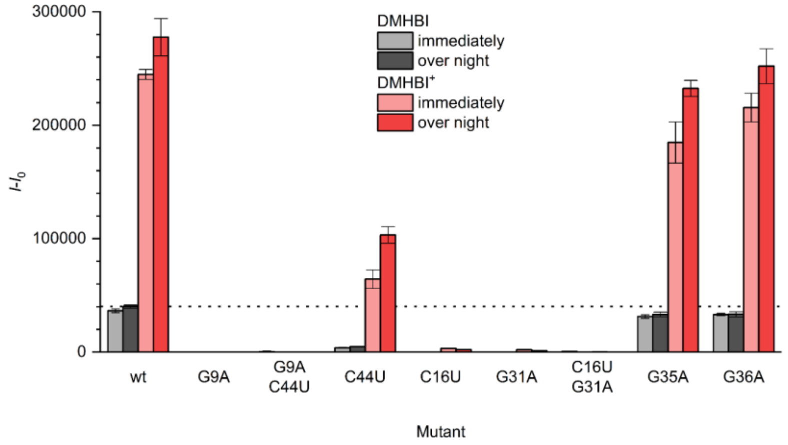

Supplementary Figure 8. Integrated fluorescence emission intensities for a number of Chili mutants with DMHBI and DMHBI$^+$ (0.5 µM RNA, 0.5 µM dye, 125 mM KCl, 5 mM MgCl2, 80 mM HEPES pH 7.5). The samples were excited at 400 nm (DMHBI) or 413 nm (DMHBI$^+$). Spectra were measured after an incubation time of 3 min and then again after 24 h.

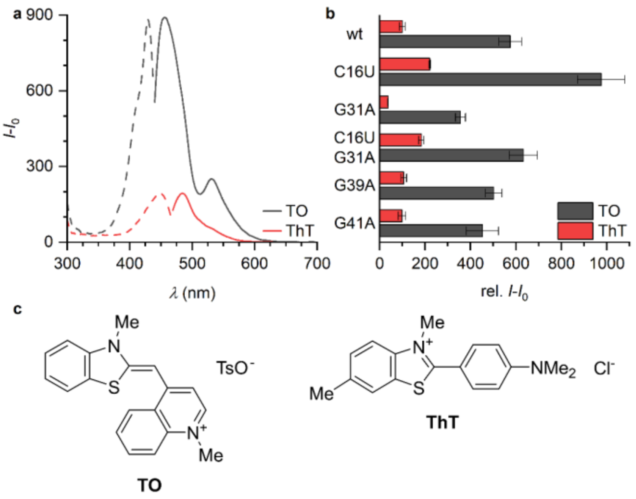

Supplementary Figure 9. a) Fluorescence excitation (dashed) and emission (solid) spectra of Thiazole orange (TO) and Thioflavin T (ThT) with wt-Chili (0.5 µM RNA, 0.5 µM dye, 125 mM KCl, 5 mM $MgCl_2$, 80 mM HEPES pH 7.5). Samples without added RNA were used for the blank correction. b) Integrated fluorescence emission intensities for a number of Chili mutants with TO and ThT. The samples were excited at 430 nm (TO) or 449 nm (ThT). c) Chemical structures of TO and ThT

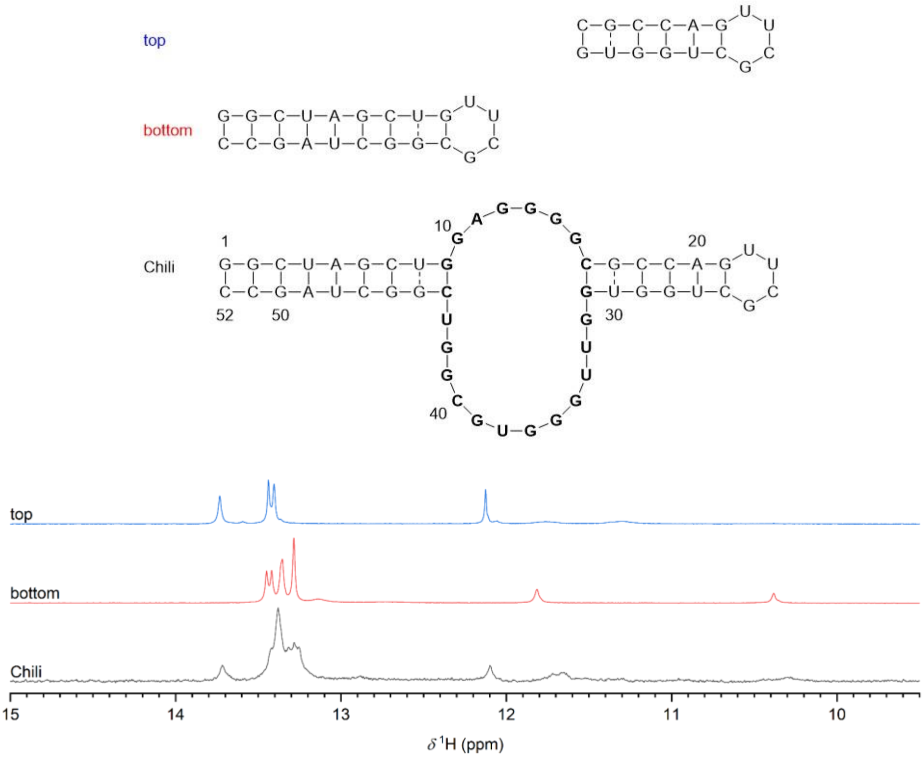

Supplementary Figure 10. ^{1}H NMR spectra of Chili (black, 150 µM) as well as the bottom (red, 460 µM) and top (blue, 400 µM) stem loop constructs in buffer (50 mM KCl, 1 mM MgCl$_2$, 25 mM Tris pH 7.4, 10% D$_2$O / 90% H$_2$O).

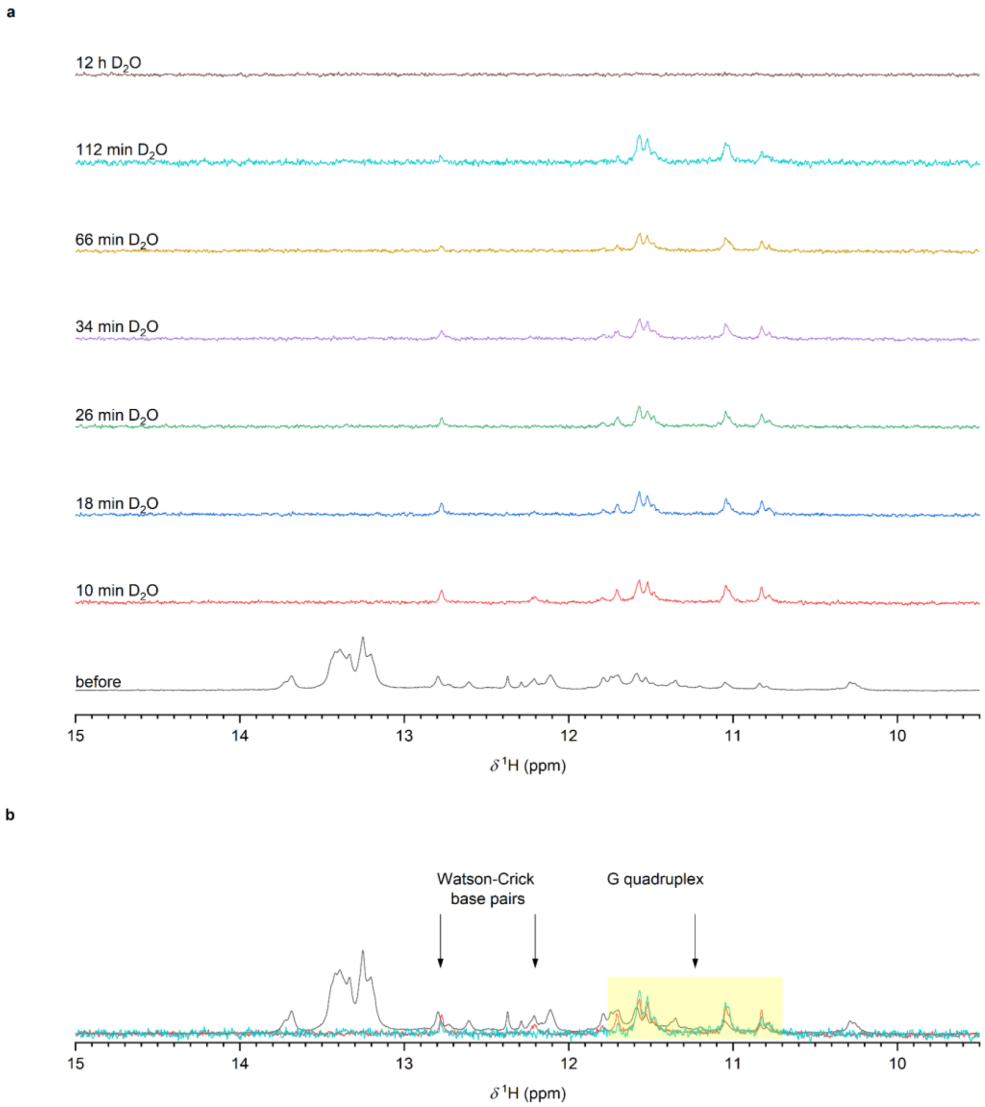

Supplementary Figure 11. ^{1}H NMR spectra of Chili–DMHBI^{+} (140 µM) in buffer (50 mM KCl, 1 mM $MgCl_2$, 25 mM Tris pH 7.4). a) Time course before and after transfer from 10% D_2O / 90% H_2O into pure D_2O. b) Spectra before the transfer (black), after 10 min (red) and after 112 min (turquoise).

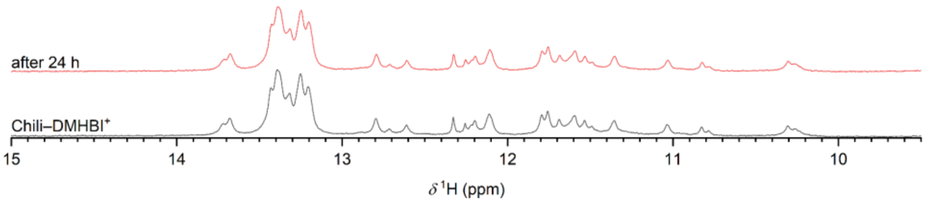

Supplementary Figure 12. ^{1}H NMR spectra of Chili–DMHBI^{+} (150 µM) in buffer (50 mM KCl, 1 mM $MgCl_2$, 25 mM Tris pH 7.4, 10% D_2O / 90% H_2O) as shown in Figure 8a of the manuscript (black) and 24 h later (red).

5 NMR spectra

NMR spectra of all newly synthesized compounds are available in an additional supplementary document.

6 Cartesian coordinates of HBI derivatives

6.1 (*Z*)-5-(4-Hydroxy-3,5-dimethoxybenzylidene)-3-isopropyl-2-methyl-3,5-dihydro-4*H*-imidazol-4-one (DMHBI-*i*Pr, 3)

H	-0.448689	-2.367131	1.709123
C	-0.397167	-1.333419	2.008131
C	-1.532747	-0.755308	2.597711
C	-1.493672	0.592515	2.992410
H	-2.367475	1.035651	3.446754
C	-0.343127	1.334716	2.793171
O	-0.175083	2.654198	3.125841
C	0.790118	0.758359	2.209874
C	0.755938	-0.587742	1.816879
O	1.899068	-1.062418	1.261264
O	1.916006	1.485181	2.022422
C	-1.279767	3.348851	3.682209
C	1.925240	-2.426437	0.872802
H	1.738661	2.378410	2.350859
H	-0.944193	4.370220	3.844518
H	-1.582690	2.907788	4.635779
H	-2.130079	3.347777	2.994789
H	2.921441	-2.602199	0.473908
H	1.178292	-2.635805	0.101750
H	1.749903	-3.086167	1.727402
C	-3.061413	-2.769379	2.502569
C	-4.382369	-3.366097	2.821099
N	-2.276346	-3.728227	1.850250
N	-4.270266	-4.669167	2.314619
C	-3.003369	-4.798570	1.760301
O	-5.356871	-2.897925	3.379429
C	-2.750953	-1.492687	2.821450
H	-3.552286	-0.954607	3.318465
C	-5.322517	-5.687817	2.384953
C	-5.645060	-6.035061	3.837968
C	-2.541549	-6.058262	1.121351
H	-1.528290	-5.908580	0.757277
H	-3.183817	-6.337701	0.282380
H	-2.545883	-6.890074	1.830298
H	-4.907156	-6.574867	1.905597
H	-6.046619	-5.166090	4.356985
H	-4.749000	-6.370851	4.362609
C	-6.558132	-5.249808	1.598698
H	-7.303597	-6.046875	1.604981
H	-6.298892	-5.030110	0.561628
H	-6.996355	-4.357481	2.043024
H	-6.384500	-6.836954	3.873386

6.2 (*Z*)-5-(4-Hydroxy-3,5-dimethoxybenzylidene)-2-methyl-3-(*trans*-4-methylcyclohexyl)-3,5-dihydro-4*H*-imidazol-4-one (DMHBI- MeCy, 5)

H	-0.434333	-2.339495	1.672485
C	-0.391253	-1.316625	2.007506
C	-1.536030	-0.765096	2.604718
C	-1.507654	0.568497	3.046132
H	-2.387935	0.991875	3.506776
C	-0.358575	1.322351	2.884515
O	-0.200287	2.630633	3.263347
C	0.784135	0.771514	2.295031
C	0.760587	-0.560130	1.855462
O	1.912596	-1.011369	1.298482
O	1.909315	1.508688	2.147098
C	-1.309415	3.297634	3.843661
C	1.944922	-2.357363	0.851988
H	1.724170	2.389371	2.503815
H	-0.978842	4.313231	4.047394
H	-1.612731	2.817562	4.778082
H	-2.158009	3.320295	3.154473
H	2.944971	-2.514166	0.454756
H	1.205087	-2.534368	0.066093
H	1.763475	-3.053730	1.675704
C	-3.048953	-2.784094	2.421502
C	-4.356442	-3.416783	2.728968

N	-2.255093	-3.701252	1.721754
N	-4.228841	-4.694795	2.164067
C	-2.964786	-4.779263	1.594881
O	-5.329527	-2.991486	3.322989
C	-2.749953	-1.519571	2.795709
H	-3.553689	-1.010894	3.319041
C	-5.237316	-5.750395	2.241919
C	-6.544607	-5.339923	1.558023
C	-5.475564	-6.187062	3.690704
C	-7.579228	-6.461540	1.646719
C	-7.837341	-6.903614	3.091304
C	-6.517611	-7.302273	3.760234
C	-8.864821	-8.029557	3.159372
C	-2.489399	-6.002228	0.897502
H	-1.486243	-5.816792	0.522027
H	-3.142033	-6.260705	0.059916
H	-2.464241	-6.861823	1.571996
H	-9.060656	-8.325601	4.192445
H	-8.508167	-8.912842	2.621659
H	-9.813952	-7.727891	2.710941
H	-6.924268	-4.438937	2.042125
H	-6.342303	-5.085708	0.514326
H	-7.234062	-7.329522	1.070370
H	-8.514554	-6.135852	1.184170
H	-8.235983	-6.038654	3.636041
H	-6.129227	-8.201756	3.265512
H	-6.695585	-7.576340	4.803406
H	-4.826770	-6.603566	1.696979
H	-5.812845	-5.321210	4.263371
H	-4.529591	-6.516469	4.128400

6.3 (*Z*)-3-Benzyl-5-(4-hydroxy-3,5-dimethoxybenzylidene)-2-methyl-3,5-dihydro-4*H*-imidazol-4-one (DMHBI-Bn, **6**)

H	-2.381237	-2.503561	-1.500380
C	-1.396059	-2.073025	-1.431822
C	-0.708441	-2.176356	-0.211872
C	0.579251	-1.625997	-0.097511
H	1.106289	-1.706798	0.841610
C	1.155911	-0.991726	-1.183273
O	2.401476	-0.419721	-1.205336
C	0.470645	-0.884945	-2.398193
C	-0.815529	-1.433227	-2.516440
O	-1.403430	-1.283623	-3.729446
O	1.034881	-0.259854	-3.457333
C	3.172540	-0.452503	-0.014662
C	-2.700783	-1.830783	-3.904431
H	1.910083	0.045411	-3.178041
H	4.106720	0.053856	-0.244634
H	3.380185	-1.481666	0.290692
H	2.662395	0.072019	0.797815
H	-2.980256	-1.608437	-4.931481
H	-3.421288	-1.373720	-3.220292
H	-2.699369	-2.913473	-3.749484
C	-2.460949	-3.446493	1.102785
C	-2.861355	-4.073788	2.386898
N	-3.491648	-3.625361	0.168749
N	-4.137324	-4.578241	2.099811
C	-4.430168	-4.283262	0.774213
O	-2.288575	-4.176610	3.453696
C	-1.267987	-2.830226	0.944307
H	-0.646179	-2.840302	1.834216
C	-4.911062	-5.374193	3.028385
C	-5.714259	-4.695203	0.155610
H	-5.732413	-4.360593	-0.878462
H	-6.562388	-4.257475	0.689123
H	-5.832963	-5.780395	0.190674
H	-4.421279	-5.244482	3.996479
H	-5.921352	-4.969286	3.114075
C	-4.970660	-6.842223	2.666822
C	-6.133467	-7.573090	2.895520
C	-3.862212	-7.489471	2.123837
C	-6.190469	-8.928703	2.591548
C	-3.918207	-8.842241	1.814631

C	-5.082313	-9.566829	2.047907
H	-7.004012	-7.077656	3.310215
H	-2.952052	-6.932734	1.941311
H	-7.102398	-9.483358	2.772762
H	-3.049999	-9.332125	1.392357
H	-5.125071	-10.620781	1.805064

6.4 (*Z*)-5-(4-Hydroxy-3,5-dimethoxybenzylidene)-2-methyl-3-(4-methylphenyl)-3,5-dihydro-4*H*-imidazol-4-one (DMHBTI, 9)

H	-0.506210	-2.330653	1.784354
C	-0.427988	-1.287219	2.039794
C	-1.563205	-0.641569	2.555447
C	-1.486123	0.719142	2.896729
H	-2.360153	1.214545	3.293476
C	-0.298298	1.406347	2.722359
O	-0.090092	2.729069	3.015454
C	0.832662	0.764404	2.207046
C	0.759839	-0.594095	1.864918
O	1.903173	-1.133851	1.373039
O	1.993449	1.438631	2.036453
C	-1.174262	3.475652	3.545508
C	1.880237	-2.505620	1.011700
H	1.843894	2.347840	2.333601
H	-0.798692	4.483712	3.703102
H	-1.510778	3.058069	4.497987
H	-2.012769	3.503505	2.843890
H	2.880070	-2.733722	0.649877
H	1.150201	-2.695734	0.219577
H	1.646507	-3.139897	1.871412
C	-3.158092	-2.606840	2.521653
C	-4.520957	-3.126126	2.793726
N	-2.364303	-3.644612	2.017755
N	-4.407031	-4.491241	2.442049
C	-3.106850	-4.704871	1.985499
O	-5.520871	-2.582874	3.208824
C	-2.820957	-1.317788	2.752414
H	-3.631677	-0.713496	3.147771
C	-5.461539	-5.434883	2.527828
C	-6.663445	-5.188652	1.871183
C	-5.319429	-6.589631	3.289522
C	-7.701209	-6.103151	1.965494
C	-7.566763	-7.280997	2.702155
C	-6.359884	-7.505808	3.362765
H	-4.402824	-6.760678	3.837839
H	-6.235778	-8.402752	3.957989
H	-6.782291	-4.275456	1.305288
H	-8.635201	-5.897663	1.456168
C	-8.687037	-8.282451	2.767568
C	-2.655528	-6.027123	1.481922
H	-1.694692	-5.900112	0.989183
H	-3.380254	-6.446875	0.781444
H	-2.541421	-6.745418	2.296608
H	-8.613256	-8.903524	3.661067
H	-8.667046	-8.949552	1.900394
H	-9.659880	-7.788863	2.775123

6.5 (*Z*)-5-(4-Hydroxy-3,5-dimethoxybenzylidene)-3-(4-methoxyphenyl)-2-methyl-3,5-dihydro-4*H*-imidazol-4-one (DMHBAI, 10)

H	-0.558660	-2.340095	1.766989
C	-0.458043	-1.297350	2.018263
C	-1.559475	-0.642405	2.592387
C	-1.454181	0.718065	2.927699
H	-2.301685	1.220339	3.370276
C	-0.272329	1.396390	2.688321
O	-0.038095	2.718395	2.966265
C	0.825384	0.744727	2.116433
C	0.724577	-0.613689	1.780994
O	1.836554	-1.162792	1.231236
O	1.979906	1.410809	1.884329
C	-1.095131	3.481892	3.525035
C	1.797870	-2.543177	0.906016
H	1.849603	2.322910	2.181507
H	-0.707398	4.490193	3.648222

H	-1.395999	3.084416	4.498221
H	-1.960575	3.501882	2.857099
H	2.780884	-2.781338	0.506836
H	1.034097	-2.753889	0.152006
H	1.602511	-3.154301	1.791756
C	-3.171119	-2.593532	2.634029
C	-4.526409	-3.099396	2.965386
N	-2.414215	-3.636273	2.084812
N	-4.447395	-4.460256	2.592826
C	-3.173318	-4.685501	2.074315
O	-5.499209	-2.547474	3.430928
C	-2.810909	-1.308881	2.852971
H	-3.595411	-0.698989	3.290067
C	-5.512343	-5.390854	2.704020
C	-6.708359	-5.156085	2.041302
C	-5.383374	-6.528976	3.499524
C	-7.765787	-6.052232	2.148677
C	-7.622599	-7.203735	2.921892
C	-6.422771	-7.434864	3.599270
H	-4.466831	-6.696294	4.049411
H	-6.332485	-8.323807	4.208796
H	-6.818774	-4.260072	1.446625
H	-8.687473	-5.842532	1.627360
O	-8.587801	-8.147685	3.080960
C	-9.816767	-7.974949	2.394441
H	-9.664628	-7.937840	1.311671
H	-10.330658	-7.065558	2.719511
H	-10.424170	-8.841325	2.644611
C	-2.767040	-6.010067	1.540712
H	-1.806852	-5.904509	1.041803
H	-3.510062	-6.390654	0.836934
H	-2.672653	-6.749509	2.338714

6.6 (*Z*)-5-(4-Hydroxy-3,5-dimethoxybenzylidene)-3-(4-trifluoromethylphenyl)-2-methyl-3,5-dihydro-4*H*-imidazol-4-one (DMHBTI^{F}, **11**)

H	-0.473878	-2.336531	1.761483
C	-0.415882	-1.293484	2.024396
C	-1.598361	-0.631901	2.395715
C	-1.547387	0.728911	2.744095
H	-2.457482	1.235746	3.028884
C	-0.337227	1.398532	2.730007
O	-0.147249	2.714610	3.058950
C	0.841760	0.739164	2.364623
C	0.794149	-0.616787	2.005424
O	1.984631	-1.167984	1.663287
O	2.025020	1.392116	2.358037
C	-1.274031	3.470120	3.476345
C	1.999488	-2.542644	1.312082
H	1.853061	2.303427	2.636569
H	-0.902525	4.465853	3.705371
H	-1.729174	3.034707	4.370020
H	-2.021327	3.532959	2.680688
H	3.036757	-2.780126	1.089048
H	1.380239	-2.734489	0.431236
H	1.649038	-3.167301	2.138606
C	-3.197159	-2.589523	2.239639
C	-4.572069	-3.100588	2.433558
N	-2.363929	-3.646298	1.855364
N	-4.422655	-4.492651	2.190806
C	-3.086900	-4.718721	1.842599
O	-5.604829	-2.545059	2.734828
C	-2.875648	-1.293745	2.460610
H	-3.718242	-0.675660	2.754536
C	-5.455240	-5.438008	2.371220
C	-6.711028	-5.201663	1.814409
C	-5.244344	-6.578933	3.143823
C	-7.742217	-6.103281	2.026578
C	-7.524820	-7.249853	2.784138
C	-6.272277	-7.486810	3.340401
H	-4.284321	-6.744250	3.610664
H	-6.103347	-8.371588	3.938132
H	-6.876639	-4.306876	1.233878
H	-8.715968	-5.917355	1.595635

C	-8.656453	-8.196648	3.066503
C	-2.580746	-6.056273	1.438629
H	-1.630045	-5.921829	0.928146
H	-3.286230	-6.562790	0.778128
H	-2.418760	-6.704292	2.302311
F	-8.228972	-9.468245	3.218005
F	-9.580010	-8.198998	2.084185
F	-9.307027	-7.870848	4.208706

6.7 (*Z*)-5-(4-Hydroxy-3,5-dimethoxybenzylidene)-3-(4-trifluoromethoxyphenyl)-2-methyl-3,5-dihydro-4*H*-imidazol-4-one (DMHBAI^{F}, **12**)

H	-0.640484	-2.087243	2.645256
C	-0.652919	-1.014190	2.554148
C	-1.896176	-0.368775	2.452960
C	-1.938233	1.030368	2.328339
H	-2.894636	1.525501	2.248740
C	-0.760036	1.754730	2.304788
O	-0.661823	3.115559	2.181952
C	0.479091	1.112484	2.408553
C	0.525326	-0.284445	2.533592
O	1.767190	-0.821156	2.624269
O	1.629735	1.821891	2.387057
C	-1.860079	3.862820	2.040311
C	1.868478	-2.231980	2.738376
H	1.391291	2.755295	2.290142
H	-1.558534	4.902794	1.943307
H	-2.500862	3.748613	2.918764
H	-2.410067	3.556547	1.146379
H	2.932574	-2.449622	2.787749
H	1.430264	-2.732957	1.870481
H	1.376752	-2.593682	3.645946
C	-3.381713	-2.414228	2.544703
C	-4.750836	-2.977797	2.529106
N	-2.455642	-3.460718	2.630158
N	-4.513452	-4.371069	2.642009
C	-3.129857	-4.563159	2.682619
O	-5.839475	-2.454208	2.441941
C	-3.146325	-1.084213	2.464694
H	-4.044948	-0.479419	2.394261
C	-5.528222	-5.355702	2.647366
C	-6.494095	-5.350072	1.643008
C	-5.579493	-6.316733	3.653922
C	-7.488993	-6.316056	1.627823
C	-7.509520	-7.278984	2.624452
C	-6.566455	-7.291829	3.637490
H	-4.856205	-6.295392	4.456642
H	-6.616624	-8.050443	4.405911
H	-6.462904	-4.588057	0.878416
H	-8.236062	-6.329015	0.847522
O	-8.450529	-8.319657	2.584392
C	-9.709615	-8.055603	2.994667
C	-2.514211	-5.913624	2.738293
H	-1.459427	-5.819676	2.491765
H	-2.998394	-6.597807	2.039499
H	-2.599730	-6.351902	3.735023
F	-9.752463	-7.549339	4.239609
F	-10.390250	-9.199379	2.978058
F	-10.343146	-7.176733	2.194054

6.8 (DMHBI^{+}, **14**)

H	-0.13268	-0.90426	-10.07804
C	-0.33735	-0.12469	-10.79201
C	-0.69361	1.14606	-10.30983
C	-0.96319	2.18119	-11.2226
H	-1.23771	3.1569	-10.85
C	-0.86658	1.94175	-12.58103
O	-1.09102	2.85776	-13.57263
C	-0.51251	0.6749	-13.06183
C	-0.24942	-0.36325	-12.15378
O	0.08275	-1.55316	-12.70958
O	-0.42173	0.44008	-14.3875
C	-1.42991	4.18427	-13.19653
C	0.35399	-2.6372	-11.83464

H	-0.63101	1.26784	-14.84469
H	-1.54711	4.74017	-14.12321
H	-0.63558	4.63615	-12.59655
H	-2.36837	4.20574	-12.63609
H	0.59184	-3.48269	-12.47526
H	-0.51707	-2.87643	-11.21806
H	1.2049	-2.41785	-11.18338
C	-0.53216	0.65826	-7.83302
C	-0.68407	1.14125	-6.44606
N	-0.0892	-0.66977	-7.81219
N	-0.25599	0.02104	-5.68
C	0.06606	-1.00691	-6.57541
O	-1.0636	2.19687	-5.98911
C	-0.79355	1.44264	-8.90628
H	-1.11833	2.44655	-8.65121
C	-0.20726	1.46E-4	-4.27378
C	-1.30923	0.42093	-3.53742
C	0.94575	-0.40321	-3.60145
C	-1.27206	0.41396	-2.14899
C	-0.13264	-0.0187	-1.48721
C	0.97858	-0.42544	-2.21814
H	1.8261	-0.68561	-4.16026
H	1.88321	-0.7484	-1.72478
H	-2.19551	0.76243	-4.05044
H	-2.14512	0.74384	-1.61058
N	-0.06381	-0.06775	0.00112
C	-1.31294	0.43688	0.6725
H	-2.15093	-0.18676	0.3749
H	-1.47523	1.47155	0.38384
H	-1.14278	0.35854	1.7507
C	0.14174	-1.49602	0.45789
H	-0.69229	-2.08614	0.08663
H	0.17727	-1.48693	1.55228
H	1.07572	-1.86482	0.04663
C	0.48918	-2.35802	-6.1253
H	0.42464	-3.03576	-6.97296
H	-0.14529	-2.72319	-5.31551
H	1.51903	-2.35913	-5.76168
C	1.08201	0.78341	0.50333
H	0.92364	1.79819	0.14738
H	2.01381	0.38862	0.11277
H	1.07246	0.72862	1.59636
I	0.25814	-0.31276	3.99704

6.9 (*Z*)-3-(4-*tert*-Butylphenyl)-5-(4-hydroxy-3,5-dimethoxybenzylidene)-2-methyl-3,5-dihydro-4*H*-imidazol-4-one (DMHBI^{C}, **15**)

H	-0.565099	-2.329345	1.741030
C	-0.460734	-1.292754	2.014554
C	-1.545866	-0.657948	2.640223
C	-1.436087	0.695281	3.002754
H	-2.270499	1.182889	3.484847
C	-0.267224	1.386270	2.736521
O	-0.031745	2.704148	3.031896
C	0.814805	0.753625	2.115105
C	0.710282	-0.597844	1.754401
O	1.806406	-1.129425	1.157418
O	1.957399	1.431310	1.859260
C	-1.076056	3.451832	3.634197
C	1.762686	-2.502953	0.804850
H	1.831641	2.336790	2.177869
H	-0.691518	4.461050	3.759806
H	-1.343678	3.038454	4.610432
H	-1.961951	3.474989	2.993727
H	2.732912	-2.728228	0.368628
H	0.975241	-2.701593	0.072251
H	1.599215	-3.133296	1.683598
C	-3.150039	-2.613658	2.660684
C	-4.487147	-3.146563	3.021855
N	-2.414560	-3.618073	2.019920
N	-4.419482	-4.484437	2.567094
C	-3.167830	-4.670101	1.980575
O	-5.438700	-2.629084	3.563850
C	-2.784023	-1.339222	2.926245

H	-3.549576	-0.752887	3.424773
C	-5.474151	-5.427988	2.656574
C	-6.717239	-5.136305	2.112144
C	-5.282820	-6.647894	3.299379
C	-7.745706	-6.068586	2.184598
C	-7.565677	-7.311657	2.790739
C	-6.311356	-7.574182	3.351458
H	-4.331210	-6.865930	3.765377
H	-6.130756	-8.516697	3.851011
H	-6.879914	-4.178617	1.637968
H	-8.699216	-5.808764	1.749363
C	-8.670554	-8.368331	2.869315
C	-9.955544	-7.922548	2.160961
H	-9.785852	-7.730322	1.099902
H	-10.375301	-7.021040	2.610595
H	-10.706575	-8.710371	2.239308
C	-8.182761	-9.668878	2.202264
H	-7.928996	-9.495827	1.154711
H	-8.965534	-10.429549	2.242879
H	-7.299960	-10.072490	2.698853
C	-2.777086	-5.952392	1.342037
H	-1.852784	-5.795838	0.791337
H	-3.557239	-6.304269	0.664321
H	-2.618000	-6.737150	2.084155
C	-9.003658	-8.645505	4.347386
H	-9.355212	-7.737974	4.841384
H	-8.133007	-9.009043	4.894311
H	-9.787714	-9.402406	4.422506

7 References

1. Rio, D.C. Expression and purification of active recombinant T7 RNA polymerase from E. coli, *Cold. Spring. Harb. Protoc.*

2. Fulmer, G.R., Miller, A.J.M., Sherden, N.H., Gottlieb, H.E., Nudelman, A., Stoltz, B.M., Bercaw, J.E. and Goldberg, K.I. (2010) NMR Chemical Shifts of Trace Impurities. Common Laboratory Solvents, Organics, and Gases in Deuterated Solvents Relevant to the Organometallic Chemist, *Organometallics,* **29,** 2176–2179.

3. Steinmetzger, C., Palanisamy, N., Gore, K.R. and Höbartner, C. (2019) A Multicolor Large Stokes Shift Fluorogen-Activating RNA Aptamer with Cationic Chromophores, *Chem. Eur. J.,* **25,** 1931–1935.

4. Yadav, V. and Babu, K.G. (2005) A Remarkably Efficient Markovnikov Hydrochlorination of Olefins and Transformation of Nitriles into Imidates by Use of AcCl and an Alcohol, *Eur. J. Org. Chem.,* **2005,** 452–456.

5. Baldridge, A., Kowalik, J. and Tolbert, L. (2010) Efficient Synthesis of New 4-Arylideneimidazolin-5-ones Related to the GFP Chromophore by 2+3 Cyclocondensation of Arylideneimines with Imidate Ylides, *Synthesis,* **2010,** 2424–2436.

6. D'Antona, N., Morrone, R., Gambera, G. and Pedotti, S. (2016) Enantiorecognition of planar "metallocenic" chirality by a nitrile hydratase/amidase bienzymatic system, *Org. Biomol. Chem.,* **14,** 4393–4399.

7. Hercouet, A. and Le Corre, M. (1988) Triphenylphosphonium Bromide: A Convenient and Quantitative Source of Gaseous Hydrogen Bromide, *Synthesis,* **1988,** 157–158.

8. Kalita, D., Morisue, M. and Kobuke, Y. (2006) Synthesis and electrochemical properties of slipped-cofacial porphyrin dimers of ferrocene-functionalized Zn-imidazolyl-porphyrins as potential terminal electron donors in photosynthetic models, *New J. Chem.,* **30,** 77–92.

9. Manoni, F. and Connon, S.J. (2014) Catalytic asymmetric Tamura cycloadditions, *Angew. Chem. Int. Ed.,* **53,** 2628–2632.

10. Sklenář, V. and Bax, A. (1987) Spin-echo water suppression for the generation of pure-phase two-dimensional NMR spectra, *Journal of Magnetic Resonance (1969),* **74,** 469–479.

11. Neese, F. (2012) The ORCA program system, *Wiley Interdiscip. Rev. Comput. Mol. Sci.,* **2,** 73–78.

12. Neese, F. (2018) Software update: the ORCA program system, version 4.0, *Wiley Interdiscip. Rev. Comput. Mol. Sci.,* **8,** e1327.

13. Grimme, S., Antony, J., Ehrlich, S. and Krieg, H. (2010) A consistent and accurate ab initio parametrization of density functional dispersion correction (DFT-D) for the 94 elements H-Pu, *J. Chem. Phys.,* **132,** 154104.

14. Grimme, S., Ehrlich, S. and Goerigk, L. (2011) Effect of the damping function in dispersion corrected density functional theory, *J. Comput. Chem.,* **32,** 1456–1465.

15. Schäfer, A., Horn, H. and Ahlrichs, R. (1992) Fully optimized contracted Gaussian basis sets for atoms Li to Kr, *J. Chem. Phys.,* **97,** 2571–2577.

16. Weigend, F. and Ahlrichs, R. (2005) Balanced basis sets of split valence, triple zeta valence and quadruple zeta valence quality for H to Rn: Design and assessment of accuracy, *Phys. Chem. Chem. Phys.,* **7,** 3297–3305.

17. Weigend, F. (2006) Accurate Coulomb-fitting basis sets for H to Rn, *Phys. Chem. Chem. Phys.,* **8,** 1057–1065.

18. Peterson, K.A., Figgen, D., Goll, E., Stoll, H. and Dolg, M. (2003) Systematically convergent basis sets with relativistic pseudopotentials. II. Small-core pseudopotentials and correlation consistent basis sets for the post- d group 16–18 elements, *J. Chem. Phys.,* **119,** 11113–11123.
